# Recycled Plastic Content Quantified through Aggregation-Induced Emission

**DOI:** 10.1021/acssuschemeng.2c03389

**Published:** 2022-09-13

**Authors:** Zoé O. G. Schyns, Thomas M. Bennett, Michael P. Shaver

**Affiliations:** † Department of Materials, School of Natural Sciences, University of Manchester, Manchester M13 9BL, U.K.; ‡ Sustainable Materials Innovation Hub, Henry Royce Institute, University of Manchester, Manchester M13 9BL, U.K.

**Keywords:** mechanical recycling, recycled content, aggregation induced enhanced emission, polymer processing, fluorescence, circular economy

## Abstract

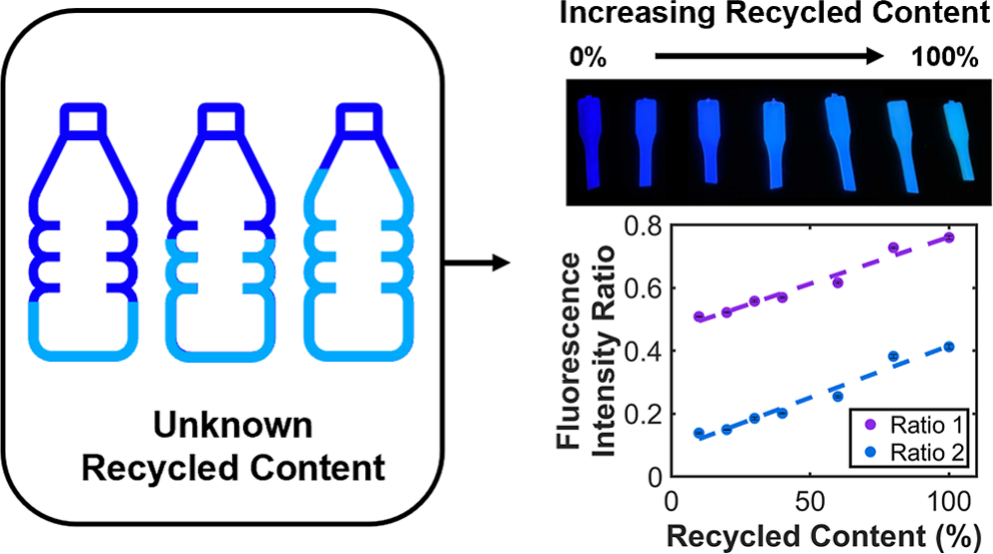

The linearity of the plastics economy is wasteful and polluting. To encourage recycling and decrease diversion to landfill, new legislation within the EU and UK will tax single-use plastic products made with less than 30% recycled plastic. At present, quantitative determination of recycled content is elusive and existing methods are inconsistent. We present a fluorescence-based analytical technique to determine recycled content in plastic and (single use) packaging. Bathochromic shifts resulting from aggregation of the fluorescent brightener 4,4′-bis(2-benzoxazolyl) stilbene (BBS) in three commodity plastics [high-density polyethylene, polypropylene, and poly(ethylene terephthalate)] at loadings ≤0.5 wt % were used to systematically quantify simulated recycled contents as low as 10 wt %. Linear correlations were found between recycled content and three fluorescence-based properties: emission, lifetime, and resulting color. We demonstrate how this multi-branched verification system is completely independent of sample dimensions and processing conditions, has a negligible effect on polymer properties, and is inexpensive and highly compatible with existing recycling infrastructure.

## Abstract 2

The aggregation of fluorescent molecules at low loadings enables the quantitative measurement of recycled content in plastics.

## Introduction

Plastic pollution resulting from increased plastic consumption and mismanagement is devastating natural environments. With a global landfill rate of 40%^1^ and plastic-related pollution being detected across all habitats around the globe, disruptive change is needed.^2−5^ The most energetically efficient pathway for plastics at end-of-life is mechanical recycling,^6^ and so, increasing recycling rates is essential. Yet recyclate use is disfavored due to increased cost relative to virgin feedstocks and diminished quality of material.^5^ Valorization of recycled content reduces greenhouse gas production, plastic loss to the environment, pressure on petrochemical feedstocks, and eventual cost of using recycled content, which remains a financial barrier to the circular economy.

Governments and global authorities are now responding to these societal concerns about fugitive plastics. Plastics both help and hinder achieving the UN’s Sustainable Development Goals, including target 12.5 which aims to dramatically increase recycling rates by 2030. Initiatives like the UK’s Plastic Packaging Tax (PPT), charging £200/tonne for single-use plastic packages with less than 30 wt % recycled content, aim to facilitate this change.^7,8^ Increasing recyclate, also known as post-consumer resin (PCR), incorporation will reduce mismanagement of plastic waste and can reduce greenhouse gas emissions by up to 15%.^9^ However, these ambitious legislations largely rely on the honesty and transparency of plastic product manufacturers and thus are susceptible to fraud or greenwashing.^10^

At present, the mass balance approach is used to track movement of recyclate feedstocks in and out of production facilities and across supply chains. This approach fails to identify true recycled content of the final product, is yet to be applicable to chemically recycled plastics, and is paperwork-heavy and financially taxing.^11,12^ Moreover, this approach’s loosely developed rules, as called for by key industry players, results in batch-to-batch or product-to-product recycled content variability, carbon-heavy cross-country recyclate transport, selective marketing, and the potential to further tarnish the credibility of the recycling industry.^10−12^

Alternative methods proposed for PCR content quantification rely on inconsistent comparisons of molecular weights (MWs) or molecular weight distributions of plastics before and after recycling. Gel permeation chromatography, mass spectrometry, rheology, and differential scanning calorimetry (DSC) can all provide MW estimates, varying in accuracy, but are heavily influenced by the processing conditions, polymer grade, additives, and resulting chemical reactions.^5,13^ Reliable comparisons of pre- and post-recycled polymer MWs would require standardization of polymer feedstock, processing conditions, and equipment across the industry. However, in a packaging sector dominated by trade-secret recipes and considerable variability, standardization remains impractical, and a process-independent PCR quantification method is non-existent.

4,4-Bis(2-benzoxazolyl) stilbene (BBS) is a Food and Drug Administration (FDA) and Registration, Evaluation, Authorisation and Restriction of Chemicals (REACH)-approved polymer additive with unique fluorescent properties and high thermal stability.^14−18^ BBS belongs to a class of molecules that exhibit aggregation-induced enhanced emission through an aggregation-induced bathochromic shift.^19−21^ At low concentrations, BBS exists as an isolated molecule which aggregates to form dimers with increasing concentration. These two BBS configurations, monomer and dimer, show detectable and distinct fluorescence emissions.^22−28^

The aggregation behavior of BBS is an example of strong coupling and can be explained by Kasha’s molecular exciton coupling theory.^29,30^ Formation of J-aggregates creates a low energy first excited state and a red-shifted emission spectrum.^29^ This fluorescence behavioral change has been used as a mechanical stress indicator in polypropylene (PP), poly(lactic acid) (PLA), poly(butylene succinate) (PBS), and low-density polyethylene (LDPE) films,^19,22−28^ and further developed as automated inspection equipment by Weder *et al.* in self-stress-sensing climbing equipment, construction, or packaging.^20,31−33^

**Figure 1:**
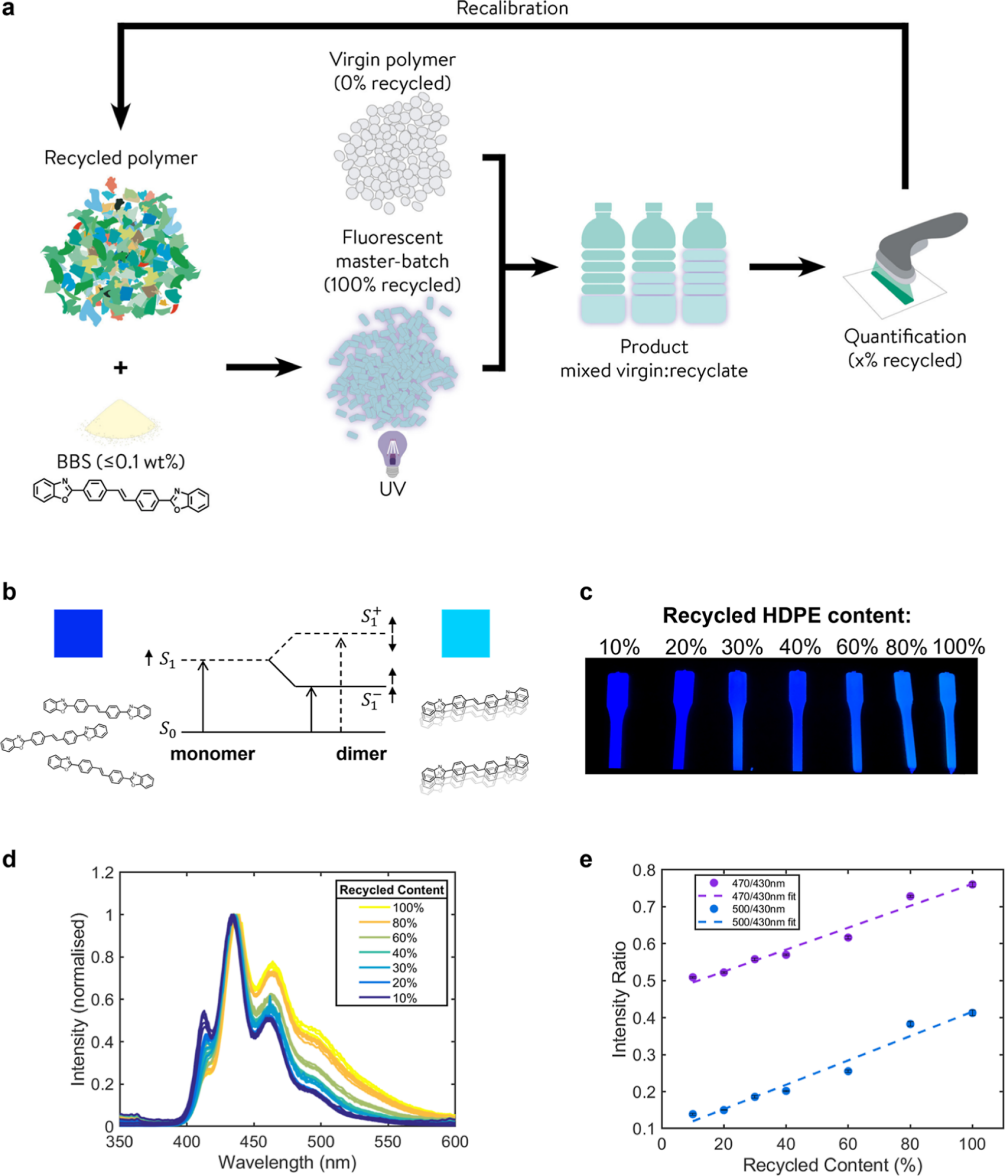
Overview of recycled content determination using fluorescence marking. (A) Graphic representation of the proposed recycled content marking technique. (B) Schematic representation of molecular exciton coupling theory applied to 4,4-bis(2-benzoxazolyl) stilbene (BBS) molecules. (C) Varying levels of recyclate content in high-density polyethylene (HDPE) marked using BBS. Illuminated at 365 nm. (D) Fluorescence emission spectra of varying recycled content for HDPE recyclate marked by BBS, normalized to 1 at the fluorescence emission maximum of isolated BBS molecules (430 nm). (E) Resulting intensity ratios between 470, 500, and 430 nm for 0.1 wt % BBS-HDPE MB. Error bars represent the standard error (*n* = 5), where each sample comes from the same batch. Fits were produced using the MATLAB curve fitting toolbox (470/430 nm *R*^2^ = 0.9696 and 500/430 nm *R*^2^ = 0.9648).

Here, we exploit this aggregation-induced-enhanced emission of BBS to create a novel yet practical methodology for PCR content quantification. Polymer master-batches loaded with BBS at concentrations ≤0.5 wt % were used to simulate a “tagged” PCR stream, which was then diluted with varying amounts of virgin polymer (Figure 1A). We demonstrate that linear correlations between recycled content and fluorescence emission ratios provide accurate predictions of recycled content in high-density polyethylene (HDPE), PP, and poly(ethylene terephthalate) (PET). Complimentary methods of recyclate determination were developed using fluorescence lifetimes and color values.

The ambiguity in PCR quantification that is inherent to the two currently available approaches—mass balance and polymer characterization—is avoided using fluorescent marking. Other fluorescence-based strategies have successfully been applied to distinguish the difference between food contact and non-food contact plastics,^34^ sort plastic by type,^35−38^ and for microplastic detection.^39^ This is the first and sole example of a process-independent, stable to additive, multi-pronged, fraud-resistant, and robust fluorescence-based method of tracking recycled content of a plastic feedstock and any resulting products. By developing a robust technique that avoids laborious effort and cost, it will encourage compliance over fraud and help rebuild public and corporate trust in recycling or be used for auditing of plastic packaging taxes being proposed worldwide.^7^

## Measurement of Recycled Content

### Recycled Content by Fluorescence Emission

HDPE was initially selected as a polymer matrix due to its ubiquity in both single-use packaging and mechanical recycling.^5,40^ BBS was dispersed directly in HDPE through melt extrusion at a range of concentrations (0.025–1.675 wt %) to determine the minimum concentration required for dye aggregation. The HDPE-BBS spectra were normalized to the fluorescence emission maximum of the isolated BBS molecules at 430 nm, corresponding to the 0 → 1 electronic transition (Figure 1B). In doing this, the observable changes in the emission spectra are attributed solely to dimer formation. A new dimer band was observed at 500 nm for BBS concentrations higher than 0.025 wt % (Figure S1); this emission band onset occurred at lower concentrations than those previously reported for BBS dispersed in PP, LDPE, PBS, and PLA (0.2–0.7 wt %),^24−26,28^ which we attribute to differences in crystallinity between these polymers and HDPE. Highly crystalline polymers such as HDPE (test grade: 73.3 ± 0.6%, Figure S37) enhance the nucleation rate of BBS by segregating BBS to the amorphous phase of the polymer, promoting dimer formation and subsequent fluorescence changes at lower dye loadings. A higher nucleation rate may also reduce the minimum concentration change required to disrupt any aggregates formed, lowering concentration thresholds.^19,22,32^ Recycling simulations were performed by creating a BBS-HDPE master-batch (MB) (0.1 wt % BBS relative to HDPE) by extrusion and diluting by reprocessing with virgin polymer to produce simulated recycled contents ranging from 0 to 100% (Figure 1C and Materials and Methods). The master-batch showed strong dimer-type emission traces with notable peaks at approximately 430, 470, and 500 nm, with some degree of aggregation observed for all diluted concentrations (Figure 1D). Explicitly, the BBS aggregation band at 500 nm increased in intensity by around 2.0-fold between 10 and 100% recycled content (Figure S12).

Comparisons of dimer content were performed by calculating fluorescence intensity ratios. Spectra were normalized as described above to remove monomer emission influence. To quantify PCR content, two different fluorescence intensity ratios were calculated using the peaks at 470, 500, and 430 nm (Figure 1E and eq 1).
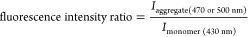
1

Comparison of ratios rather than intensities minimizes discrepancies between sample sizes and specific fluorimeter settings such as slit widths.^41^ Fluorescence intensity ratios were linear with varying recycled content, and this linearity was found at MB concentrations as low as 0.025 wt % (Figures S4 and S5). The BBS concentrations studied throughout this work did not cause any obvious color change under ambient lighting; the MB and any diluted material were visually indistinguishable from the virgin HDPE (Figure S6). The scaling of fluorescence intensity with BBS concentration was found to be non-linear at much higher concentrations (>0.5 wt %) (Figure S1) due to higher ordered structures that may induce aggregation-caused quenching (Figures S25–S27)*.*^25,42,43^ Using the 0.1 wt % MB, the linear correlations between recycled content were remarkably consistent, with an *R*^2^ value of 0.9635 for the 500 nm:430 nm and an *R*^2^ of 0.9681 for 470 nm:430 nm (Table S5). The high linearity between intensity ratio and recycled content of the 0.1 wt % MB paired with such cost-effective low dye loadings confirms that this concentration could be used in an industrial setting. The rest of this paper explores recycling simulations using a HDPE 0.1 wt % MB unless specified otherwise (Supporting Information Section S1.1.3).

### Verification of Recycled Content by Fluorescence Lifetimes

To prevent false positives in measuring recycled content, a multi-faceted quantification methodology is needed. Fluorescence lifetime measurements such as excitation wavelength and excitation duration are independent of measurement conditions and have greater molecular specificity than emission phenomena.^44^ Aggregated BBS molecules exhibit longer fluorescence lifetimes due to the symmetry-forbidden radiative transitions involved in their fluorescence.^24^ The nature of lifetime measurements and the unique properties of BBS allow for this secondary, precise measurement of recycled content through lifetime comparisons.

**Figure 2:**
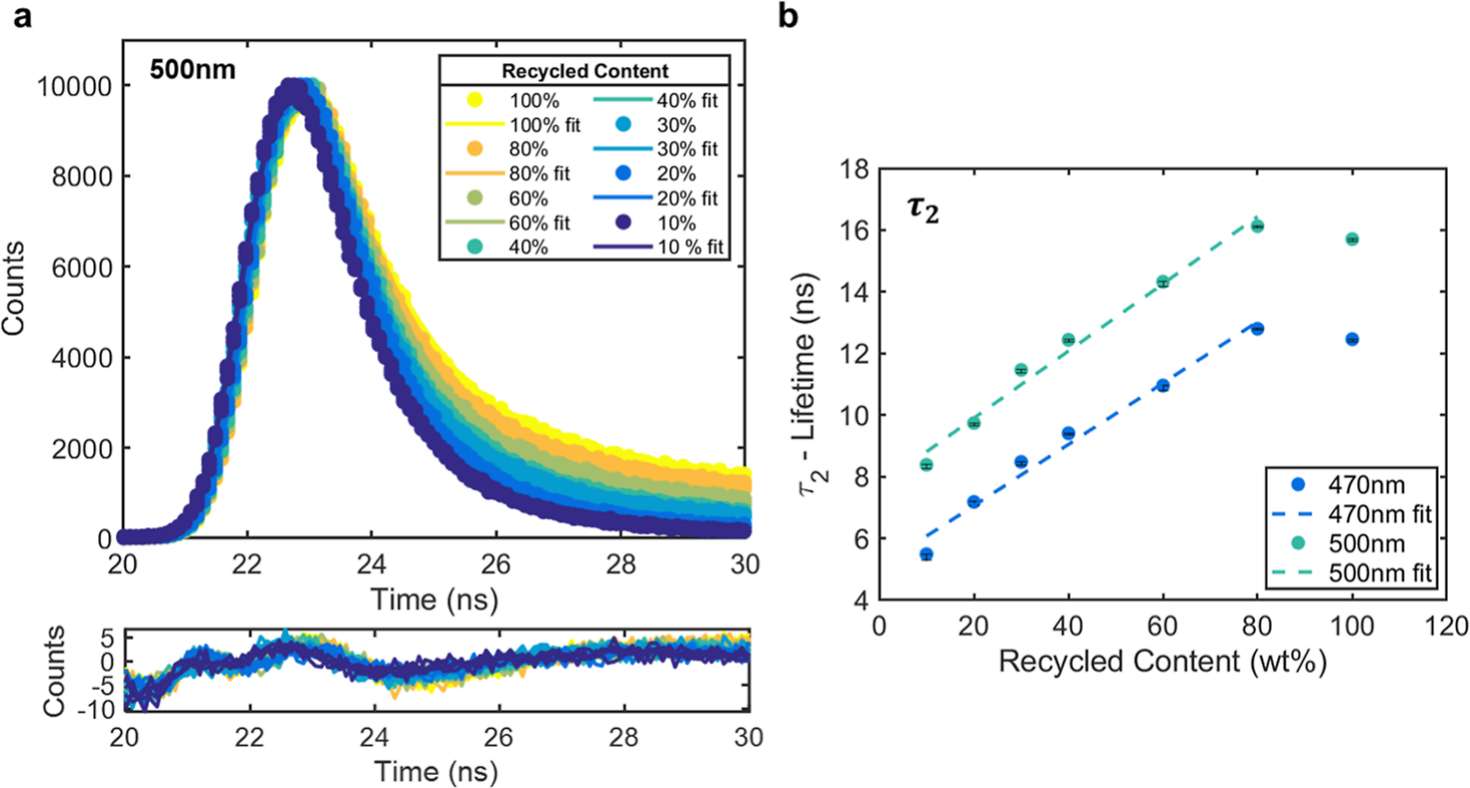
Fluorescence lifetime measurements for diluted 0.1 wt % BBS-HDPE MB. (A) Fluorescence lifetime emission traces with various recycled contents (●) and fitted with a bi-exponential decay function (—); samples were irradiated at 340 nm, and emission was measured at 500 nm. (B) Long-lived lifetime parameter (τ_2_) from bi-functional exponential function with increasing recycled content measured at 470 and 500 nm (excitation wavelength = 340 nm). Error bars represent the standard error (*n* = 5) where each sample comes from the same batch. Fits were produced using the MATLAB curve fitting toolbox (470 nm *R*^2^ = 0.9791, 500 nm *R*^2^ = 0.9839).

The initial fluorescence spectra revealed that dimer formation caused fluorescence emission shifts at 470 and 500 nm (Figure 1D). Fluorescence lifetime data were therefore collected at 470 and 500 nm at an excitation wavelength of 340 nm (Figure 2). The resulting data were indicative of dimer-type lifetimes, and all variants were fit using bi-functional exponential functions (Supporting Information Section S2.1.1). All samples showed evidence of two distinct decay times: one short-lived around 1–1.5 ns (τ_1_) attributed to the presence of monomeric BBS, and a second long-lived component of approximately 10 ns (τ_2_) consistent with the previously reported dimer decay times.^24^ All measured samples showed bi-functional decay curves, thus confirming some degree of dimerization at the lowest BBS concentration of the 10% recycled content sample (0.01 wt % BBS) (Figure 2). The contribution of τ_*2*_ increased with increasing recycled content for both 470 and 500 nm (Figure 2) as the ratio of dimer to monomer increased. At 470 nm, τ_2_ decreased from ∼12 ns at 100% recycled content to ∼5 ns at 10% recycled content, and from ∼16 ns at 100% recycled content to ∼8 ns at 10% recycled content at 500 nm; a similar decrease was also observed for τ_1_.

A second quantification method was realized through a linear trend for τ_2_ up to 80% recycled content, where a reduction in τ_2_ correlates with reduced recycled content (Figure 2). The τ_2_ data were highly linear for both 470 and 500 nm, with excellent reproducibility and *R*^2^ values of 0.9791 and 0.9839, respectively (10–80% recycled content). The loss of linearity in fluorescence lifetime trends for the 100% recycled content sample was tentatively attributed to aggregation-induced quenching, which could result from photon reabsorption, energy transfer, or another mechanism.^44^ Practically, measurement of fluorescence lifetimes is an additional process to validate recyclate content. Creating a multi-layered quantification process is essential to circumvent possible falsification of recyclate incorporation through doping with fluorophores that could enhance emission at 470 and 500 nm. This suggests a workflow of rapid fluorescence measurements (<1 min) for most audited samples coupled to additional audit checks for new suppliers or samples of questionable authenticity through fluorescence lifetimes (ca. 5 min); in both cases, these provide a step change in the time for analysis of recycled content.

### Recycled Content Determination for Quality Control

**Figure 3:**
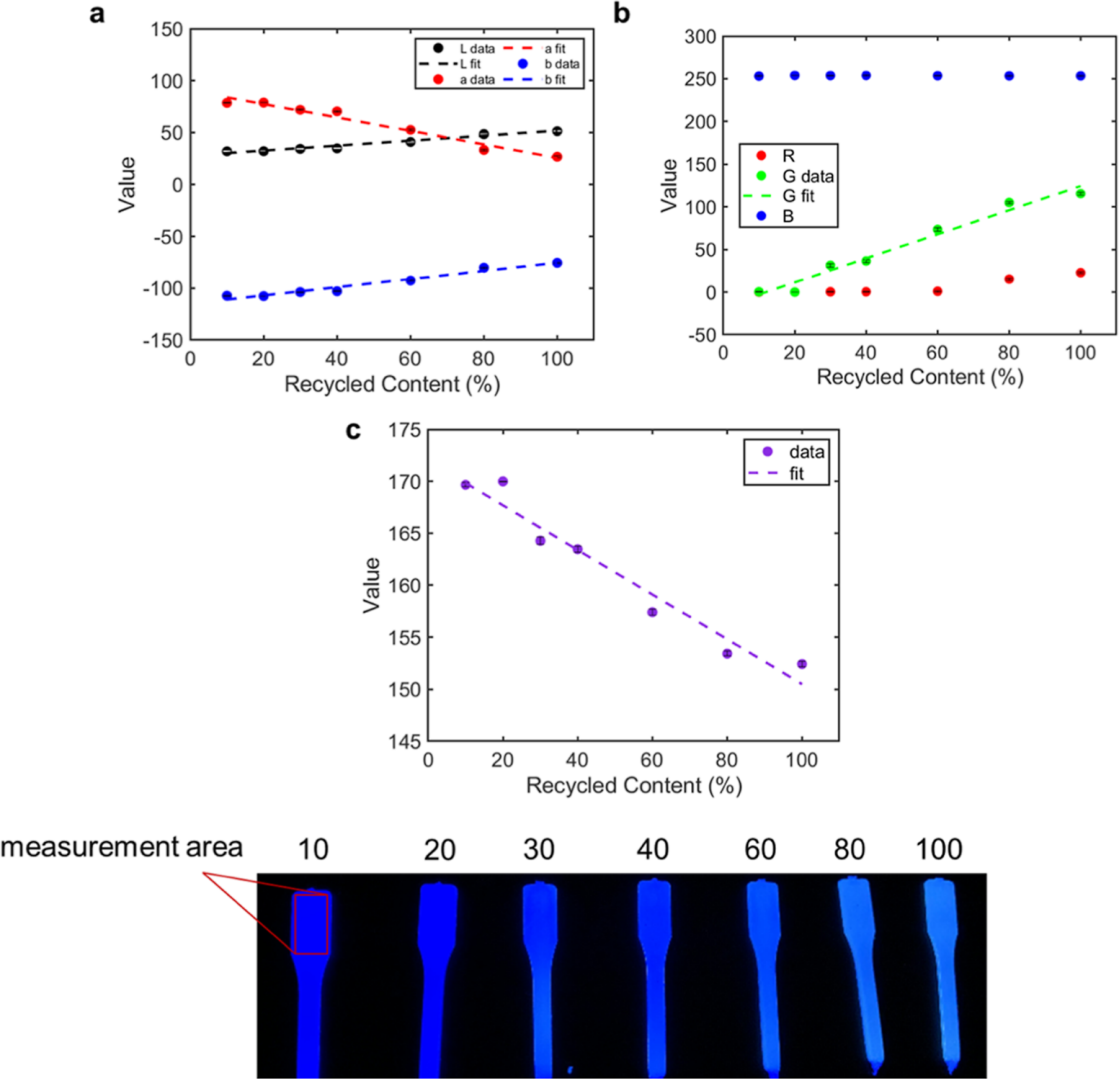
Color analysis of 0.1 wt % BBS-HDPE MB samples illuminated at 365 nm and photographed in a blacked-out room. (A) L*a*b* values were measured using ImageJ. Fits were produced using the MATLAB curve fitting toolbox [L (*R*^2^ = 0.9511), a (*R*^2^ = 0.9660), b (*R*^2^ = 0.9609)]. (B) RGB values were measured using ImageJ. Fits were produced using the MATLAB curve fitting toolbox [G (*R*^2^ = 0.9718)]. (C) Hue values were measured using ImageJ. Fits were produced using the MATLAB curve fitting toolbox [Hue (*R*^2^ = 0.9511)]. Errors were taken as the standard deviation produced by the ImageJ software.

The bathochromic shift resulting from increased dimer content increases the intensity of fluorescence emission in the green region of the visible spectrum (∼550 nm) (Figure 1D). Under UV excitation at 365 nm, this shift is observable with the naked eye. Digital analysis techniques were used to quantify visual color changes under UV light. Images were split into individual color channels using ImageJ software, and the resulting values varied linearly as a function of recycled content. An example of this process for L*a*b*, RGB (red, green, blue), and HSV (hue, saturation, value) color spaces is depicted in Figure 3.

This procedure not only adds a third methodology for quantification but it also extends the applicability of our fluorescent-marked PCR concept to facilities without access to specialized fluorescence equipment. Unknown mixed samples could be compared to known reference standards, like the function of a universal indicator in the use of pH paper. This would allow “real-time” quick determination of recyclate content or as a quality-control check for manufacturers. The importance of quick checks is critical as industry may rapidly switch between batches or sources of recycled content. The accuracy of this technique could be further improved by calibrating color values using LEDs or light sources with confirmed wavelengths and colors. Layering of these quantification processes is key to minimizing fraudulent marking of PCR content and ensuring reliability between sites and waste streams, with the methodology supporting checks across the supply chain and encouraging compliance through its simplicity.

### Versatility and Industrial Compatibility

Following method development, the real-life applicability of our approach was evaluated by testing the effects of sample size, processing conditions, HDPE recyclate composition, and additive incorporation. Mechanical recycling processes rely on heat and shear forces and remain unstandardized across the plastics industry.^5^ To simulate variation in recycling processes, the HDPE MB (0.1 wt % MB) was subjected to injection molding across a range of temperatures (180–260 °C) and associated viscoelastic response (Figure S7). The resulting intensity ratios were found to be independent of temperature and remained constant at ≈ 0.8 for the 470 nm/430 nm ratio and ≈0.5 for the 500/430 nm ratio (Figure S7). These results indicate that at all relevant BBS concentrations (≤0.1 wt %), processing conditions are likely to not have any effect on the fluorescence measurements, suggesting applicability to the range of practices used in recycling and packaging industries.

To validate that intensity measurements were independent of sample size, fluorescence emission measurements were performed on compression molded films (120–880 μm thickness) as opposed to injection molded dumbbells (Figure S8). The intensity measurements and ratios were independent of sample thickness or size. Having such sample versatility enables use of fluorescence measurements for recycled content determination in a wide range of manufactured plastic products (Figures S8 and S9).

To further validate our approach, tests were performed on an industrially produced recycled HDPE stream sourced from recycled milk bottles (Figure S10). Emission profiles and intensity ratios were analogous to those performed with the simulated recycled content (Figures S10 and S11). Additionally, recycling simulations were performed on additive-altered 0.1 wt % BBS-HDPE MB. In two separate studies, the effect of adding 1 wt % UV stabilizer (Irganox 1010) and 0.005 wt % optical brightener (1,4-Bis(benzo[*d*]oxazol-2-yl)naphthalene, BBON) on recycled content marking was found to be minimal (Figure S13 and Tables S2 and S3). These tests evidence that this technique is independent of sample size and processing conditions, remains valid for industrially produced recyclate, and can withstand the presence of common plastic additive formulations. BBS is FDA-approved at the lower studied MB concentration of 0.025 wt % (Figures S4 and S5) for use in contact with aqueous, acidic, and basic media.^16,17^ Seven day leaching tests confirmed that no leaching of BBS from the polymer matrix can be seen in the relevant solvents approved by the FDA (Figure S20).

HDPE is one of several polymers that dominate the plastic packaging industry. Accordingly, we extended our initial research on HDPE recycled content determination to include PP and PET. A loading study of BBS dispersed in PP revealed that BBS displays aggregation behavior similar to that in HDPE (Figure S14). This agreement is unsurprising because of the similar polarity and high crystallinity values of both polymers, given their influence on BBS aggregate formation. Recycling simulation tests (10–100%) were then performed using a BBS-PP 0.1 wt % MB. A linear correlation was observed between recycled content and fluorescence emission ratios for PP at this BBS concentration, with intensity ratios comparable to those of HDPE under the same conditions (Figure S15). Both short- and long-lived fluorescence lifetimes (τ_1_ and τ_2_, respectively) increased upon aggregation and subsequent bathochromic shifts of BBS in PP (Figure S22).^45^ Optical color values also changed predictably (Figure S50).

Contrary to HDPE and PP, the aggregation threshold for BBS in an amorphous grade of PET was found to be significantly higher in analogous loading studies (Figure S16). We attribute this to two key differences: (1) the increased polarity and aromaticity of the PET chains disrupting the energetics of dimerization and (2) a lower crystallinity increasing the volume of the amorphous phase in which BBS segregates.

Consequently, a relatively higher master-batch concentration of 0.5 wt % was trialed for PET recycling simulations. Fluorescence emission measurements initially revealed no change in the intensity of the peaks at 470 and 500 nm with increasing recycled content (10–100%). No relationship was found between recycled content and emission ratios at both 470/430 nm and 500/430 nm for the prepared samples (Figure S18), corroborating low levels of BBS aggregation. For accurate quantification, we found that PET samples must be annealed (>1 h at 120 °C) to promote aggregation (Figure S17) by increasing crystallinity from ∼7 to ∼40%, thus increasing the concentration of BBS in the amorphous phase of the samples. Post-anneal, a linear relationship between intensity ratios and recycled content was observed (Figure S19), akin to those recorded for HDPE and PP. A similar disparity in trends between annealed and non-annealed samples was observed in the corresponding fluorescence lifetime data and optical color values (Figures S23 and S24 and S51), allowing for a coterminous solution to process history changes.

**Figure 4:**
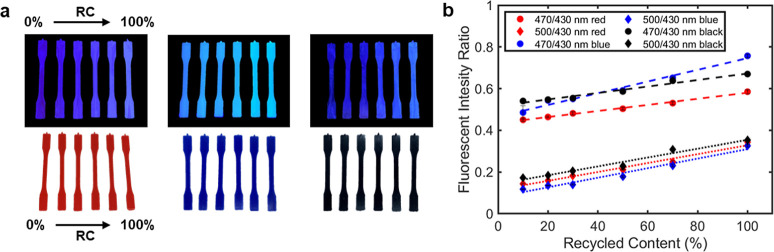
Recycled content (RC) determination in colored plastics. (A) Colored 0.1 wt % BBS-HDPE MB samples, top: illuminated at 365 nm and photographed in a blacked-out room, and bottom: under ambient lighting. (B) Resulting intensity ratios for red, blue, and black diluted 0.1 wt % BBS-HDPE samples between 470 (●), 500 (⧫), and 430 nm. Error bars represent the standard error (*n* = 5) where each sample comes from the same batch. Fits were produced using the MATLAB curve fitting toolbox [470/430 nm *R*^2^ = 0.9928, 0.9786, 0.9735 (red, blue, black) and 500/430 nm *R*^2^ = 0.9773, 0.9659, 0.9716 (red, blue, black)].

Colored plastics are also ubiquitous in packaging applications. Colored plastics, specifically those that use black pigments, notoriously obfuscate plastic waste sorting due to interference with spectroscopic detection techniques.^46^ To ensure that use of industrially relevant polymer colorants would not negatively affect the proposed marking technique, this research was extended to include common-color plastics. Concentrated colors (red, blue, and black) in pellet form were added to a 0.1 wt % BBS-HDPE MB at 1 wt % loading to produce fluorescent colored MBs; these were then diluted down in recycling simulations. BBS was undetectable to the naked eye in the colored samples, yet samples fluoresced brightly under UV illumination (Figure 4A). The overall fluorescence intensities of the red and black samples were found to be slightly diminished compared to the colorless BBS-HDPE samples. Despite this, intensity ratios between the 470, 500, and 430 nm peaks revealed linear relationships between intensity and recycled content for all three colors tested, including black (Figure 4B). Visual color analysis under UV irradiation also retained linear relationships for all three of the colors tested (Figure S54). These results further highlight the robustness of this fluorescence-based methodology and suggest the potential to additionally recognize and sort black plastic packaging in material recycling facilities.

Overall, the three plastics with which we have demonstrated successful implementation of our fluorescent marking approach—HDPE, PP, and PET—make up approximately 40% of total EU plastic demand (19.4% PP, 12.4% HDPE, and 7.9% PET),^47^ and indeed much higher percentages in single-use plastic packaging products. By expanding this research to cover the chemically similar LDPE (17.4% of EU plastic demand), this fluorescence-based methodology would be compatible with the majority of the plastic market.^46^ We also see no obvious reason why this approach would not be compatible with other highly used plastics. Maximizing coverage of this technology, both by plastic type and geographically, is key to improving global recycling rates.

### BBS-Polymer Characterization

**Figure 5:**
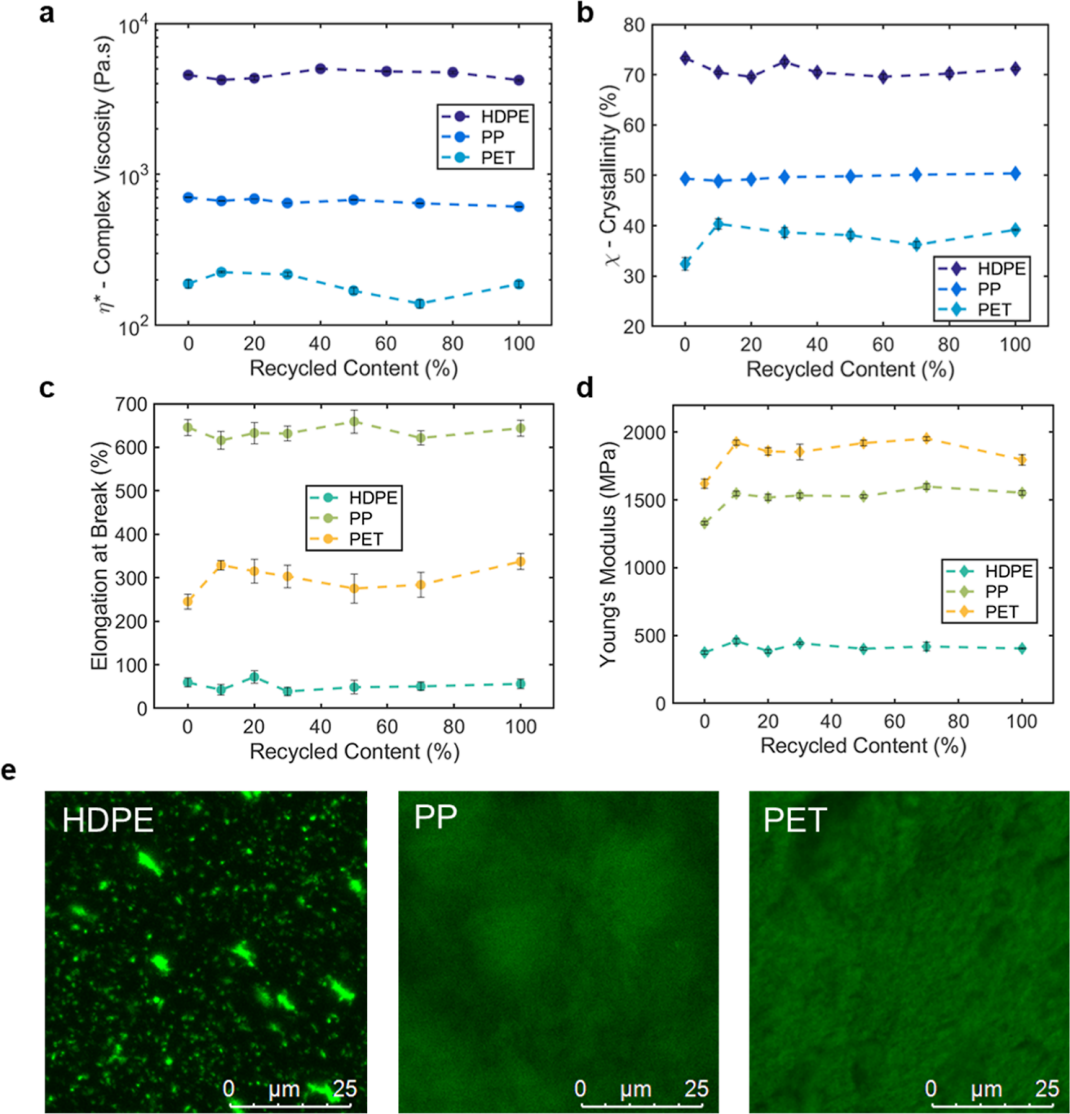
BBS-Polymer sample characterization of BBS MBs of HDPE (0.1 wt %), PP (0.1 wt %), and PET (0.5 wt %) using simulated recycled content. (A) Complex viscosity of diluted BBS MBs measured at 10 rad s^–1^, 1% strain, and 200 °C (HDPE and PP) or 280 °C (PET). (B) Crystallinity values of diluted BBS MBs measured using DSC (Supporting Information Section S5.3). (C) Elongation at break of diluted BBS MBs measured at 5 mm/min, 15 mm/min, and 40%/min, respectively. (D) Young’s moduli of diluted MBs determined from the elastic response of samples measured at 5 mm/min, 15 mm/min, and 40%/min, respectively. (E) Confocal images of BBS MB samples in the 530–620 nm range (excited at 405 nm) (see Figures S25–S29). Scale bar: 25 μm. Figure error bars represent the standard error [*n* = 3 for (A,B), *n* = 5 for (C,D)]. Fits were produced using the MATLAB curve fitting toolbox.

The relationship between aggregate sub-structures and BBS concentration was investigated using confocal microscopy. Aggregates were detected in all three polymer MBs: at 0.1 wt % BBS for HDPE and PP, and at 0.5 wt % for PET (Figures 5 and S25–S29). Many small, well-dispersed, and defined spherical aggregates and helical structures were detected in the 0.1 wt % BBS-HDPE samples (Figure S25). Images of PP and PET samples revealed a significantly different aggregate size and distribution. Very small BBS aggregates were well-dispersed throughout the PP sample and highlighted the spherulitic domains of PP (Figures 5E and S28). Infinitesimal aggregates of BBS were nonuniformly dispersed throughout PET in patchwork domains, potentially correlating with regions of lower crystallinity (Figures 5E and S29).

Recyclate use is economically disfavored because poor control of PCR feedstock, especially through unpredictable progressive incorporation of contaminants and additives, can diminish product performance.^5^ Furthermore, typical extrusion conditions promote thermo-mechanical and thermo-oxidative degradation through chain scission, cross-linking, and branching reactions. Widespread use of specialty additives has been shown to mitigate shear-induced structural modifications and deterioration in properties.^5^ If seeking to fraudulently mimic the presence of recyclate, controlled degradation through use of peroxides could be exploited to imitate the effects of chain scission. Indeed, rheological measurements can provide an estimate of MW (Cox-Merz Rule—see Materials and Methods) and have been proposed as a method for recyclate quantification.^48,49^ Within this complex relationship between additives and recyclate, it is important to ensure that BBS does not have unintended consequences on material quality.

Frequency sweeps of HDPE, PP, and PET MB recycling simulations showed no significant trend between recyclate fraction and complex viscosity, storage, or loss moduli (Figures 5A and S46–48). Similarly, no significant changes were seen in the thermal or tensile properties of the samples (Figure 5A–D and S33–S45). For example, crystallinity measurements obtained *via* DSC remained relatively consistent at ca. 70% (HDPE), 50% (PP), and 40% (PET) with increasing simulated recycled content (and thus BBS incorporation) (Figure 5B). Combined stability of complex viscosity, thermal properties, mechanical properties, and crystallinity confirms that BBS has negligible effect on extrusion-triggered chemical reactions. This is attributed to the thermal stability of BBS (*T*_d5%_ = 367.60 °C), which is not prone to radical formation and hydrogen abstraction along the polymer chain (Figure S52). The barrier properties of BBS-marked PP were also tested and showed a minor improvement versus the unmarked control (Supporting Information Section S10). Additionally, FT-IR spectra of the MB recycling simulations of HDPE, PP, and PET remained unaffected by the presence of BBS; a crucial feature required for IR-associated plastic waste sorting (Figures S30–S32). These data strongly indicate that there are no deleterious effects of BBS incorporation on plastic properties or chemical fingerprinting. The compatibility of this technique with existing plastic recycling infrastructure, without modifications, offers an advantage over comparable bureaucracy-heavy verification schemes and would further minimize costs. As this method is unaffected by polymer additives and physico-chemical structure, there is a clear advantage over the use of rheological and thermal measurements in recyclate quantification, where the source of material and process history heavily influence measurements.

To prevent fraud, this fluorescence-based approach is best enabled by exploiting established batch tracking methodologies for material integrity. This could be delivered in conjunction with the aforementioned mass balance methods (where it would avoid intermediary audit steps to decrease costs and increase analytical rigor) or through the implementation of a “trust mark” system enabling a supply chain to be rewarded for ambitious recycling targets in addition to taxation reporting. For example, attempts to exploit the technology by fraudulently marking batches of virgin plastic with BBS are theoretically possible, but circumvented through audit trails, as undisclosed concentrations would present illogical results. Furthermore, the presence of false dyes would be detectable through fluorescence lifetime measurements (before and after annealing). Haphazard attempts at doping in BBS to match the said recyclate concentration would return atypical readings due to the sensitivity of the detection method. Purchasing tagged recyclate with intent to reverse engineer the formulation would also be time consuming, cost prohibitive, and risky to the point that using the technology as intended would provide the path of least resistance, whereby compliance is easier and less expensive than fraud.

This method’s simplicity also translates to a practical advantage over more elaborate approaches seen in digital marking systems (*e.g.*, digital watermarks). These digital marks, though theoretically more information-rich, require reapplication at each stage of the product lifecycle and are generally applicable to only a single component. The decreased production line speed and requirements to audit across supply chains create more obvious opportunities for fraudulent recycled content reporting. In this context, fluorescent-based PCR marking with BBS fills a clear technological niche supporting the plastic recycling industry.

A straightforward PPT auditing process underpinned by BBS-labeling is envisioned through the help of transparent relationships across the polymer industry. Through collaboration with recyclers, rigorously audited PCR-BBS master-batches with pre-defined, undisclosed concentrations are produced. These master-batches would be diluted with virgin feedstock to pre-set recycled content values, before being processed into new plastic products and certified *via* a second audit stage. Considering the feedstock variability, throughput, and pace of the plastic recycling sector, the simplicity of this approach is fundamental to its application and offers a clear advantage over more time-consuming or process-altering methods.

### Conclusions and Future Prospects

We present a unique, process-independent method to mark recycled content in HDPE, PP, and PET that may represent a milestone in global efforts to improve mechanical recycling rates within the plastics industry. These polymers alone account for 40% of plastics use within the EU. This multi-pronged approach (fluorescence intensity, lifetime, and color) avoids disrupting existing processes and would enable quantification of subsequent individual plastic components.^50^ BBS is cheap, readily available, and has already received FDA and REACH approval for use in most food contact applications over the concentration ranges described herein. Furthermore, its incorporation has negligible impact on material and sorting properties, and the resulting quantification does not suffer from the common weaknesses observed in other PCR determinations, where PCR auditing is impossible due to uncontrolled, unstandardized additive incorporation, and variable processing conditions and recyclate quality. It is well-established that BBS is compatible with existing manufacturing processes, while this research confirms that a high quality recyclate can be maintained, thereby underpinning the first technique for recyclate quantification that is consistently accurate and broad in scope. The innovation interweaves three layers of detection, making it more difficult to falsify PCR content, while not inflating costs and being broadly applicable to products of various polymers, geometries, and colors.

The UK-based legislations introduced in April 2022 are already setting a legal precedent for the incorporation of recyclate globally. BBS-based recyclate marking has the potential to curb land-filling rates as an accessible and standardized international certification of recycled content. This innovation might best be enabled through batch-to-batch tracking of recycled content through to a product or component, with an associated certification scheme built on transparent partnerships to increase consumer trust in plastic recycling. Furthermore, the technology presented may also be applicable to a wide range of concentration-based fluorescent markers detailed by other research groups, such as mechanochromic dyes.^20,31−33,51^ This discovery is therefore a key tool toward rebuilding confidence in a circular plastics economy, addressing the plastic pollution problem through innovation, collaboration, and transparency.

## Materials and Methods

### Materials

HDPE was provided by and manufactured by Sabic. Sabic HDPE B624LS is a food-grade polymer pellet of MFI of 0.5 dg/min at 190 °C/2.16 kg. Its quoted melting point is 135 °C. rHDPE, stemming from a recycled milk bottle waste stream, was provided by Alpla. 4,4′-Bis(2-benzoxazolyl) stilbene (BBS), also known as Fluorescent Brightener 393 or Rylux OB-1, was supplied by Tokyo Chemical Industry UK and used as received. PET was provided by Unilever and purchased from Hardie Polymers. Food-grade virgin Ramapet N1 polymer pellets, manufactured by Indorama Ventures, with a quoted melting point of 247 ± 2 °C and of extrusion grade {[η] = 0.80 ± 2 dL/g} were used as the virgin PET. PP was purchased from Hardie Polymers. PP Capilene T 89 E was manufactured by Carmel Olefins Ltd with a MFI of 25 g/10 min at 230 °C/2.16 kg and a quoted vicat softening temperature of 153 °C. 1,4-Bis(benzo[*d*]oxazol-2-yl)naphthalene (BBON), trade name Hostalux KCB, was supplied by Fluorochem and used as received. Irganox 1010 was provided by BASF and used as received. Quinine sulfate was purchased from Sigma-Aldrich and used as received.

### Sample Preparation

PET pellets were dried in a Fistreem vacuum oven fitted with an Edwards RV5 vacuum pump at 120 °C for 16 h to prevent hydrolytic scission during processing; HDPE and PP pellets were not dried. Polymer-dye blends were prepared by compounding 4 g of polymer with 0.005–1.675 wt % (with respect to polymer matrix) of BBS in a HAAKE Minilab II micro twin-screw compounder at 200 °C (HDPE and PP) or 280 °C (PET) with a screw speed of 100 rpm (HDPE and PP) or 30 rpm (PET). The dyed samples were immediately quenched in a room-temperature water-bath and pelletized (2.5 mm) using a HAAKE Process 16 Varicut Pelletizer. Samples were subject to a drying step before further processing to remove residual water from the quenching step. The master-batches were compounded with virgin polymer pellets either in a HAAKE Minilab II, a HAAKE Polylab 16, or a HAAKE Process 11 to produce samples with simulated recycled contents varying from 10 to 100% (maintaining processing at 200 °C and 100 rpm for PP and HDPE, or 280 °C for PET).

### Dumbbell Preparation

Polymer-BBS pellets (0.005–1.675 wt %) were individually injection molded into dumbbells to match ISO 527-2-1BA using a HAAKE Minijet II micro piston injection molder. Injection molding was completed with a cylinder temperature of 200 °C (HDPE and PP) or 280 °C (PET) and a mold temperature of 60 °C (HDPE and PP) or 80 °C (PET), an injection pressure of 600 bar for 5 s, and a post-injection pressure of 300 bar for 5 s.

### Film Preparation

Samples of the same recyclate/virgin ratios were compression molded using a Collin Platen Press P300 P/M. HDPE- and PP-based samples were pressed at 200 °C into 880 μm/120 μm width films between aluminum foil and film molds for 3 min at 20 bar. PET-based samples were pressed at 280 °C into 880 μm/120 μm width films between Teflon films and film molds for 1 min at 20 bar. Samples were slowly cooled to 40 °C in the hot press, removed, and left to cool at room temperature.

### Blown Film Sample Preparation

Blown films were prepared by extruding virgin PP and 0.1 wt % BBS-PP MB through a HAAKE Polylab 16 at 220 °C and 100 rpm fitted with a HAAKE melt pump and 35 mm blown film die both held at 220 °C and a melt pump speed of 18 rpm. Films were air cooled and wound using a HAAKE blown film tower.

### Additive-Altered Sample Preparation

A BBON-HDPE master-batch (2.5 wt % relative to polymer matrix) was prepared using a HAAKE Minilab II micro twin-screw compounder at 200 °C and 100 rpm. The master-batch was diluted to 0.1 wt % in a HAAKE Polylab 16 at 200 °C and 100 rpm and subsequently blended with the 0.1 wt % BBS-HDPE recycling simulations using identical conditions. Irganox 1010 was blended into the 0.1 wt % BBS—HDPE MB (1 wt % relative to polymer matrix) in a HAAKE Polylab 16 at 200 °C and 100 rpm. All additive-altered samples were injection molded into a HAAKE Minijet II micro piston injection molder using previously stated conditions.

### Fluorescence Emission Spectra

Fluorescence intensity measurements were conducted at room temperature on a Cary Eclipse Fluorescence Spectrophotometer from Agilent paired with Cary Eclipse Software. Emission spectra were obtained by exciting dumbbell samples at 325 nm using a slit width of 2.5 mm for outgoing and incoming beams and measuring emission from 350 to 600 nm. The slit width was increased to 5 mm for emission measurements of black samples. The resulting spectra were normalized with respect to the isolated BBS molecule peak at ∼430 nm. The ratio of the excimer bands (∼500 and ∼470 nm) to isolated band (∼430 nm) was then determined and used as an indicator of aggregation and subsequent recycled content. Five samples of each sample batch were produced, and each measured once unless specified otherwise. Errors were calculated by dividing standard deviation by the square root of sample number.

### Fluorescence Quantum Yields

Fluorescence intensity measurements were conducted at room temperature on a Cary Eclipse Fluorescence Spectrophotometer from Agilent paired with Cary Eclipse Software. Absorbance measurements were obtained on a Perkin Elmer LAMBDA 365 + UV/Vis Spectrometer. The fluorescence quantum yields (Φ) of the PP—BBS recycling simulation samples were calculated using quinine sulfate in a 0.5 M H_2_SO_4_ solution and eq 2 (Table S4).
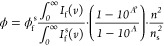
2where Φ_f_ represents the quantum yield of the quinine standard, ∫_0_^*∞*^*I*_f_(*v*) and ∫_0_^*∞*^*I*_f_^s^(*v*) represent the area beneath the sample and standard emission spectra, respectively (λ_excite_ = 310 nm), *A* and *A*^s^ represent the absorbances of the samples and standard, respectively at λ_excite_ = 310 nm, and *n* and *n*_s_ represent the refractive indices of the samples and standard, respectively.^24,52^ The quantum yield of quinine sulfate in 0.5 M H_2_SO_4_ is quoted as 0.545 and a refractive index of 1.346. The refractive index of PP was taken as 1.49.^24,52^

### Fluorescence Lifetimes

Fluorescence lifetime experiments were performed on an Edinburgh Instruments F900 paired with F900 software. Excitations were performed using a 340 nm picosecond pulsed LED with a 500 ns pulse period. Emission lifetimes were measured over 200 ns, de-convoluted, and the resulting decay fitted to a multi-exponential decay function using non-linear least-square fitting *via* the F900 software (eq 3 and Supporting Information Section S2.1.1). Five samples of each sample batch were produced, and each measured once unless specified otherwise. Errors were calculated by dividing standard deviation by the square root of sample number.
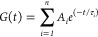
3where *A*_*i*_ represents decay amplitude, and τ_*i*_ represents the lifetime parameter and time, *t*.

### Digital Photographs and Optical Analysis

Digital photographs of polymer-BBS samples were taken using an IPhone XS camera under an analytikjena 6 W UV excitation lamp on long wavelength (365 nm) in a UVP Chromato viewing cabinet. ImageJ software was used to separate the resulting photos into RGB, L*a*b*, and HSB stacks. The corresponding value for each channel was taken as the average over a pre-determined area and plotted against the recycled content. Errors were taken as the standard deviation from taking the average over the pre-determined area.

### Confocal Microscopy

Confocal microscopy pictures were taken using a Leica SP8 confocal microscope with a pulsed 405 nm laser for excitation. Film samples were mounted on glass slides and covered with glass slip covers. The films were imaged using a 63 × 1.20 numerical aperture oil immersion objective. The laser scan speed was set to 100 Hz, and the pinhole aperture was set to 1.0 Airy. Image sizes were set to 1024 × 1024 pixels. Multiple images were taken across the entire sample.

### Mechanical and Thermal Properties

#### Rheological Measurements

Frequency sweeps were conducted on a DH-R2 Rheometer using an environmental test chamber and a stainless steel 25 mm parallel plate geometry under a nitrogen flow. Sweeps were performed at 200 °C (HDPE and PP) or 280 °C (PET) from 0.1 to 600 rad s^–1^ at a strain of 0.3% (HDPE and PP) or 1% (PET) and collecting 10 points per decade. The measurement gap was set to 1000 mm, and samples were trimmed at 1050 mm before starting tests. The complex viscosity of the samples was recorded and relates to the steady-state viscosity by the Cox-Merz rule.^48,49^
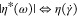
4

When
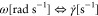
5

And  is defined as
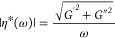
6where *G*^′^ and *G*^″^ are the storage and loss moduli, respectively, and  is the complex modulus.^48,49^ A total of three runs were produced per sample batch. Errors were calculated by dividing standard deviation by the square root of sample number.

#### Tensile Testing

Tensile tests were conducted on a Static Testing Instron 3344L3928 fitted with either a 500 N or 2000 N load cell. Samples, matching ISO 527-2-1BA, were tested at a constant speed of either 5 mm/min (HDPE) or 15 mm/min (PP), or a constant strain of 40%/min (PET). The strain at break ε_b_, strain at yield ε_y_, stress at yield σ_y_, stress at break σ_b_, and Young’s Modulus E were measured and calculated. A minimum of five samples were tested per sample batch accordant with ISO 527-2-1BA. Errors were calculated by dividing standard deviation by the square root of sample number.

#### Differential Scanning Calorimetry

All DSC experiments were performed on a TA instruments DSC 2500. All samples were prepared to weigh between 3 and 10 mg and were placed in *T*_zero_ pans and fitted with *T*_hermetic_ lids. Samples were subject to an initial equilibration step at 40 °C before a thermal ramp at 10 °C•min^–1^ up to 250 °C (HDPE and PP) or 300 °C (PET) to erase the thermal history and then cooled to −80 °C at 5 °C•min^–1^ and finally heated at 10 °C•min^–1^ to 250 °C (HDPE and PP) or 300 °C (PET). Nitrogen was used as a purge gas at 50 mL•min^–1^. Three repeat runs were performed per sample unless stated otherwise. Analysis of runs was performed on the TA control software. Crystallinity was calculated according to eq 7, where Δ*H*_m_ and Δ*H*_c_ are the melting and cold-crystallization enthalpies, respectively, and Δ*H*_m_^°^ is the melting enthalpy of perfectly crystalline HDPE (293 J/g), PP (207 J/g), and PET (140 J/g). DSC measurements were conducted on three different samples from the same batch. Errors were calculated by dividing standard deviation by the square root of sample number.
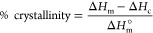
7

#### Barrier Properties

Water vapor transmission rate (WVTR) measurements were performed on an Extra Solution PermeH_2_O DC Water Vapor Analyzer. The WVTR of blown films of virgin PP and the BBS-PP 0.1 wt % MB of 40 μm thickness was tested at 37.8 °C at 90% relative humidity with medium conditioning settings {>10 [g/(m^2^•24 h)]}.^53^ Measurements were repeated twice for confirmation.

#### Leaching Experiments

0.4 g of 2.5 mm pellets of 0.1 wt % BBS-HDPE MB (relative to polymer matrix) were submerged in 1 mL of EtOH, IPA, DI water, Olive Oil, and CHCl_3_ at room temperature for 7 days. Fluorescence emission spectra were recorded on the 7th day of testing, and measurements were conducted at room temperature on a Cary Eclipse Fluorescence Spectrophotometer from Agilent paired with Cary Eclipse Software. Emission spectra were obtained by exciting samples in a 1 mL quartz cuvette at 325 nm using a slit width of 2.5 mm for outgoing and incoming beams and measuring emission from 350 to 600 nm.

#### Fourier-Transform Infra-red Spectroscopy

FT-IR spectra were recorded on a Bruker INVENIO. 64 scans were performed between 400 and 4000 cm^–1^, and peaks were manually picked on the INVENIO software.

#### Thermogravimetric Analysis

Thermogravimetric analysis was performed on a TA Simultaneous Thermal Analyzer SDT 650. Two runs were performed on each sample.

## Data and Materials Availability

Data available on request.

## Supporting Information Available

Additional raw data from fluorescence (emission, lifetimes, and confocal microscopy), characterization techniques (FT-IR, rheology, *etc.*), and further experimental details (PDF)
