# Supplementary material for: Recycled Plastic Content Quantified through Aggregation-Induced Emission
Source: ACS Sustain Chem Eng. 2022 Sep 13;10(38):12659–69. doi: 10.1021/acssuschemeng.2c03389 (PMC9516760; doi:10.1021/acssuschemeng.2c03389)
Supplement: Supplementary file 1 — sc2c03389_si_001.pdf [file sc2c03389_si_001.pdf]

Supplementary information

Recycled plastic content quantified through aggregation-induced emission

Zoé O. G. Schyns, Thomas M. Bennett and Michael P. Shaver*

Pages - 60

Figures - 54

Tables - 5

Table of Contents

Table of Contents S2
1 Fluorescence Emission S4
1.1 HDPE Fluorescence Emission Spectra....S4
1.1.1 Variable Dye Loadings....S4
1.1.2 Lower concentration master-batches....S5
1.1.3 Scaling up of 0.1 wt% Master-batch S7
1.1.4 Process Independent Fluorescence Emission Spectra S9
1.1.5 Sample Size Independent Fluorescence Emission Spectra S10
1.1.6 Proof of concept using rHDPE....S11
1.1.7 Deconvolution of 0.1 wt% Spectra....S13
1.1.8 Additive-Altered 0.1 wt% Recycling Simulations....S14
1.2 PP Fluorescence Emission Spectra....S16
1.2.1 Variable Dye Loadings....S16
1.2.2 PP 0.1 wt% Recycling Simulation....S17
1.2.3 Quantum Yield of PP 0.1 wt%....S17
1.3 PET Fluorescence Emission Spectra....S19
1.3.1 Variable Dye Loadings....S19
1.3.2 PET 0.5 wt% Recycling Simulation....S21
1.4 Linear Fits for Fluorescence Emission spectra S22
1.5 Fluorescence Leaching Studies....S23
2 Fluorescence Lifetimes S24
2.1.1 Fitting Equation for Fluorescence Lifetimes....S24
2.1.2 HDPE 0.1 wt% master-batch S25
2.1.3 PP 0.1 wt% master-batch S26
2.1.4 PET 0.5 wt% master-batch S28
3 Confocal Microscopy....S31
3.1 HDPE S31
3.2 PP S34
3.3 PET S35
4 FT-IR....S36
4.1 HDPE S36
4.2 PP S37
4.3 PET S38
5 DSC....S39
5.1 Exemplary DSC Curve S39

5.2 Thermal Properties S40
5.2.1 HDPE S40
5.2.2 PP S41
5.2.3 PET S41
5.3 Crystallinity S42
5.3.1 HDPE S43
5.3.2 PET S43
5.3.3 PP S44
6 Mechanical Properties S45
6.1 Elongation at Break S45
6.1.1 HDPE S45
6.1.2 PP S46
6.1.3 PET S46
6.2 Young's Modulus S47
6.2.1 HDPE S47
6.2.2 PP S47
6.2.3 PET S48
7 Rheological Properties S49
7.1 HDPE S49
7.2 PET S50
7.3 PP S51
8 Colour Analysis S52
8.1 HDPE S52
8.2 PP S54
8.3 PET S55
9 TGA of 4,4′-bis(2-benzoxazolyl)stilbene S56
10 Water Vapour Transmission Rates S57
11 Recycling Simulations of Coloured HDPE Samples S58
11.1 Fluorescence Emission S58
11.2 Optical analysis S59
12 References S60

Results

1 Fluorescence Emission

1.1 HDPE Fluorescence Emission Spectra

1.1.1 Variable Dye Loadings

Initially it was deemed important to probe the range of concentrations at which BBS molecules would display dimeric fluorescence responses. BBS was directly dispersed into the HDPE polymer matrix through melt blending at a large range of concentrations (0 – 1.675 wt%). Evidence of dimer formation were readily detectable at dye loadings as low as 0.025 wt%. Following this, fluorescent MBs were prepared at 0.1 wt%.

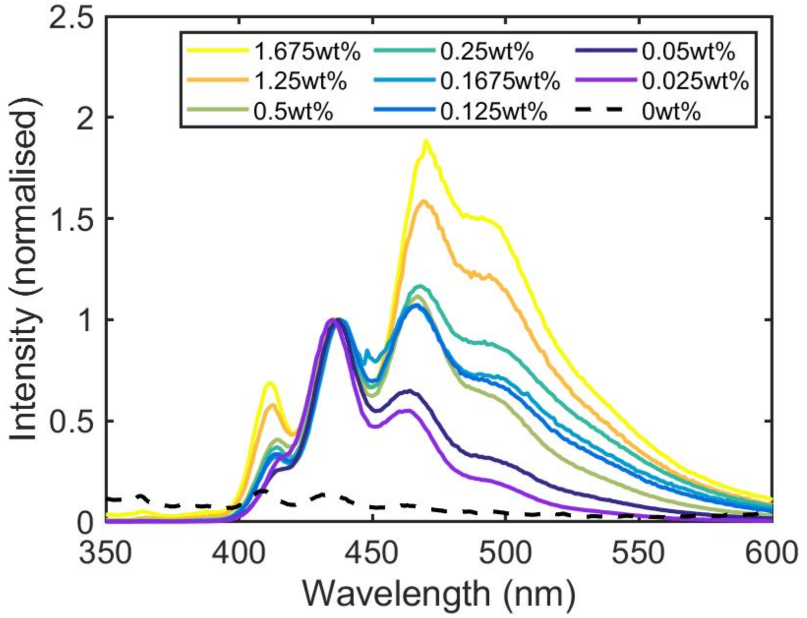

Figure S1 - Fluorescence emission spectra of varying BBS loading in HDPE (0.025 – 1.675 wt%). Emission spectra normalised at the fluorescence emission maximum of isolated molecules, corresponding to the 0 → 1 transition.

1.1.2 Lower concentration master-batches

The following tests were produced using a small-scale compounder with a 7 ml mixing section. During tests it was noted that for these small-scale experiments, fluorescence data deviated more when compared to their scaled-up equivalents.

1.1.2.1 0.05 wt%

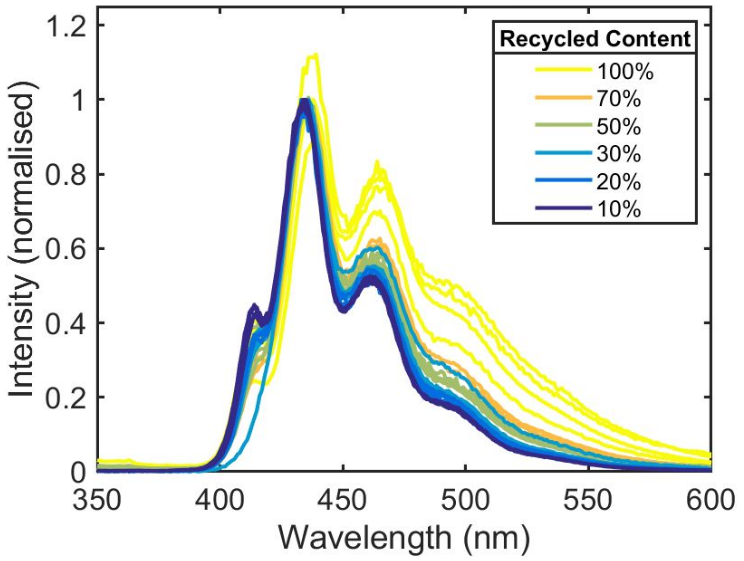

Figure S2 - Fluorescence emission spectra of diluted 0.05 wt% BBS-HDPE MB. Emission spectra normalised at the fluorescence emission maximum of isolated molecules, corresponding to the 0 → 1 transition.

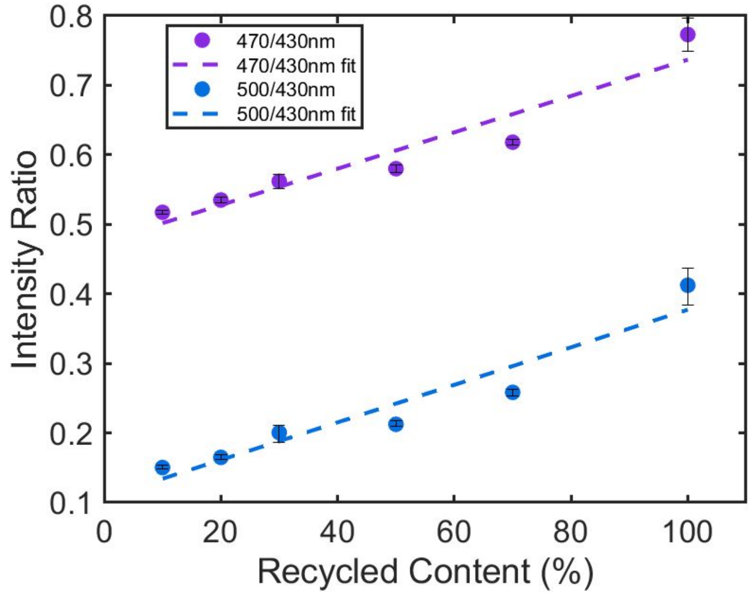

Figure S3 - Fitted intensity ratios for diluted BBS-HDPE 0.05 wt% MB. Resulting intensity ratios between 470, 500 and 430 nm for 0.05 wt% MB. Error bars represent the standard error (n = 5). Fits produced using the MATLAB curve fitting toolbox. R^2 values tabulated in *SI 1.4*.

1.1.2.2 0.025 wt%

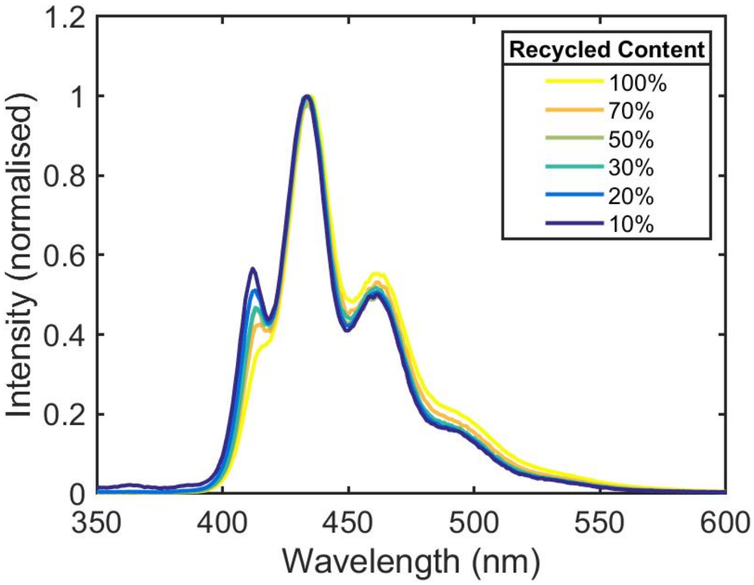

Figure S4 - Fluorescence emission spectra of diluted 0.025 wt% BBS-HDPE MB. Emission spectra normalised at the fluorescence emission maximum of isolated molecules, corresponding to the 0 → 1 transition.

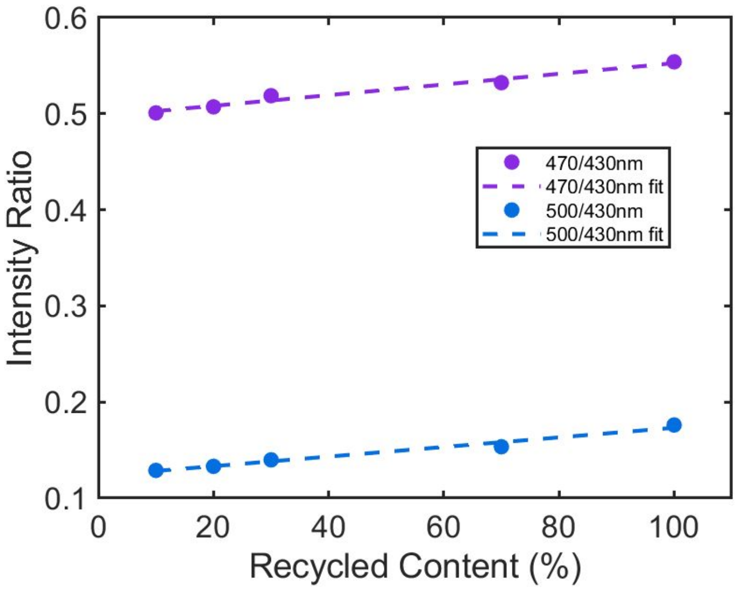

Figure S5 - Fitted intensity ratios for diluted BBS-HDPE 0.025 wt% MB. Resulting intensity ratios between 470, 500 and 430 nm for 0.025 wt% MB. Fits produced using the MATLAB curve fitting toolbox. R^2 values tabulated in SI 1.4.

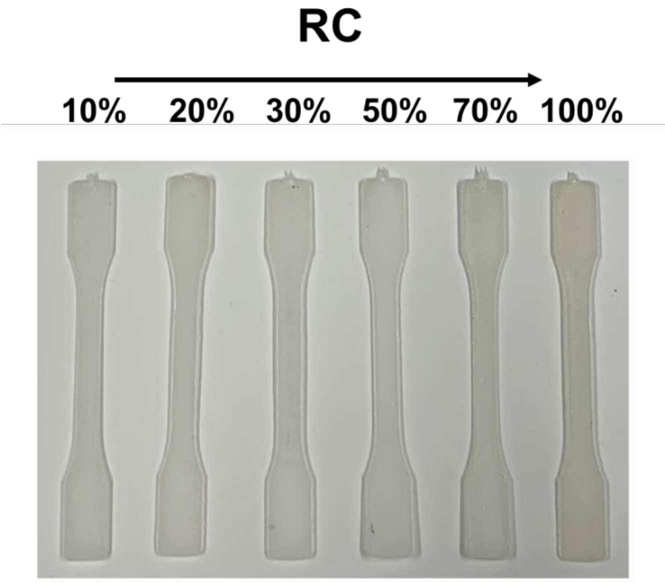

Figure S6 – Photograph of recycling simulation of 0.025 wt% BBS-HDPE MB samples. Samples photographed under ambient lighting.

Only the 0.1 wt% HDPE MB was scaled up from the 4 g to 80 g scale. The 0.1 wt% concentration was chosen to maintain both the linearity of the fluorescence ratio with increasing recycled content and to maintain compliance with FDA and REACH standards for food contact.

1.1.3 Scaling up of 0.1 wt% Master-batch

40 g of concentrated BBS-HDPE MB was created by melt blending BBS and HDPE at 2.5 wt% in a HAAKE Minilab II micro twin-screw compounder at 200 °C with a screw speed of 100 rpm. The dyed samples were immediately quenched in a room-temperature water-bath and pelletised (2.5 mm) using a HAAKE Process 16 Varicut Pelletizer. The MB was then diluted to 0.1 wt% by melt blending the 2.5 wt% MB with virgin HDPE in a HAAKE Polylab. The dyed samples were immediately quenched in a room-temperature water-bath and pelletised (2.5 mm) using a HAAKE Process 16 Varicut Pelletizer. The MB was then compounded with virgin polymer pellets in a HAAKE Polylab to produce roughly 80 g of samples with simulated recycled contents varying from 10-100 % (maintaining processing at 200 °C and 100 rpm). The polymer-BBS pellets were individually injection moulded into dumbbells to match ISO

527-2-1BA using a HAAKE Minijet II micro piston injection moulder. Injection moulding was completed with a cylinder temperature of 200 °C and mould temperatures of 60 °C, injection pressure of 600 bar for 5 s and a post-injection pressure of 300 bar for 5 s.

The same generic process was used to produce scaled-up PP and PET masterbatches. Processing conditions for PP were the same as those for HDPE: 200 °C with a screw speed of 100 rpm with identical injection moulding conditions, cylinder temperature of 200 °C and mould temperatures of 60 °C, injection pressure of 600 bar for 5 s and a post-injection pressure of 300 bar for 5 s. For PET an extrusion temperature of 280 °C and screw speed of 100 rpm was used, and injection moulding conditions of 280 °C cylinder temperature, 80 °C mould temperature, injection pressure of 600 bar for 5 s and a post-injection pressure of 300 bar for 5 s.

Table S1 – Tabulated BBS concentration relative to recycled content for recycling simulations of the 0.1 wt% MB of HDPE and PP.

Recycled Content (%)	Approximate BBS concentration (wt%)
10	0.01
20	0.02
30	0.03
40	0.04
60	0.06
80	0.08
100	0.10

1.1.4 Process Independent Fluorescence Emission Spectra

To ensure that BBS recyclate marking was truly process independent, the 0.1 wt% BBS-HDPE MB was injection moulded at several temperatures between 180-260 °C. It was found that the final fluorescence intensity ratio was unchanged with processing temperature.

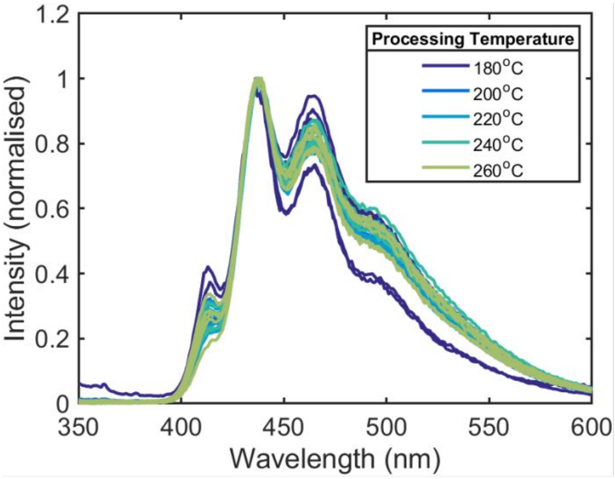

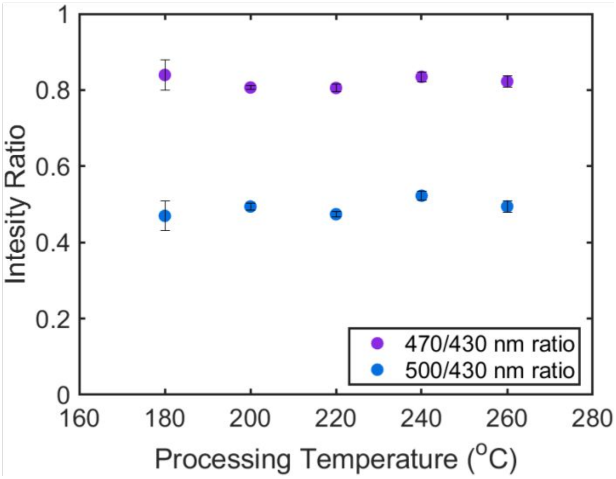

Figure S7 - Left: Fluorescence emission spectra of 0.1 wt% BBS-HDPE MB injection moulded at different temperatures. All spectra normalised at 430 nm. 5 samples tested per temperature. Right: Fluorescence intensity ratios of both 470/430 nm and 500/430 nm for the 0.1 wt% MB samples injection moulded at different temperatures. Error bars represent the standard error (n = 5) where each sample comes from the same batch.

1.1.5 Sample Size Independent Fluorescence Emission Spectra

The following tests were produced using a small-scale compounder with a 7 ml mixing section. During these tests it was noted that fluorescence data for the small-scale experiments deviated more when compared to the scaled-up experiments.

Fluorescence emission tests were performed on injection moulded dumbbells. The 0.1 wt% BBS-HDPE MB was pressed into films to confirm that the fluorescence intensity ratios were independent of sample thickness/size.

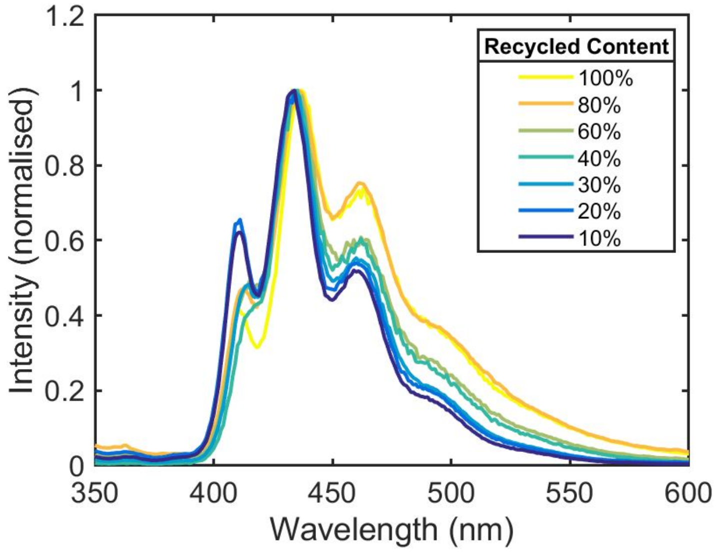

Figure S8 - Fluorescence emission spectra of diluted 0.1 wt% BBS-HDPE MB pressed into films. Emission spectra normalised at the fluorescence emission maximum of isolated molecules, corresponding to the 0 → 1 transition.

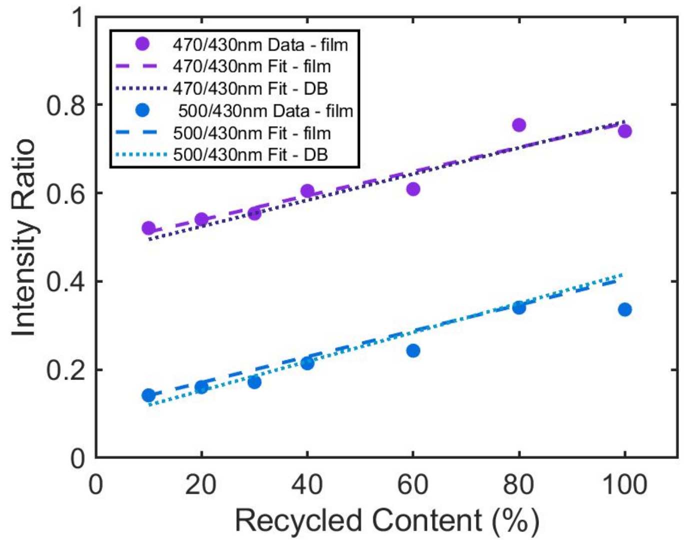

Figure S9 - Fitted intensity ratios for diluted BBS-HDPE 0.1 wt% MB pressed into films. Resulting intensity ratios between 470, 500 and 430 nm for 0.1 wt% MB films (dashed line) compared to dumbbells (dotted line). Fits produced using the MATLAB curve fitting toolbox. R^2 values tabulated in SI 1.4.

1.1.6 Proof of concept using rHDPE

To evaluate the real-life applicability of this emission-based verification of recycled content, the recycling simulation was performed on a genuine recycled HDPE stream sourced from recycled milk bottles. Emission profiles and fluorescence intensity ratios were analogous to those performed with the simulated recycled content, thus confirming the industrial applicability of this research.

The following test was produced using a small-scale compounder with a 7 ml mixing section. During tests it was noted that on the small-scale experiments, fluorescence data deviated more when compared to the scaled-up experiments.

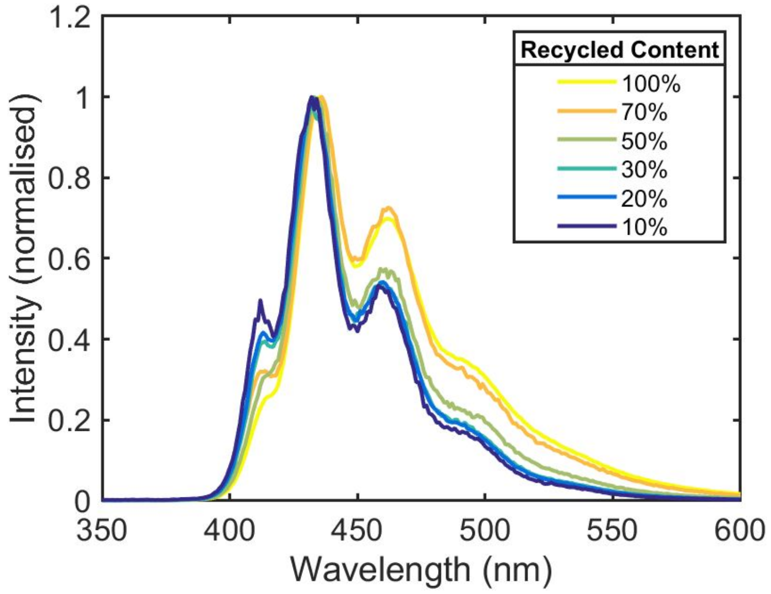

Figure S10 - Fluorescence emission spectra of diluted 0.1 wt% BBS-rHDPE MB from a milk bottle waste stream. Emission spectra normalised at the fluorescence emission maximum of isolated molecules, corresponding to the 0 → 1 transition.

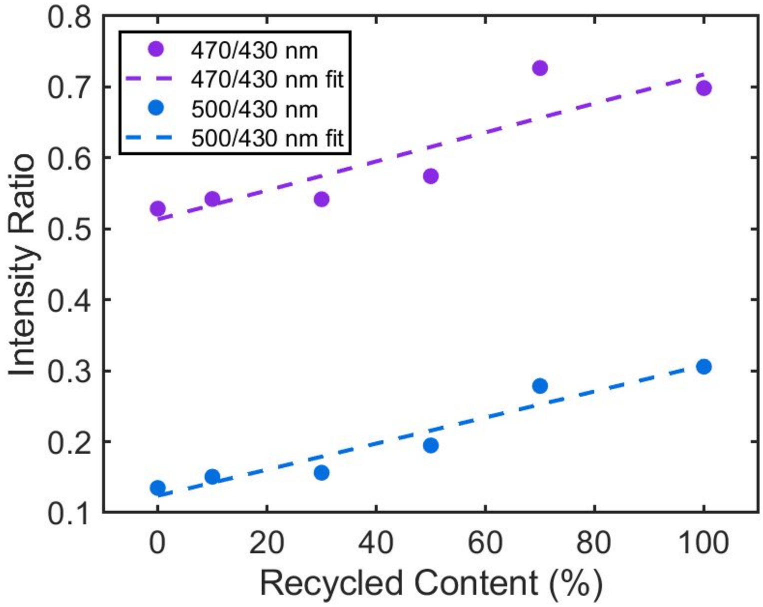

Figure S11 - Fitted intensity ratios for diluted BBS-HDPE 0.1 wt% MB from milk bottle recyclate stream. Resulting intensity ratios between 470, 500 and 430 nm for 0.1 wt% MB samples. Fits produced using the MATLAB curve fitting toolbox. R^2 values tabulated in SI 1.4.

1.1.7 Deconvolution of 0.1 wt% Spectra

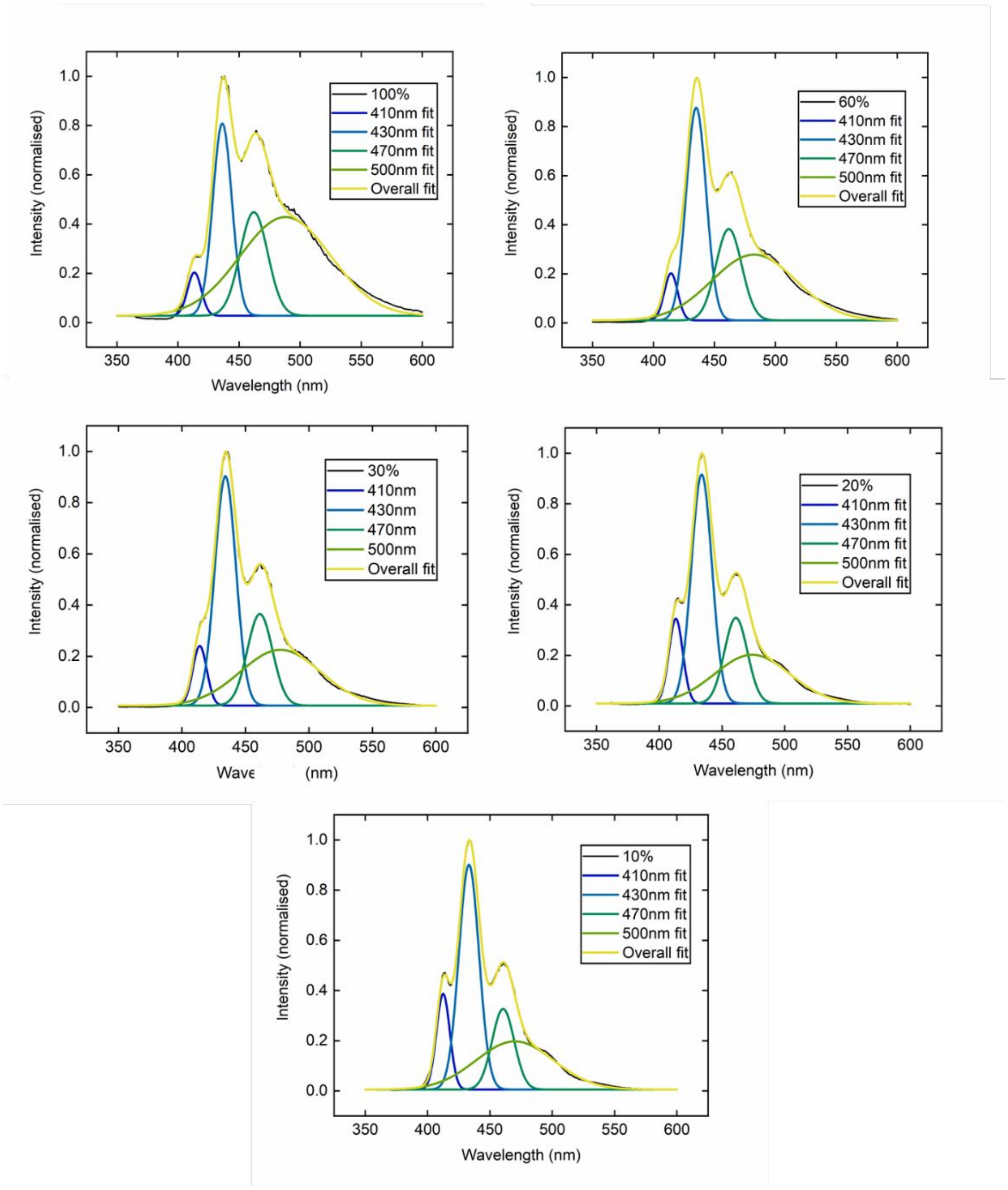

Figure S12 – Deconvolution of the fluorescence emission spectra of the diluted 0.1 wt% BBS-HDPE MB. Deconvoluted by fitting with four Gaussians and produced using Origin's multi-peak fitting system.

To investigate the nature of the fluorescence emission response, the fluorescence emission peaks were deconvoluted by fitting 4 gaussian peaks to the emission data using Origin software. From this, it was seen that the largest contributor to both the increase in the peak at 470 and 500 nm was due to dimer fluorescence peak formation. The intensity of the 500 nm dimer peak increases with increasing recycled content and occurrence of dimerisation.

1.1.8 Additive-Altered 0.1 wt% Recycling Simulations

The effect of including typical plastic additives, such as UV stabilizers or optical brighteners, on the fluorescence response of the BBS based marking system was investigated.

1.1.8.1 Hostalux KCB

The optical brightener 1,4-Bis(benzo[d]oxazol-2-yl)naphthalene (BBON), trade name Hostalux KCB, a typically used in many polymers and with an absorption wavelength similar to BBS, was blended into the 0.1 wt% BBS-HDPE recycling simulation at a typical OB loading of 0.005 wt%.[1] The fluorescence emission spectra and corresponding intensity ratios were unchanged from those of the BBS-HDPE reference recycling simulation.

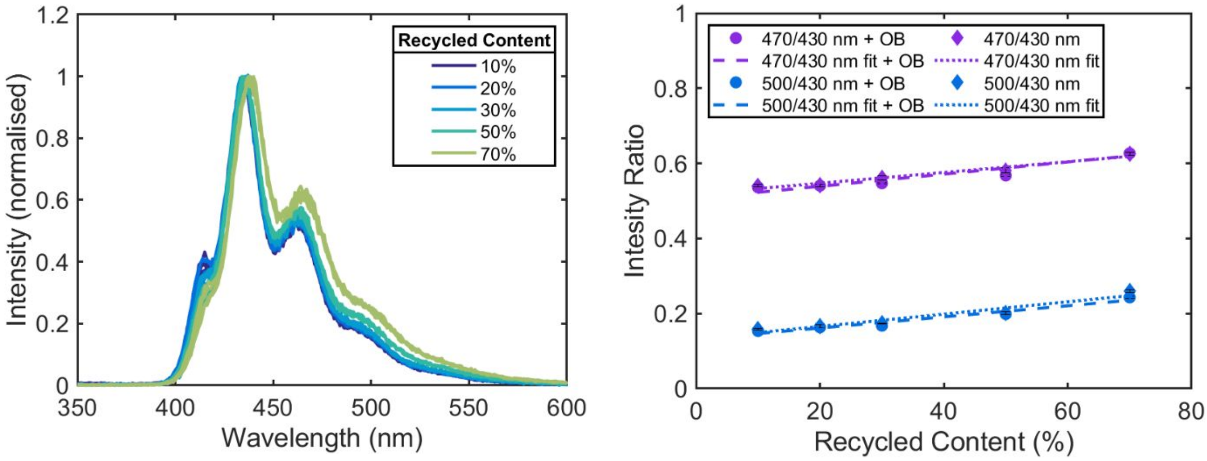

Figure S13 - Fluorescence emission spectra of varying recycled content for HDPE marked by BBS (0.1 wt%) and blended with optical brightener Hostalux KCB, normalised to 1 at the fluorescence emission maximum of isolated molecules. RHS: Resulting intensity ratios between 470, 500 and 430 nm for HDPE recycled simulations (470/430 nm R^2 = 0.9542 and 500/430 nm R^2 = 0.9749).

1.1.8.2 Irganox 1010

No discernable difference in the fluorescence emission ratios between the standard BBS-HDPE 0.1 wt% MB and its analogue modified with 1 wt% Irganox® 1010.

Table S2 – Intensity ratios for the BBS-HDPE 0.1 wt% MB and for the 1 wt% Irganox® 1010 modified 0.1 wt% BBS-PP calculated from emission spectra.

Sample	Ratio 470:430	Ratio 500:430
BBS-PP	0.709 ± 0.004	0.437 ± 0.007
IRGANOX-BBS-PP	0.696 ± 0.004	0.400 ± 0.005

Table S3 – Tabulated short- (τ_1) and long-lived (τ_2) fluorescence lifetime parameters at 470nm and 500nm for the 0.1 wt% BBS-HDPE MB and the Irganox® 1010 modified 0.1 wt% BBS-PP MB.

	Lifetime parameters 470nm		Lifetime parameters 500nm	
**Sample**	τ_1	τ_2	τ_1	τ_2
BBS-PP	1.34	13.42	1.51	16.71
IRGANOX-BBS-PP	1.22	12.34	1.35	15.03

1.2 PP Fluorescence Emission Spectra

1.2.1 Variable Dye Loadings

BBS was directly dispersed into the PP polymer matrix through melt blending over a large range of concentrations (0-1.675 wt%) as was previously completed for HDPE (*SI 1.1.1.*). Evidence of dimer formation was detectable at dye loadings as low as 0.025 wt%. Following this, fluorescent MBs were prepared at 0.1 wt%.

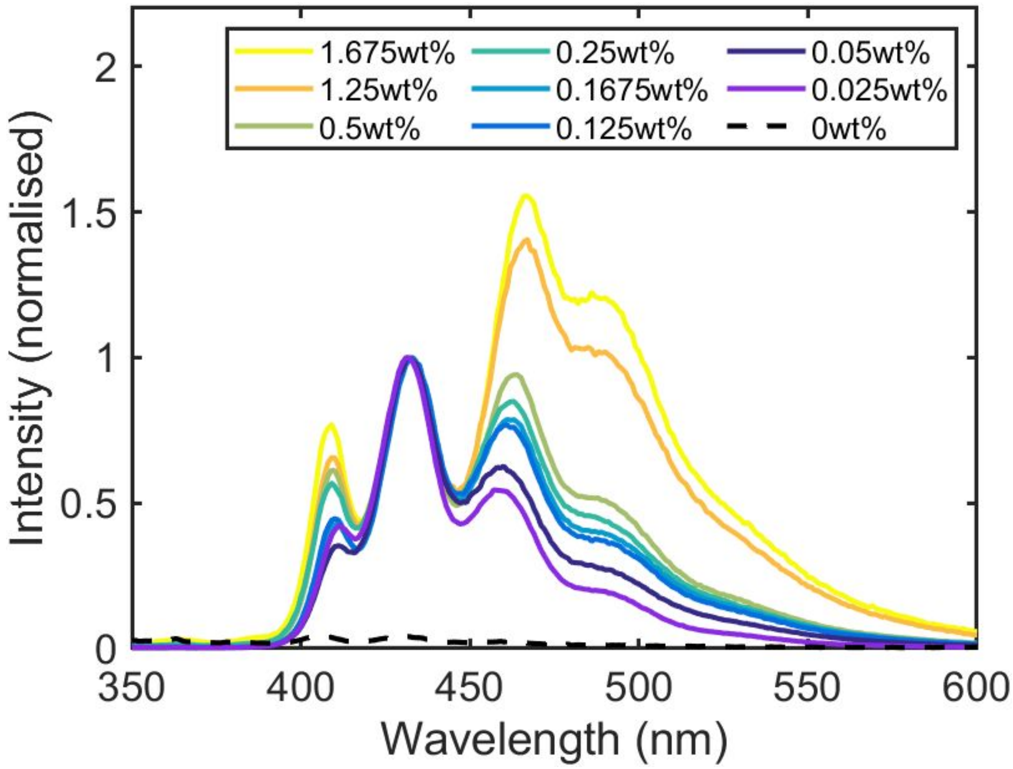

Figure S14 - Fluorescence emission spectra of varying BBS loading in PP (0.025-1.675 wt%). Emission spectra normalised at the fluorescence emission maximum of isolated molecules, corresponding to the 0 → 1 transition.

1.2.2 PP 0.1 wt% Recycling Simulation

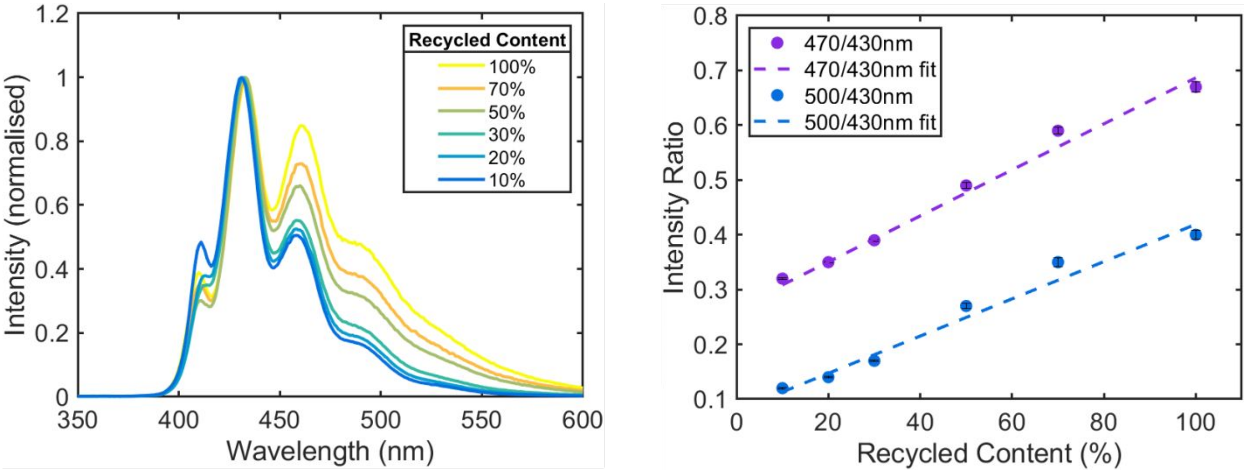

Figure S15 – LHS: Fluorescence emission spectra of varying recycled content for PP recyclate marked by BBS, normalised to 1 at the fluorescence emission maximum of isolated molecules. RHS: Resulting intensity ratios between 470, 500 and 430 nm for PP recycled simulations (470/430 nm R^2 = 0.9957 and 500/430 nm R^2 = 0.992).

1.2.3 Quantum Yield of PP 0.1 wt%

Quantum yields of BBS-PP (0.1 wt%) were measured relative to quinine 0.007 mg/ml quinine sulfate in 0.5 M H_2SO_4 according to equation 1.

$$\phi = \phi_f^s \frac{\int_0^\infty I_f(v)}{\int_0^\infty I_f^s(v)} \cdot \left(\frac{1-10^{A^s}}{1-10^{A}}\right) \cdot \frac{n^2}{n_s^2} \#(1)$$

Where Φ_f represents the quantum yield of the quinine standard, $\int_0^\infty I_f(v)$ and $\int_0^\infty I_f^s(v)$ represent the area beneath the sample and standard emission spectra respectively (λ_{excite} = 310 nm), A and A^s represent the absorbances of the samples and standard respectively at λ_{excite} = 310 nm, n and n_s represent the refractive indices of the samples and standard respectively.[2,3] The quantum yield of quinine sulfate in 0.5 M H_2SO_4 is quoted as 0.545 and a refractive index of 1.346. The refractive index of PP was taken as 1.49.[2,3]

Table S4 – Quantum yield values of 0.1 wt% BBS-PP blown film relative to a 0.007 mg/ml quinine sulfate in 0.5 M H_2SO_4 standard.

	Quantum Yield Φ
Quinine Sulfate Reference	0.545
BBS-PP Blown Film (0.1 wt%)	0.233

1.3 PET Fluorescence Emission Spectra

1.3.1 Variable Dye Loadings

As completed for HDPE and PP, BBS was directly dispersed into the PET polymer matrix through melt blending at a range of concentrations (0-1.675 wt%). Evidence of dimer formation was detectable at dye concentrations of 0.05 wt% and above but only weak differences in the emission spectra were recorded due to the polarity and low crystallinity of the PET chains.

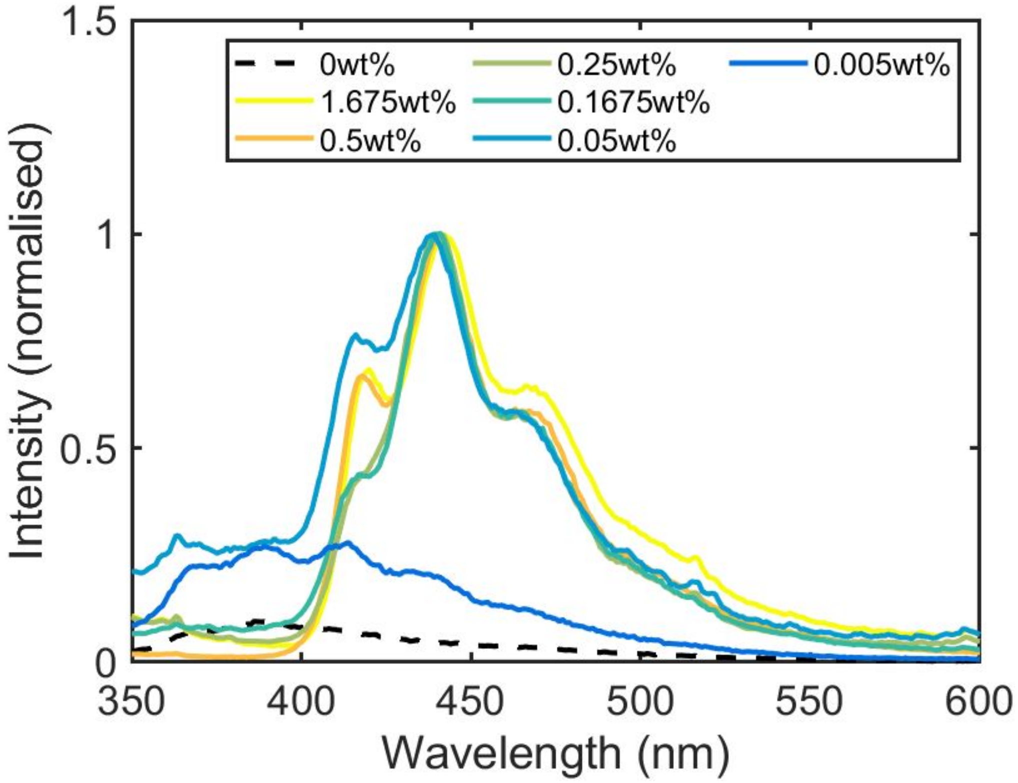

Figure S16 - Fluorescence emission spectra of varying BBS loading in PET (0.025-1.675 wt%). Emission spectra normalised at the fluorescence emission maximum of isolated molecules, corresponding to the 0 → 1 transition.

To increase aggregation levels, PET samples were annealed in a vacuum oven at 120 °C for 5 hours under vacuum. Following this annealing step, aggregation of the BBS in PET and subsequent increases in the fluorescence at 470 nm and 500 nm was more pronounced. This increase in fluorescence was most noticeable for the 1.675 wt% concentration sample where fluorescence intensity increased by almost 3-fold at 500 nm.

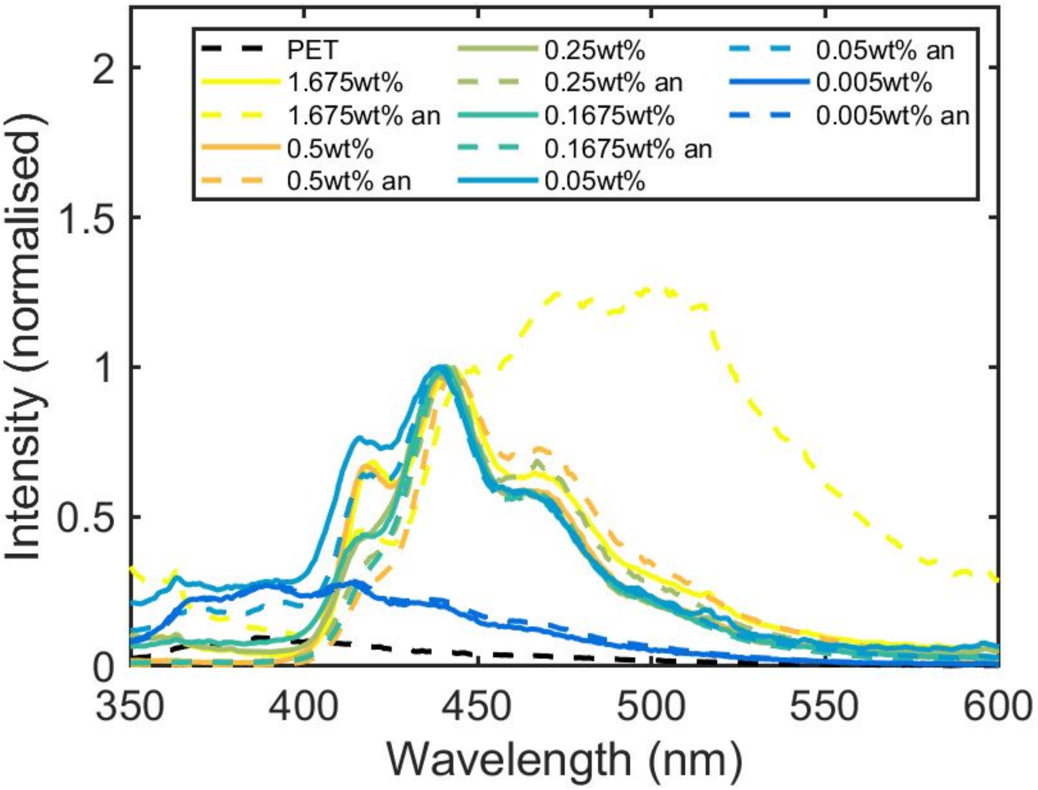

Figure S17 - Fluorescence emission spectra of varying BBS loading in HDPE (0.025-1.675 wt%). Emission spectra normalised at the fluorescence emission maximum of isolated molecules, corresponding to the 0 → 1 transition.

1.3.2 PET 0.5 wt% Recycling Simulation

1.3.2.1 Unannealed

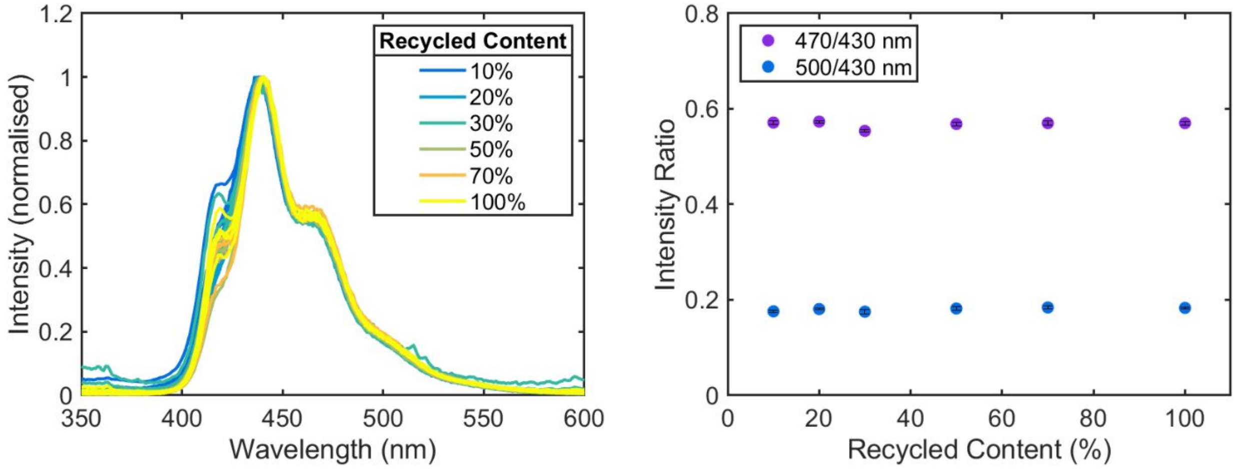

Figure S18 - LHS: Fluorescence emission spectra of varying recycled content for PET recyclate marked by BBS (0.5 wt%), normalised to 1 at the fluorescence emission maximum of isolated molecules. RHS: Resulting intensity ratios between 470, 500 and 430 nm for PET recycled simulations.

1.3.2.2 Annealed

PET samples were annealed at 120 °C for 5 hours to increase aggregation levels.

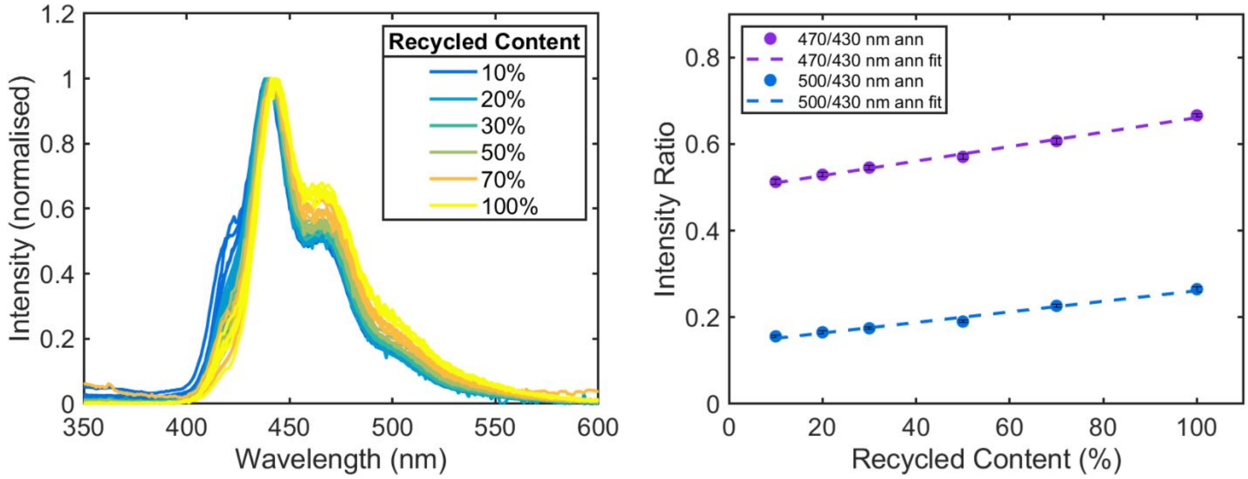

Figure S19 - LHS: Fluorescence emission spectra of varying recycled content for annealed PET recyclate marked by BBS (0.5 wt%), normalised to 1 at the fluorescence emission maximum of isolated molecules. RHS: Resulting intensity ratios between 470, 500 and 430 nm for PET recycled simulations (470/430 nm R^2 = 0.9834 and 500/430 nm R^2 = 0.9937).

1.4 Linear Fits for Fluorescence Emission spectra

The ratio of dimer to monomer peak was calculated using:

$$I_{ratio} = \frac{I_{470nm\ or\ 500nm}}{I_{430nm}} \#(2)$$

This resulting intensity ratio was plotted against the recycled content of the samples. The linearity of the plots was only found to hold for the full recycled content range when the initial MB concentration was less than 0.2 wt% for HDPE or PP, and less than 0.5 wt% for PET. This loss of linearity at high concentrations was attributed to the well-known fluorescence phenomenon of aggregate induced quenching.

Table S5 - R^2 values for truncated linear fits to intensity ratios for 470/430 nm and 500/430 nm for MB concentrations of 0.5, 0.1, 0.05 and 0.025 wt%. Fits produced using the MATLAB curve fitting toolbox according to the specified fitting ranges. (*) 0.05 wt% and 0.025 wt% MBs were created in a 7 ml small scale compounder.

**Polymer sample**	**BBS concentration (wt%)**	**R^2 (470:430 nm)**	**R^2 (500:430 nm)**	**Gradient (470:430 nm)**	**Gradient (500:430 nm)**
HDPE	0.1	0.9681	0.9635	$2.97 \cdot 10^{-3}$	$3.30 \cdot 10^{-3}$
HDPE	0.05*	0.9078	0.9131	$2.61 \cdot 10^{-3}$	$2.70 \cdot 10^{-3}$
HDPE	0.025*	0.9752	0.9768	$5.55 \cdot 10^{-4}$	$4.99 \cdot 10^{-4}$
HDPE films	0.1	0.9095	0.9507	$2.74 \cdot 10^{-3}$	$2.42 \cdot 10^{-3}$
rHDPE	0.1	0.7806	0.9295	$2.05 \cdot 10^{-3}$	$1.84 \cdot 10^{-3}$
Black HDPE (1 wt%)	0.1	0.9735	0.9716	$1.56 \cdot 10^{-3}$	$2.12 \cdot 10^{-3}$
Red HDPE (1 wt%)	0.1	0.9928	0.9773	$1.46 \cdot 10^{-3}$	$2.14 \cdot 10^{-3}$
Blue HDPE (1 wt%)	0.1	0.9786	0.9659	$2.80 \cdot 10^{-3}$	$2.29 \cdot 10^{-3}$
PP	0.1	0.9957	0.992	$4.20 \cdot 10^{-3}$	$3.40 \cdot 10^{-3}$
PET (annealed)	0.5	0.9834	0.9937	$1.22 \cdot 10^{-3}$	$1.67 \cdot 10^{-3}$

1.5 Fluorescence Leaching Studies

Though BBS is FDA approved for use in food-contact materials, preliminary leaching studies were performed in several industry-relevant solvents. Samples were submerged for 7 days in relevant solvents. Subsequent fluorescence emission measurements were performed on solvents. No fluorescence response was detected for buffer solutions of pH 4, 9 or DI water which suggests no leaching of BBS from the polymer matrix. Fluorescence traces, typical of BBS, were detected in some solvents such as ethanol, propanol, acetone and olive oil, meaning that our system would have limited applicability for alcohol-related uses and high fat-content products. Current EU regulations state that plastic materials should not release more than 10 mg of substances per 1 dm^2 of surface area.[4] It is important to recognize that fluorescence intensity measurements are not quantitative and that an in-depth, gravimetric long term leaching test should be performed before dismissing applicability of BBS for alcohol oily-substance related use.

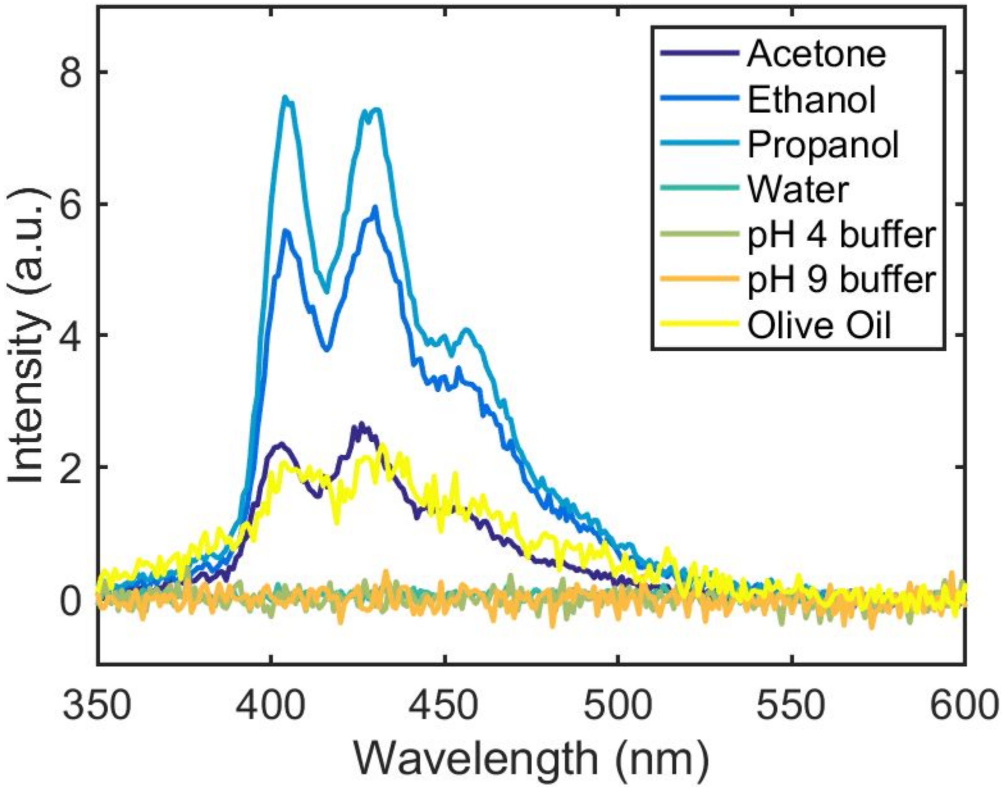

Figure S20 - Fluorescence emission spectra of solvents from seven day leaching tests of 0.1 wt% BBS-HDPE MB pellets in solvent (0.4 mg/ml).

2 Fluorescence Lifetimes

2.1.1 Fitting Equation for Fluorescence Lifetimes

The F900 software was used for all fluorescence lifetime fitting. Bi-exponential decay functions were found to be most suitable for the decay pattern shown by the BBS aggregates.

$$F(t) = \sum_i a_i e^{\frac{-t}{\tau_i}} \#(3)$$

Equation S1 – Multi-functional exponential decay fitting equation for fluorescence emission lifetime measurements. Where a_i represents decay amplitude, τ_i represents the lifetime parameter and time, t.

$$F(t) = A_1 e^{\frac{-t}{\tau_1}} + A_2 e^{\frac{-t}{\tau_2}} \#(4)$$

Equation S2 – Bi-functional exponential decay fit for fluorescence emission lifetime measurements. Where a_1 and b_1 represent decay amplitudes, τ_1 and τ_2 represent the short and long-lived lifetime parameters respectively and time, t.

2.1.2 HDPE 0.1 wt% master-batch

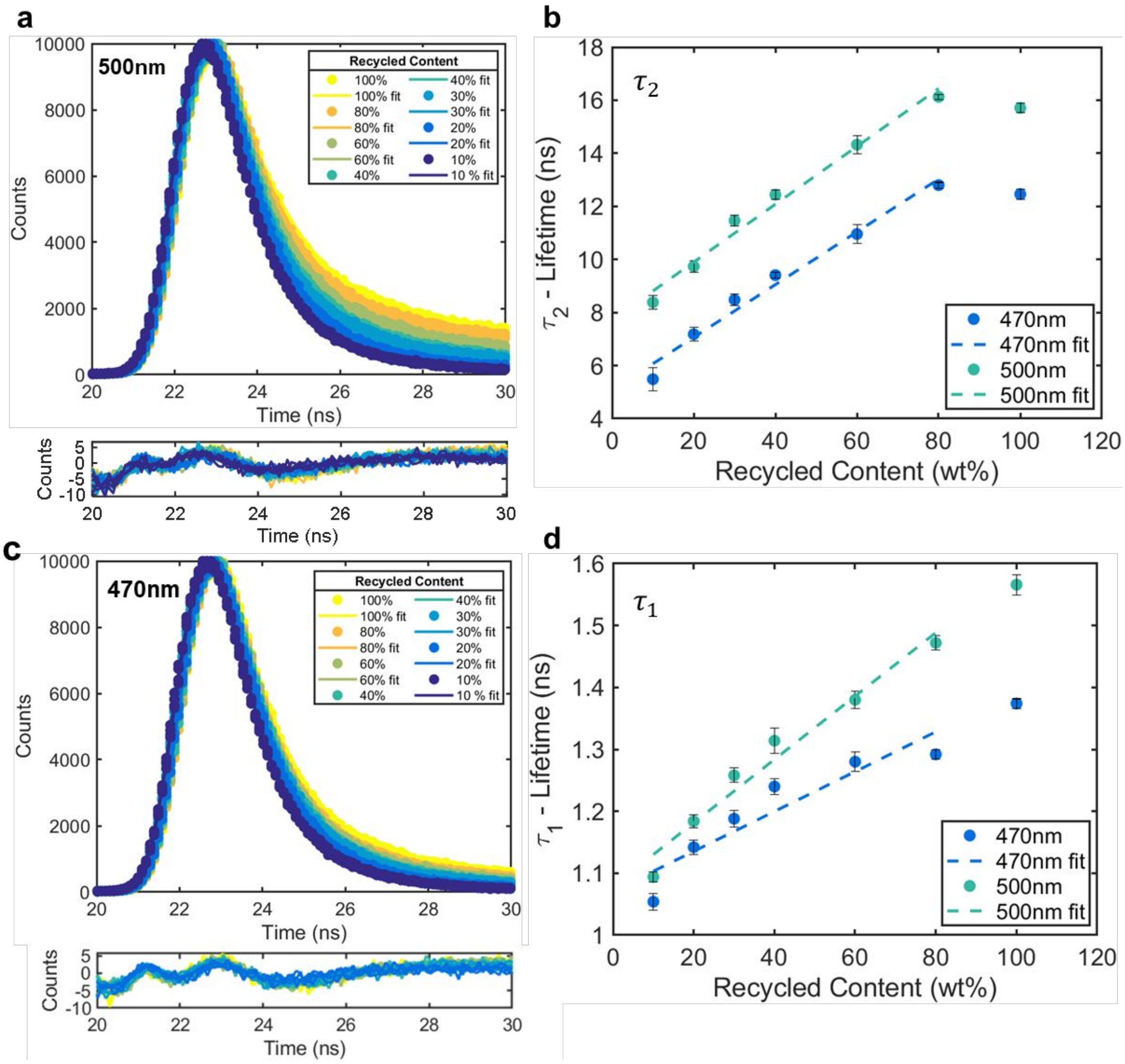

Figure S21 - Fluorescence lifetime measurements for diluted BBS-HDPE 0.1 wt% MB. (**A**) Fluorescence lifetime traces for diluted 0.1 wt% MB, excitation at 430 nm, measurement at 500 nm. (**B**) τ_2 – long lived lifetime parameter from bi-functional exponential function with increasing recycled content measured at 470 and 500 nm with excitation wavelength of 340 nm. Error bars represent the standard error (n = 5). Fits produced using the MATLAB curve fitting toolbox (470nm R^2 = 0.9791, 500nm R^2 = 0.9839). (**C**) Fluorescence lifetime traces for diluted 0.1 wt% MB, excitation at 430 nm, measurement at 470 nm. (**D**) τ_1 – short lived lifetime parameter from bi-functional exponential function with increasing recycled content measured at 470 and 500 nm with excitation wavelength of 340 nm. Error bars represent the standard error (n = 5).

2.1.3 PP 0.1 wt% master-batch

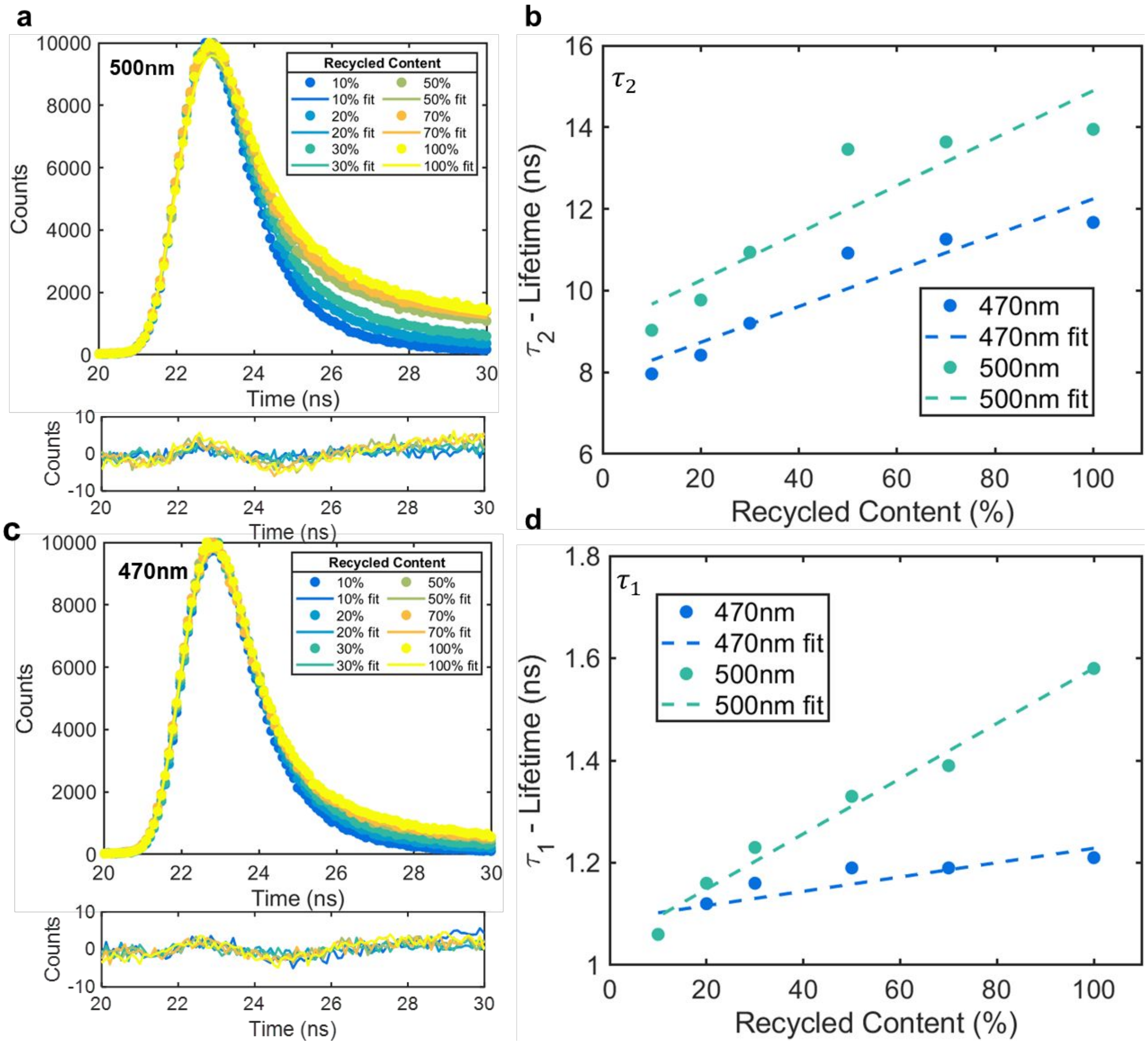

Figure S22 - Fluorescence lifetime measurements for diluted 0.1 wt% BBS-PP MB. (**A**) Fluorescence lifetime traces for diluted 0.1 wt% PP MB, excitation at 430 nm, measurement at 500 nm. (**B**) τ_2 – long lived lifetime parameter from bi-functional exponential function with increasing recycled content measured at 470 and 500 nm with excitation wavelength of 340 nm. Fits produced using the MATLAB curve fitting toolbox (470nm R^2 = 0.8871, 500nm R^2 = 0.831). (**C**) Fluorescence lifetime traces for diluted 0.1 wt% PP MB, excitation at 430 nm, measurement at 470 nm. (**D**) τ_2 – short lived lifetime parameter from bi-functional exponential function with increasing recycled content measured at 470 and 500 nm with excitation wavelength of 340 nm. Fits produced using the MATLAB curve fitting toolbox (470nm R^2 = 0.7446, 500nm R^2 = 0.9807).

At 470 nm, τ_2 decreased from ~12 ns at 100 % recycled content to ~8 ns at 10 % recycled content compared to ~12 ns at 100 % recycled content to ~5 ns at 10 % recycled content for HDPE (*Figure S22*). At 500 nm these lifetimes dropped from ~14 ns at 100 % recycled content to ~9 ns at 10 % recycled content compared to from ~16 ns at 100 % recycled content to ~8 ns at 10 % recycled content for HDPE (*Figure S22*). These differences in lifetime can be

recognised as effects arising from the difference in interactions between BBS and the host polymer matrix, due to differences in molecular structure between HDPE and PP.[5]

2.1.4 PET 0.5 wt% master-batch

2.1.4.1 Unannealed

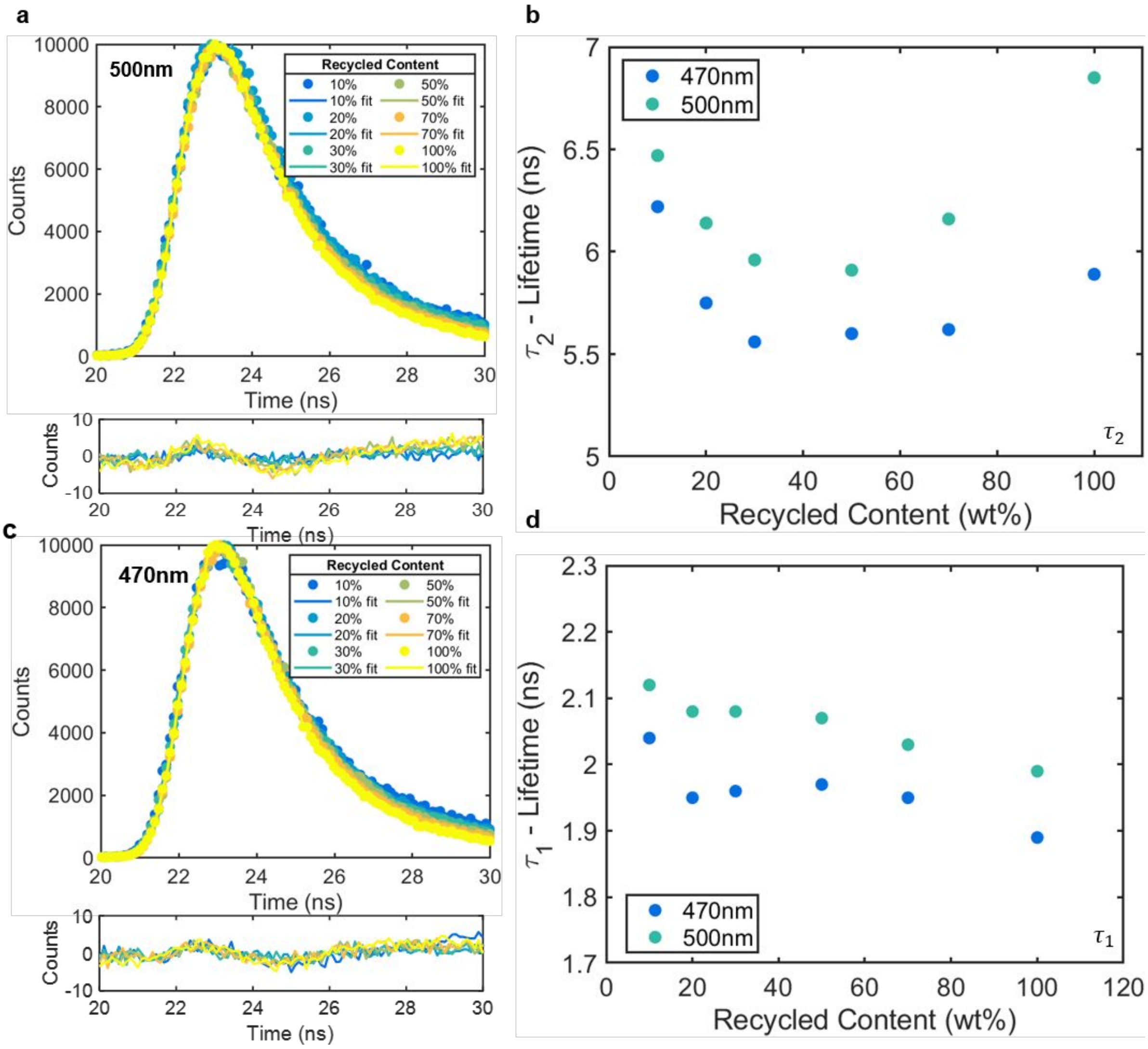

Figure S23 - Fluorescence lifetime measurements for diluted 0.5 wt% BBS-PET MB. (**A**) Fluorescence lifetime traces for diluted 0.5 wt% PET MB, excitation at 430 nm, measurement at 500 nm. (**B**) τ_2 – long lived lifetime parameter from bi-functional exponential function with increasing recycled content measured at 470 and 500 nm with excitation wavelength of 340 nm. (**C**) Fluorescence lifetime traces for diluted 0.5 wt% PET MB, excitation at 430 nm, measurement at 470 nm. (**D**) τ_1 – short lived lifetime parameter from bi-functional exponential function with increasing recycled content measured at 470 and 500 nm with excitation wavelength of 340 nm.

Annealed

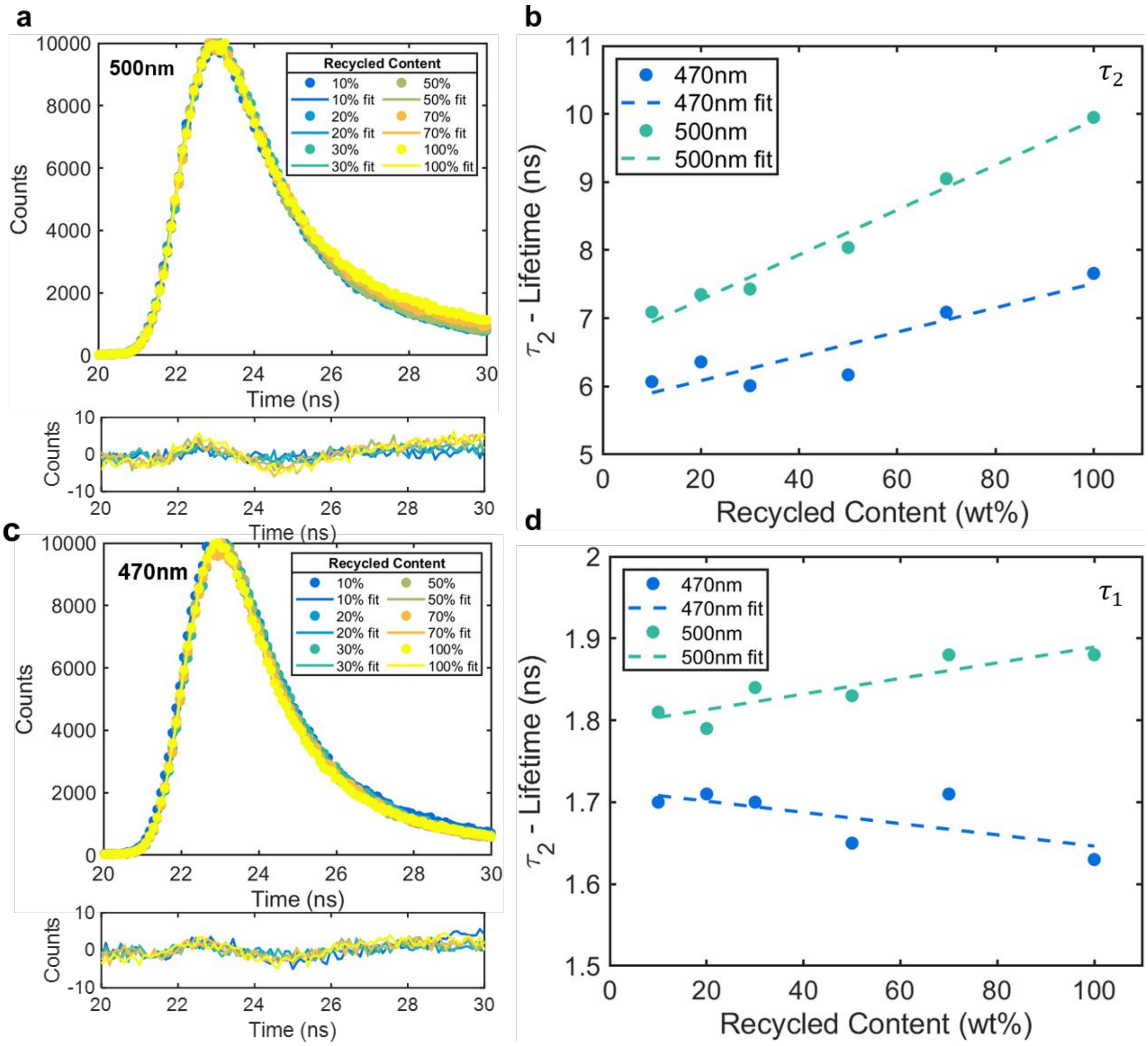

Figure S24 - Fluorescence lifetime measurements for diluted annealed 0.5 wt% BBS-PET MB. (**A**) Fluorescence lifetime traces for diluted annealed 0.5 wt% PET MB, excitation at 430 nm, measurement at 500 nm. (**B**) τ_2 – long lived lifetime parameter from bi-functional exponential function with increasing recycled content measured at 470 and 500 nm with excitation wavelength of 340 nm. (**C**) Fluorescence lifetime traces for diluted annealed 0.5 wt% PET MB, excitation at 430 nm, measurement at 470 nm. (**D**) τ_1 – short lived lifetime parameter from bi-functional exponential function with increasing recycled content measured at 470 and 500 nm with excitation wavelength of 340 nm. Fits produced using the MATLAB curve fitting toolbox (470 nm R^2 = 0.4548, 500 nm R^2 = 0.7799).

Similar to fluorescence emission measurements, trends in fluorescence lifetimes for the 0.5 wt% PET BBS MB were only observed in annealed samples. At 470 nm, τ_2 stayed constant for unannealed samples ~ 6 ns at 100 % recycled content to ~ 6 ns at 10 % recycled content compared to ~8 ns at 100 % recycled content to ~6 ns at 10 % recycled content for annealed samples (*Figure S23* and *Figure S24*). For the unannealed samples, τ_2 recorded at 500 nm remained constant at ~6 ns at both 100 % and 10 % recycled content. Upon annealing, τ_2 decreased from ~10 ns at 100 % recycled content to ~ 7 ns at 10 % recycled content for the annealed samples (*Figure S23* and *Figure S24*).

3 Confocal Microscopy

The relationship between aggregation sub-structures and concentration was investigated using confocal microscopy. Confocal images of samples pressed into films were taken between 530 and 620 nm to minimise detection of monomeric BBS (*Figure S25-S29*). Images were taken at regular intervals throughout the sample and processed into projections on the LASX software. Particle analysis was performed using ImageJ and the average aggregate sizes reported below.

3.1 HDPE

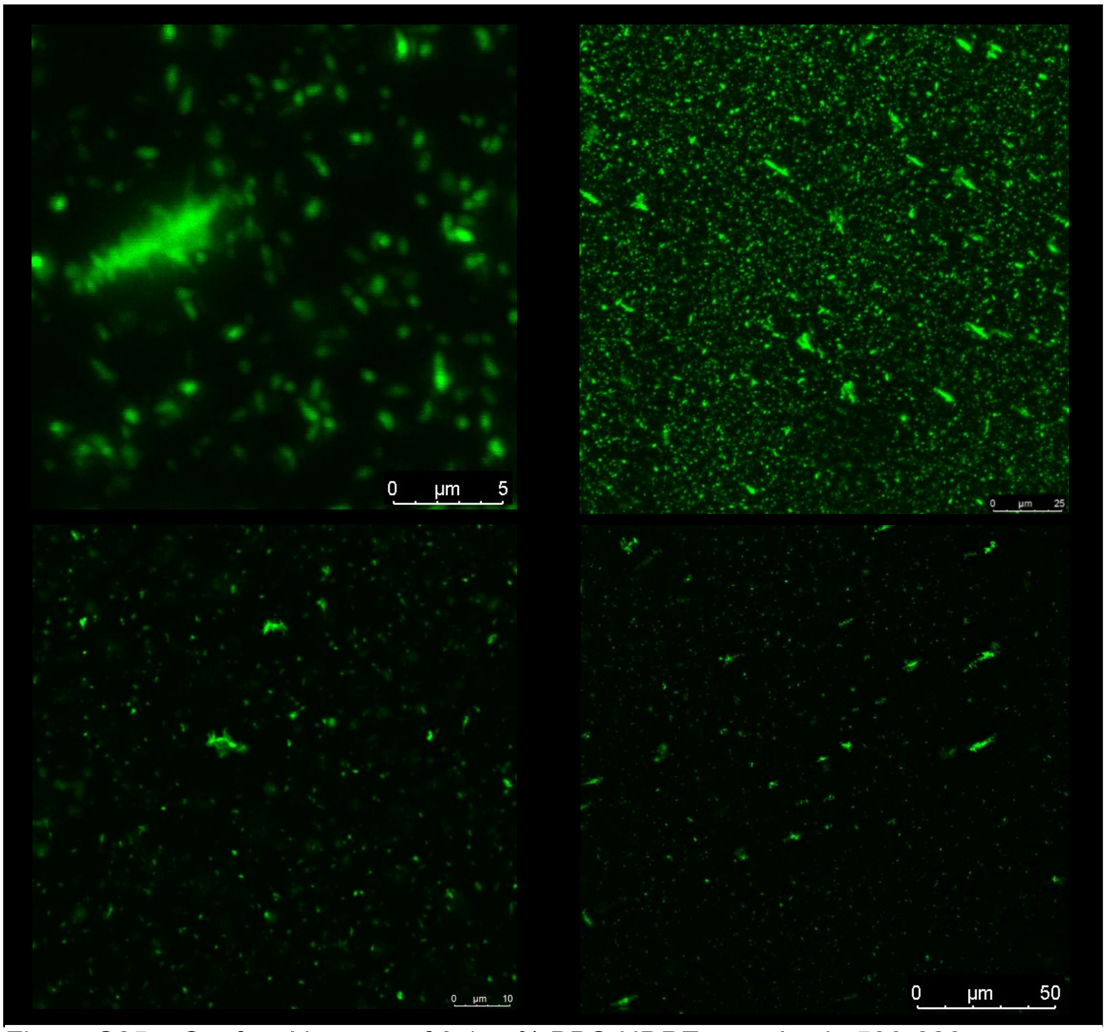

Figure S25 – Confocal images of 0.1 wt% BBS-HDPE samples in 530-620 nm range, excited at 405 nm. Top Left: Scale bar: 5 μm. Top Right: Projection through sample Scale bar: 25 μm. Bottom Left: Scale bar: 10 μm. Bottom right: Scale bar 50 μm.

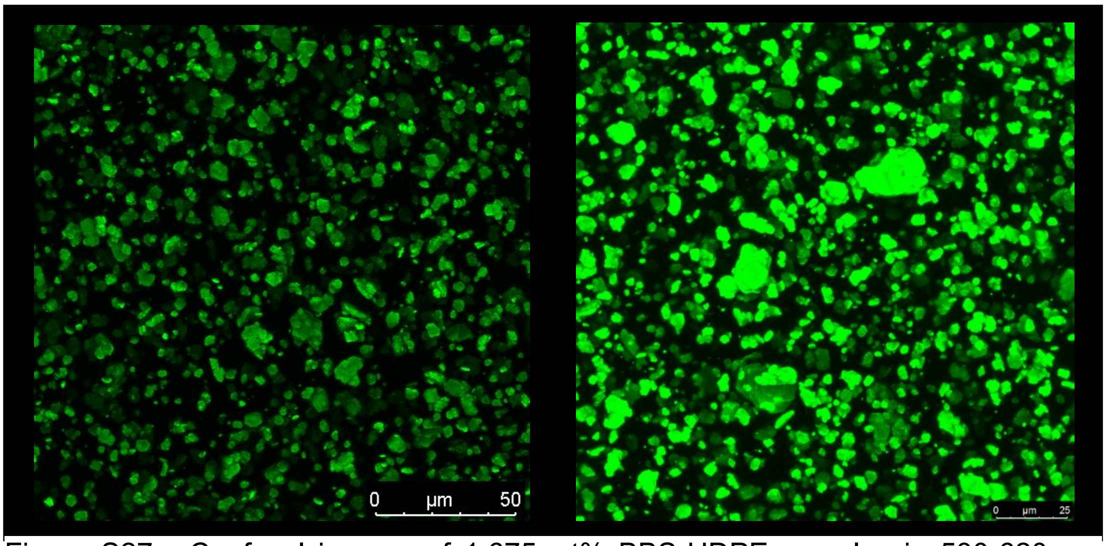

Figure S27 - Confocal images of 1.675 wt% BBS-HDPE samples in 530-620 nm range, excited at 405 nm. Left: Scale bar: 50 µm. Right: Scale bar: 25 µm.

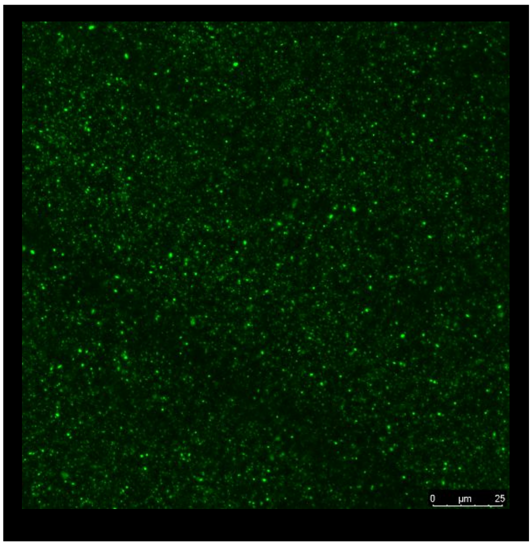

Figure S26 - Confocal images of 0.05 wt% BBS-HDPE samples in 530-620 nm range, excited at 405 nm. Scale bar: 25 µm.

Spherical aggregates were present in the 0.05 wt% and 0.1 wt% BBS-HDPE samples. Unlike at lower concentration, higher order aggregates, seemingly helical in structure, appeared in the 0.1 wt% MB. At the highest concentration of 1.675 wt%, even higher order aggregates appeared with large plate-like structures.

The average area of the aggregates detected were analysed using the ImageJ software. Aggregate area was found to increase predictably between 0.05 wt% and 0.1 wt% by almost

doubling from 0.326 μm^2 to 0.607 μm^2. The average aggregate size was found to increase non-linearly to 4.059 μm^2 at the highest concentration of 1.675 wt%. This is unsurprising due to the non-linear increase in the fluorescence intensity at 470 nm and 500 nm seen in the loading study. The presence of these larger aggregates could explain the plateauing in the lifetime parameters measured for the undiluted 0.1 wt% BBS-HDPE MB samples (SI 2.1.2. and SI 2.1.3.). By contrast, the overloaded 1.675 wt% sample displayed many well-dispersed large aggregates of around 5 μm with some as large as 20 μm (*Figure S26*). These larger aggregates were found to be plate-like in shape and more abundant than those in the 0.1 wt% sample. The increased aggregate size and abundancy with increased BBS loading corroborates the changes in fluorescence behaviour seen for the higher concentration BBS-HDPE samples (SI 1.1.1).

3.2 PP

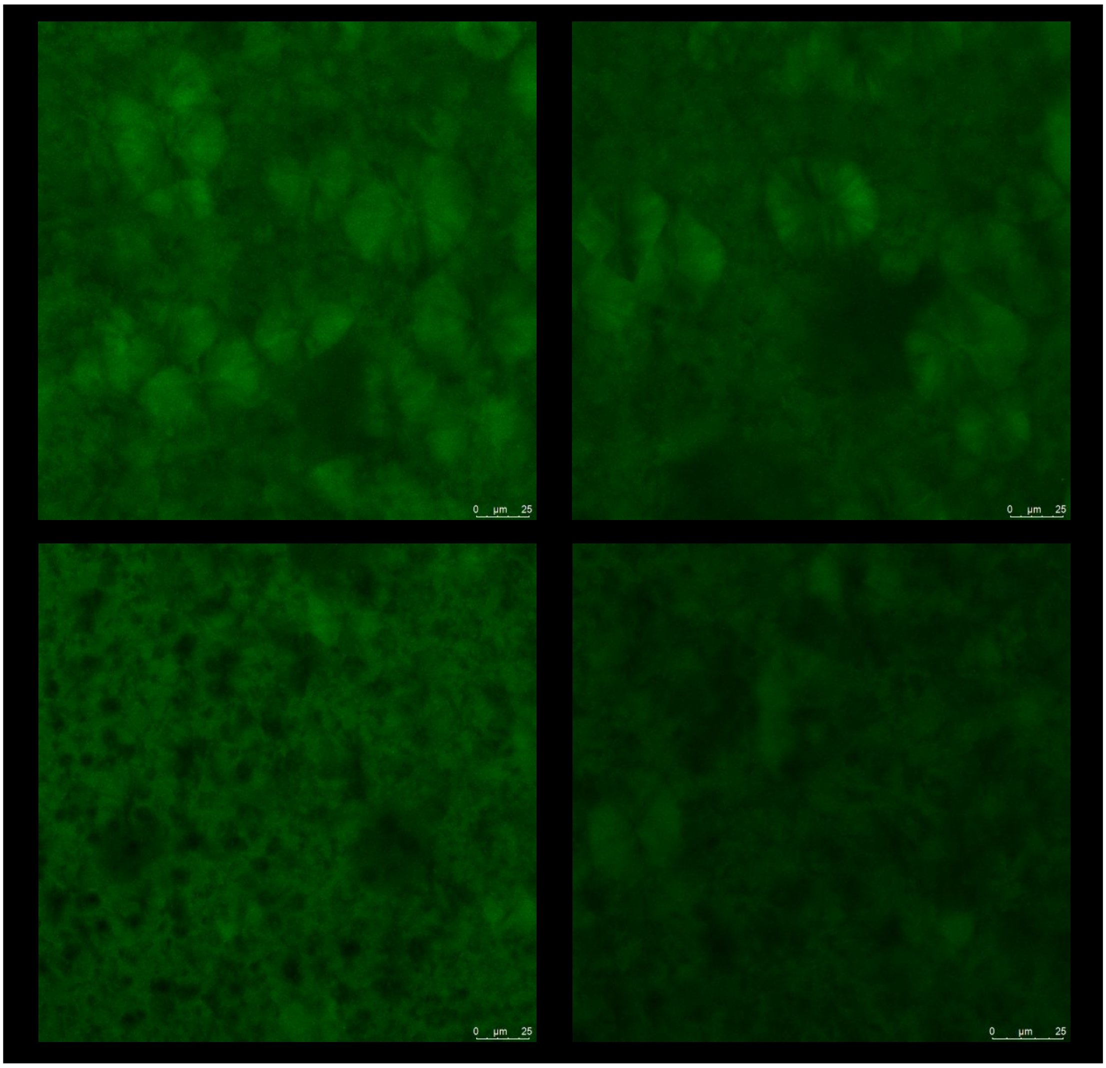

Figure S28 - Confocal images of 0.1 wt% BBS-PP samples in 530-620 nm range, excited at 405 nm. Scale bar: 25 µm.

Confocal microscopy of the 0.1 wt% MB PP BBS MB revealed a significantly different distribution of aggregates when compared to the HDPE samples. Small aggregates were detected throughout the sample which were too small to be measured using particle analysis techniques. The dye was found to be well dispersed and highlighted the presence of numerous PP spherulites. The aggregates present in PP were too small for particle size analysis.

3.3 PET

Aggregates in the annealed 0.5 wt% PET BBS MB were found to be distributed in patch-like domains of the samples. These patches were seemingly made up of infinitesimally small BBS aggregates that were too small to be measured by particle analysis methods.

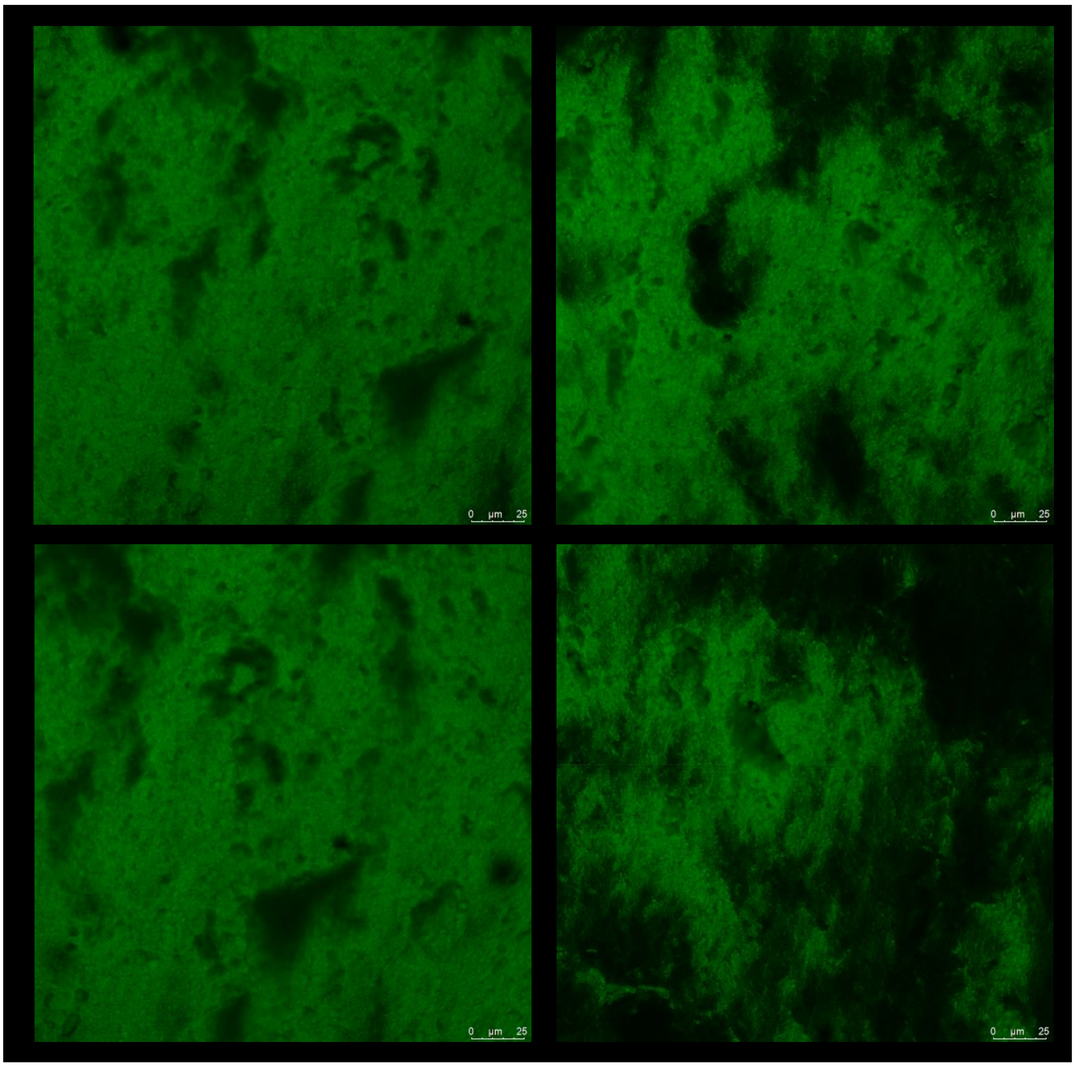

Figure S29 - Confocal images of 0.5 wt% BBS-PET samples in 530-620 nm range, excited at 405 nm. Left: Low gain setting scale bar: 25 µm.

4 FT-IR

FT-IR spectra were obtained to demonstrate that recyclate marking with BBS would have little effect on standard infra-red based plastic sorting techniques. BBS' signal relative to the main functional groups across the three polymers e.g., C-H, C=O, C-H_2, C-H_3 and C-O is imperceptible.

4.1 HDPE

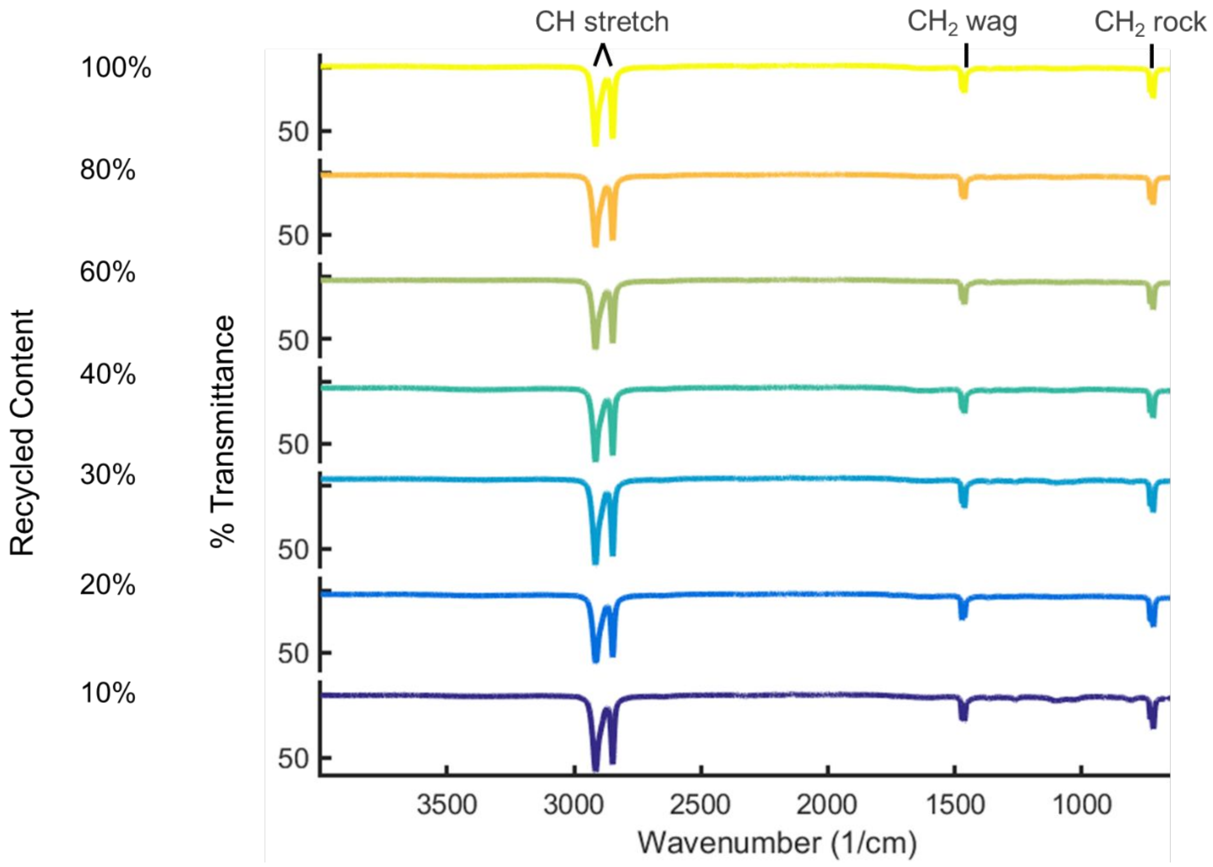

Figure S30 – FT-IR transmittance spectra of diluted 0.1 wt% BBS-HDPE MB.

4.2 PP

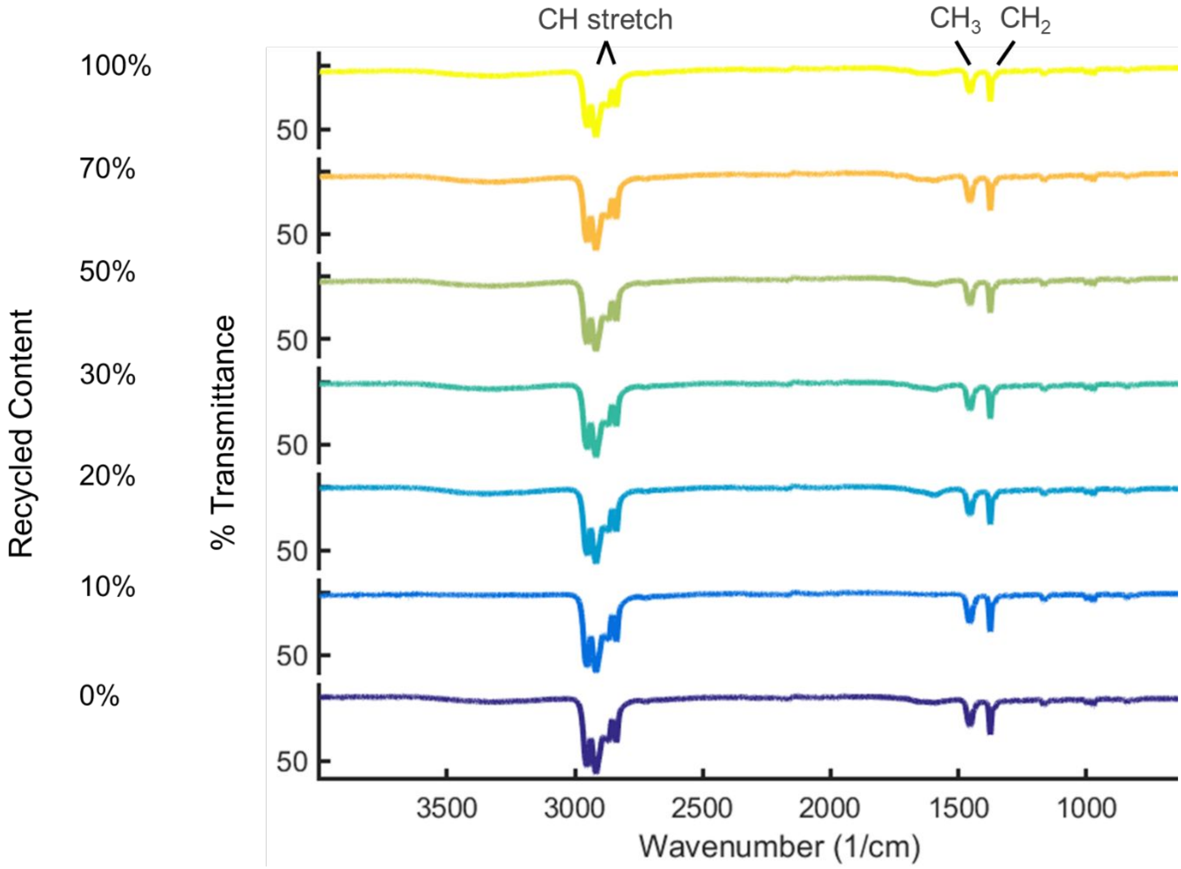

Figure S31 – FT-IR transmittance spectra of diluted 0.1 wt% BBS-PP MB.

4.3 PET

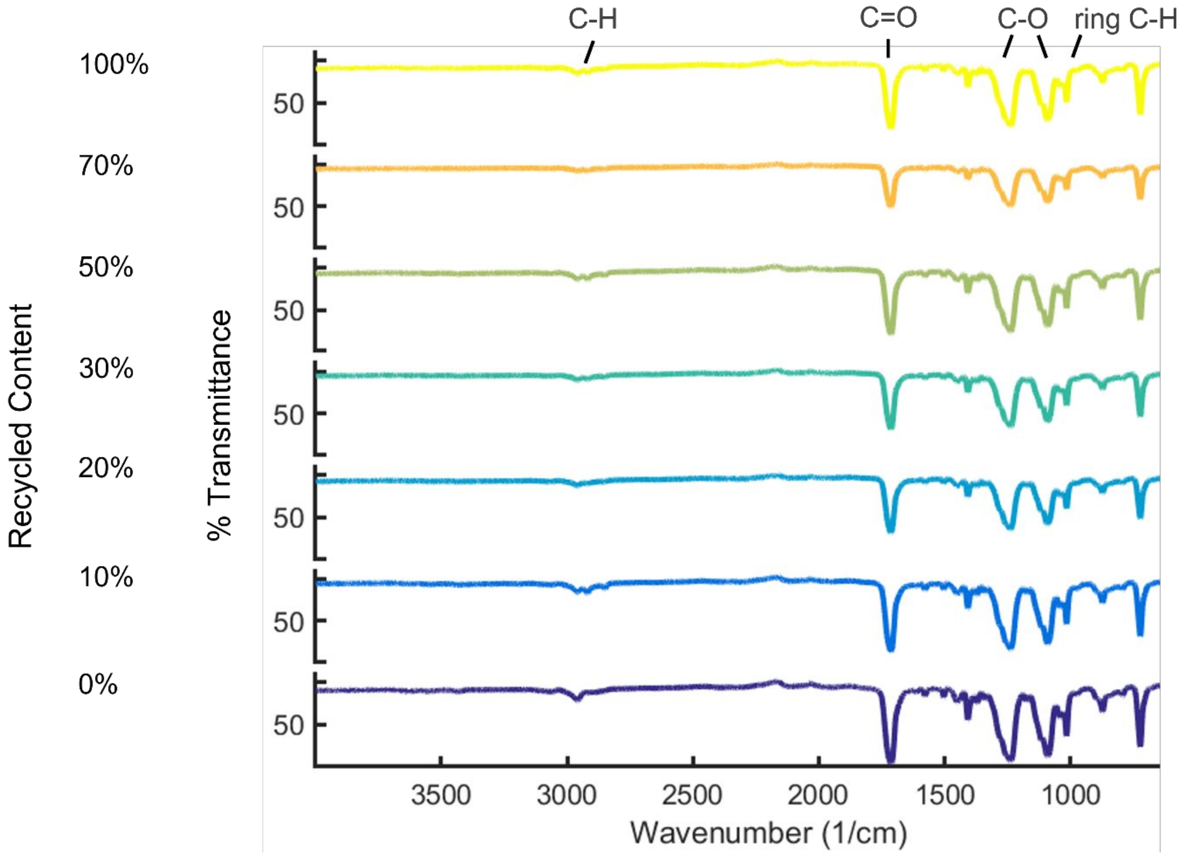

Figure S32 – FT-IR transmittance spectra of diluted 0.5 wt% BBS-PET MB.

5 DSC

5.1 Exemplary DSC Curve

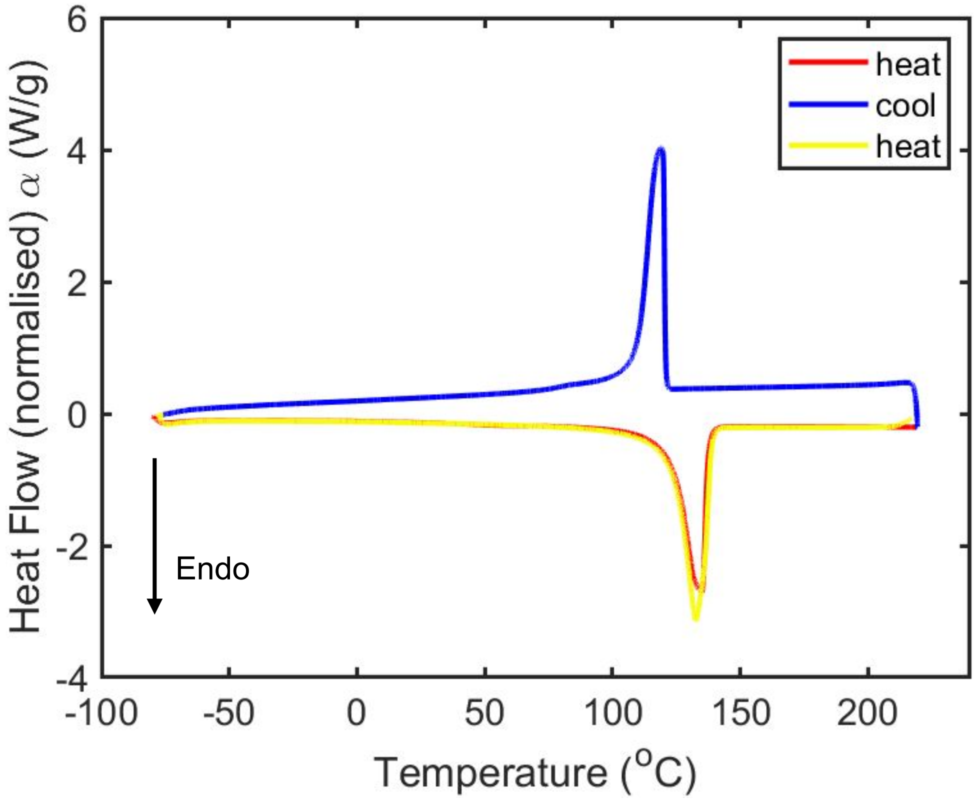

Figure S33 – Example DSC curve for BBS-HDPE samples. Heating rate 10 °C/min from -80 °C to 220 °C and 220 °C to -80 °C 5 °C/min cooling rate.

5.2 Thermal Properties

Thermal properties of HDPE, PET and PP samples marked with BBS were found to be largely unaltered. This is important in terms of material stability and of processing characteristics for re-manufacture from recyclate into new plastic products. Heating rates were fixed at 10 °C/min and cooling rates at 5 °C/min for all three polymers.

5.2.1 HDPE

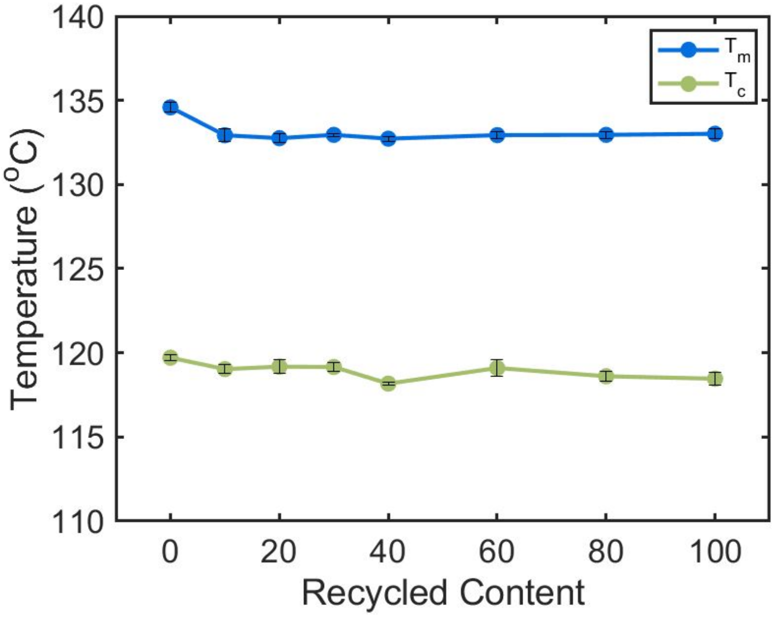

Figure S34 – Thermal properties (T_m and T_c) of diluted 0.1 wt% BBS-HDPE MB samples. Error bars represent the standard error (n = 3).

5.2.2 PP

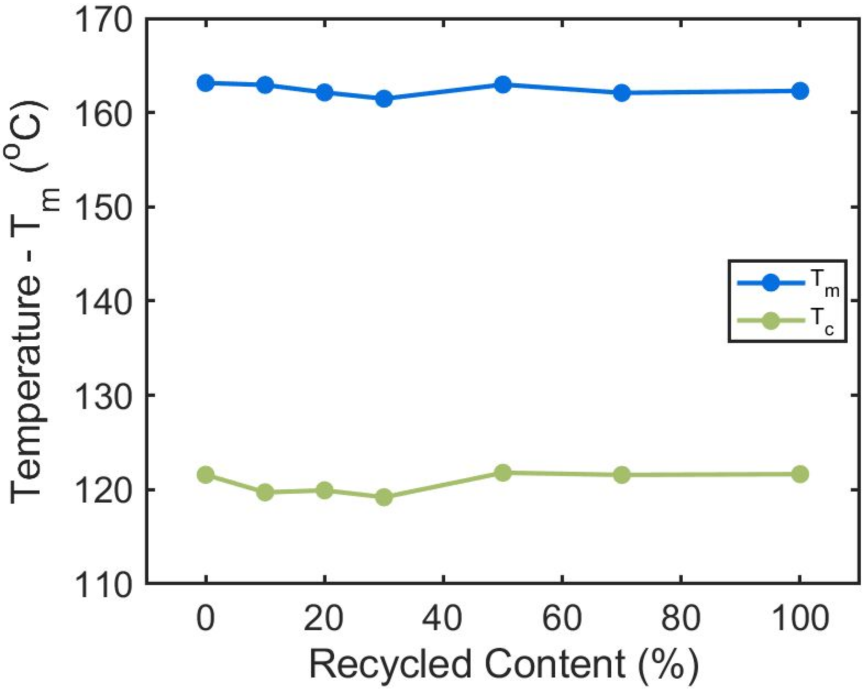

Figure S35 – Thermal properties (T_m and T_c) of diluted 0.1 wt% BBS-PP MB samples.

5.2.3 PET

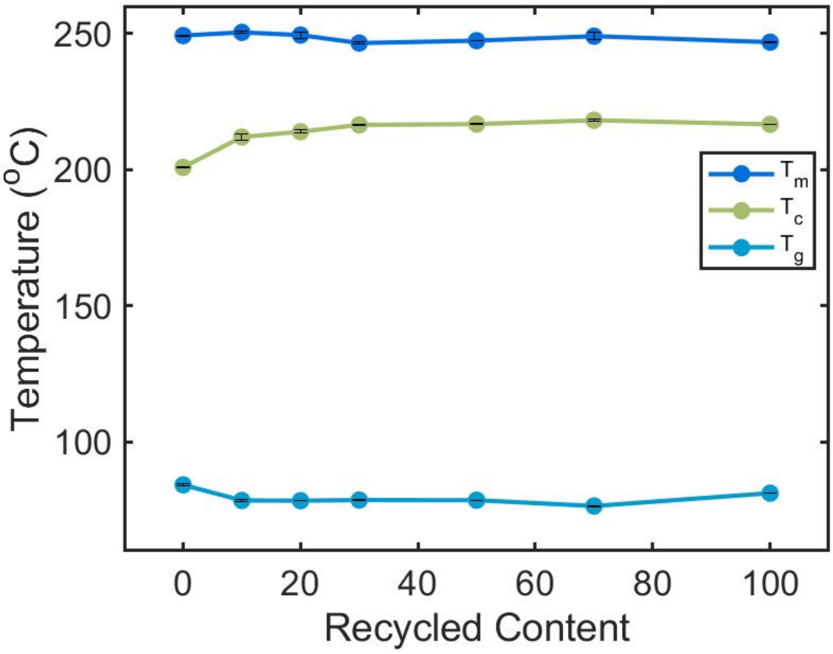

Figure S36 – Thermal properties (T_m, T_g and T_c) of diluted 0.5 wt% BBS-PET MB samples. Error bars represent the standard error (n = 3).

5.3 Crystallinity

$$\% \, Crystallinity = \frac{\Delta H_m - \Delta H_c}{\Delta H_m^o} \times 100\#(5)$$

Crystallinity of samples calculated by subtracting cold crystallisation enthalpy from melting enthalpy and dividing by melting enthalpy of perfectly crystalline HDPE (293 J/g), PET (140 J/g) and PP (207 J/g). The crystallinity of the samples was found to remain constant at all simulated recycled contents, and thus crystallinity measurements are not suitable for determining recycled content of HDPE, PET or PP. Initial jumps in crystallinity with inclusions of recycled content are expected due to thickening of the crystal lamellae due to chain scission.[6]

5.3.1 HDPE

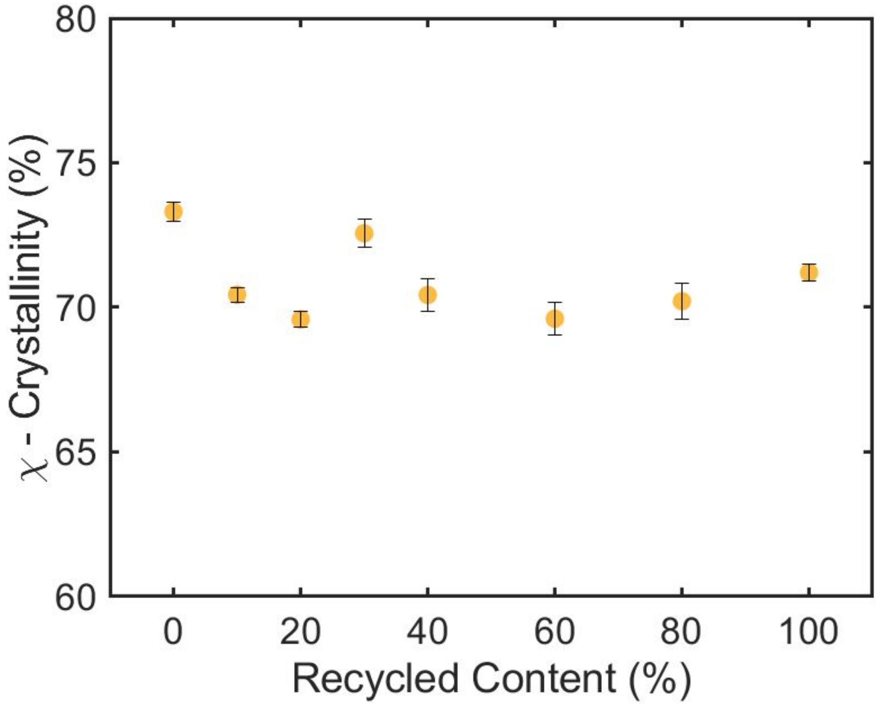

Figure S37 – Crystallinity of diluted 0.1 wt% BBS-HDPE MB samples calculated according to equation 3. Error bars represent the standard error (n = 3).

5.3.2 PET

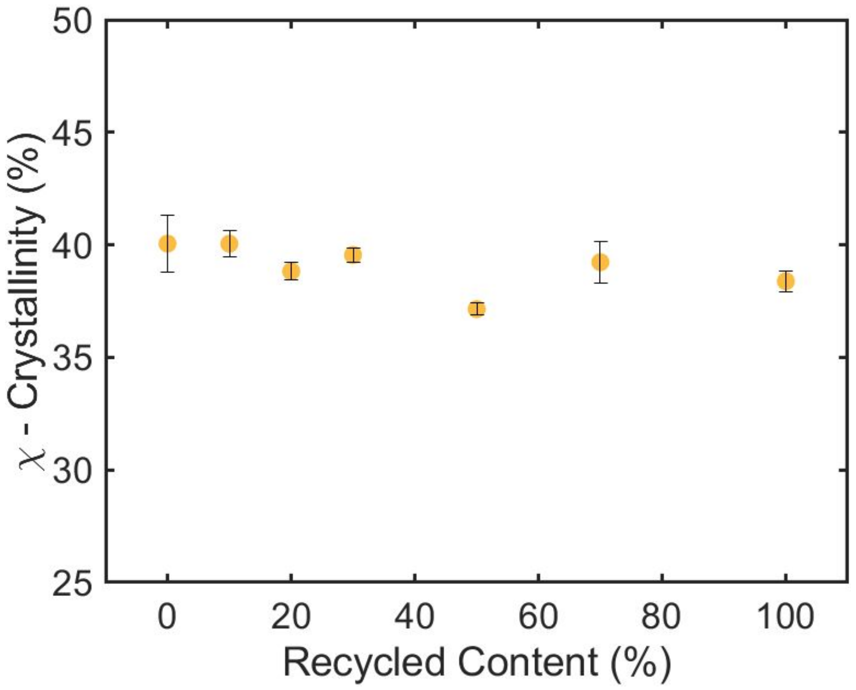

Figure S38 – Crystallinity of diluted 0.5 wt% BBS-PET MB samples calculated according to equation 3. Error bars represent the standard error (n = 3).

5.3.3 PP

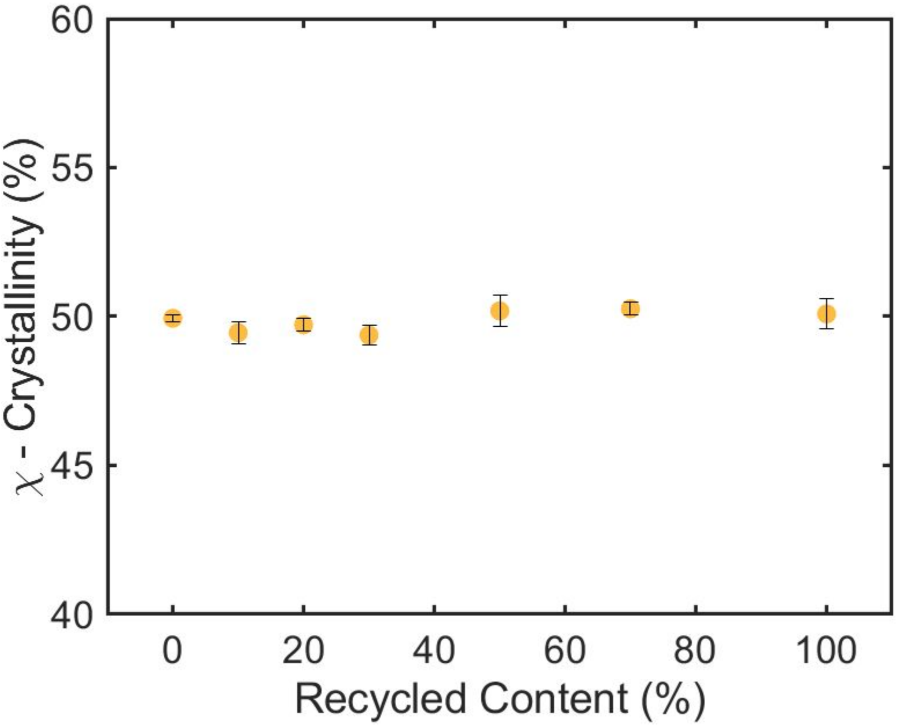

Figure S39 – Crystallinity of diluted 0.5 wt% BBS-PET MB samples calculated according to equation 3.

6 Mechanical Properties

Modulus, high ductility and fracture strength are key properties of high-quality plastics. Mechanical recycling is known to cause degradation of these properties, thus dissuading manufacturers from using recyclate. The mechanical properties of the marked samples were tested to ensure that incorporation of BBS had little effect on the final properties of the materials.

6.1 Elongation at Break

6.1.1 HDPE

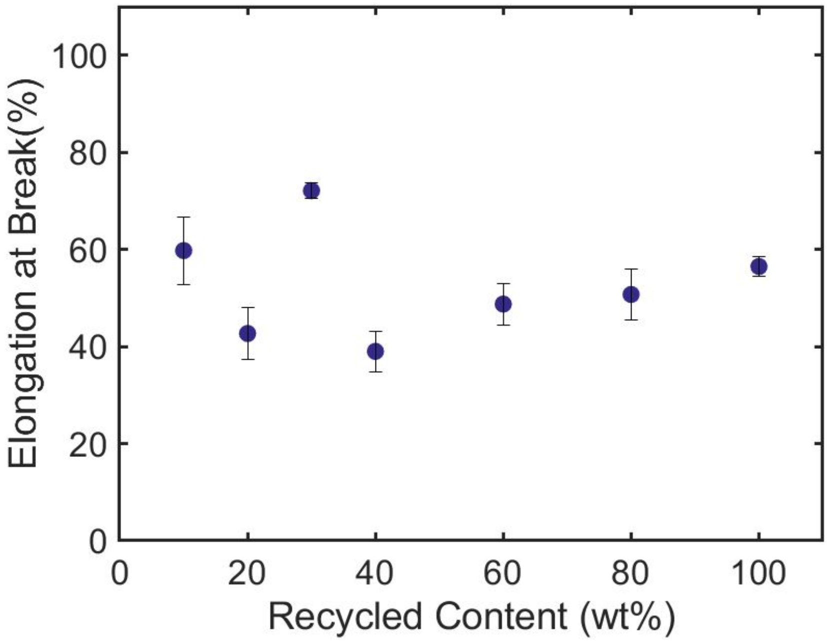

Figure S40 – Elongation at break of diluted 0.1 wt% BBS-HDPE MB samples measured at 5 mm/min. Error bars represent the standard error (n = 5). Fitting performed using the MATLAB curve fitting toolbox.

6.1.2 PP

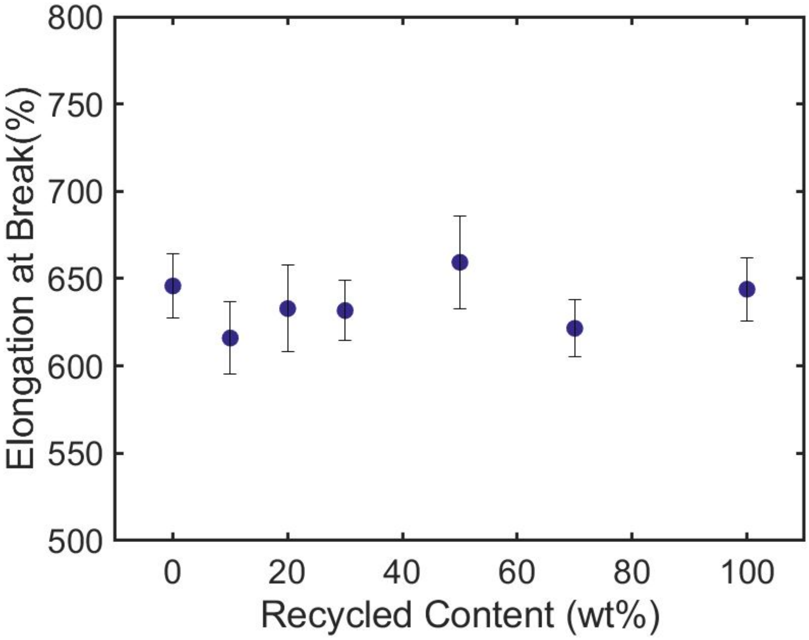

Figure S41 – Elongation at break of diluted BBS-PP 0.1 wt% MB samples measured at 15 mm/min. Error bars represent the standard error (n = 5). Fitting performed using the MATLAB curve fitting toolbox.

6.1.3 PET

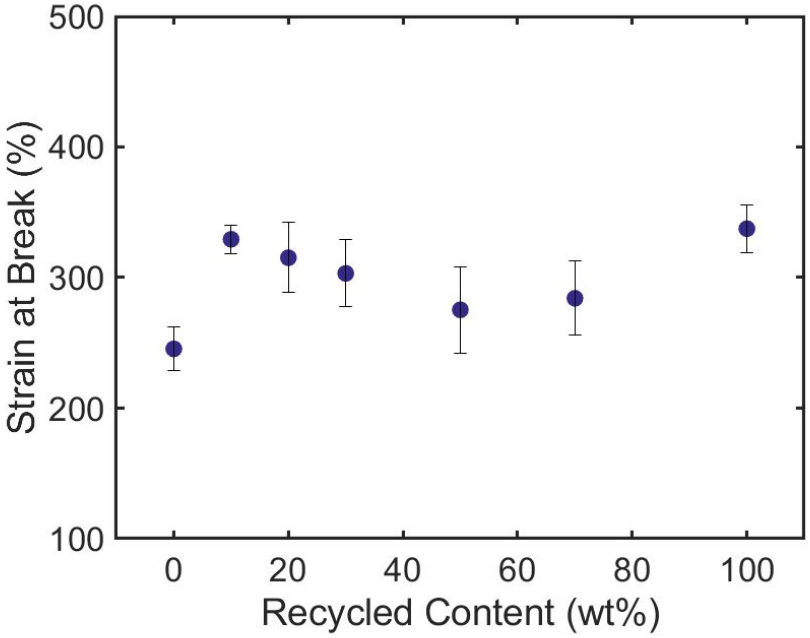

Figure S42 – Elongation at break of diluted BBS-PET 0.5 wt% MB samples measured at 40 %/min. Error bars represent the standard error (n = 5). Fitting performed using the MATLAB curve fitting toolbox.

6.2 Young's Modulus

6.2.1 HDPE

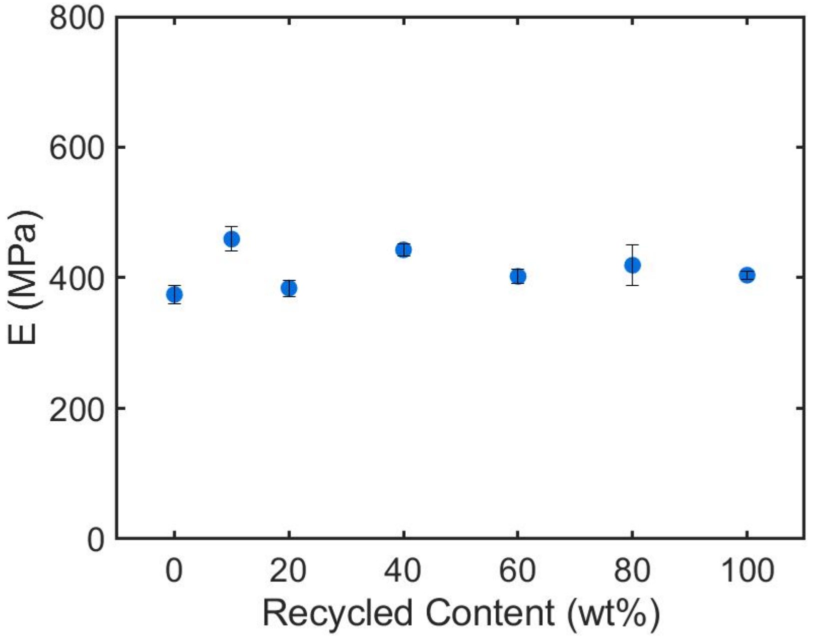

Figure S43 – Young's Modulus of diluted 0.1 wt% BBS-HDPE MB samples calculated according to equation 3. Error bars represent the standard error (n = 5). Fitting performed using the MATLAB curve fitting toolbox setting elastic regime fit limits between 0 and 5 % displacement.

6.2.2 PP

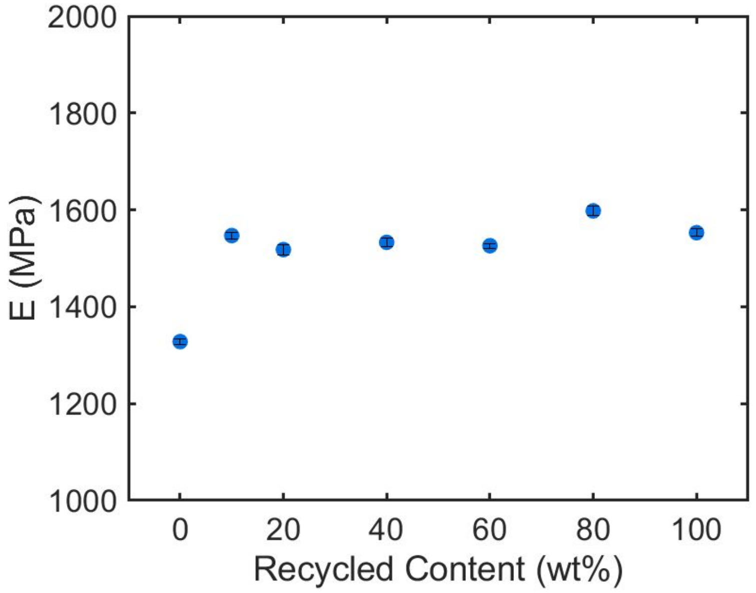

Figure S44 – Young's Modulus of diluted 0.1 wt% BBS-PP MB samples calculated according to equation 3. Error bars represent the standard error (n = 5). Fitting performed using the MATLAB curve fitting toolbox setting elastic regime fit limits between 0 and 5 % displacement.

6.2.3 PET

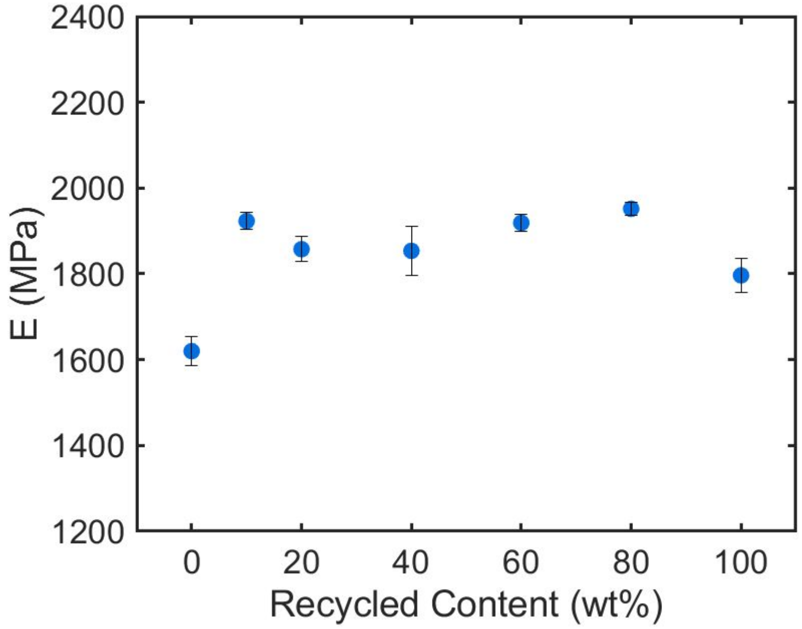

Figure S45 – Young's Modulus of diluted 0.5 wt% BBS-PET MB samples calculated according to equation 3. Error bars represent the standard error (n = 5). Fitting performed using the MATLAB curve fitting toolbox setting elastic regime fit limits between 0 and 5 % displacement.

7 Rheological Properties

The rheological properties of polymers at melt temperature can be indicative of their response to processing. Processing or mechanical uses heat and shear and these processes can be simulated using rheological measurements. Complex viscosity, storage and loss moduli were measured at a low strain rate to remain in the linear viscoelastic region. The rheological behaviour of all samples was found to be unchanged on incorporation of BBS.

7.1 HDPE

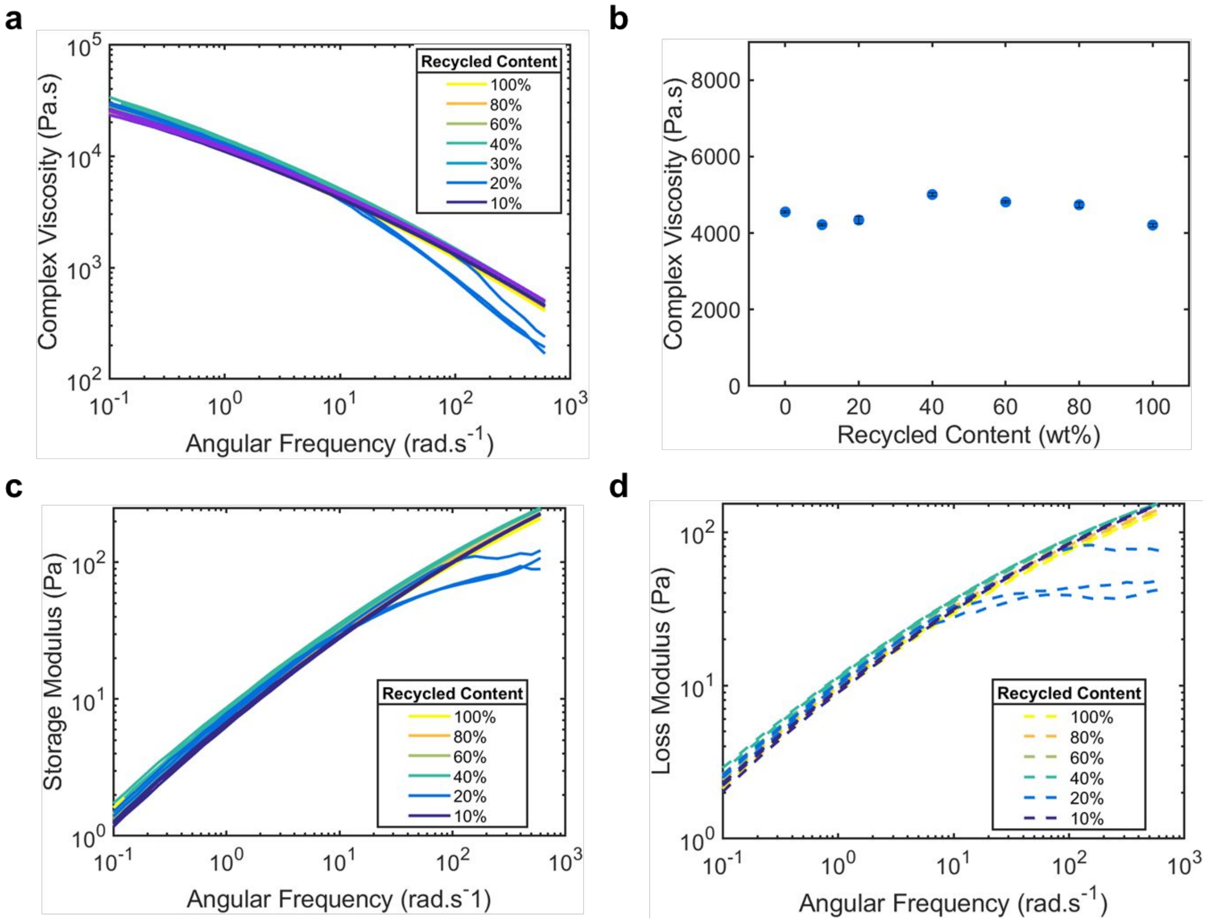

Figure S46 – Rheological properties of diluted 0.1 wt% BBS-HDPE MB. (**A**)Complex viscosity with varying angular frequency for the diluted 0.1 wt% MB. (**B**) Complex viscosity measured at 10 rad·s^{-1} for diluted 0.1 wt% MB. (**C**) Storage moduli with varying angular frequency for the diluted 0.1 wt% MB. (**D**) Loss moduli with varying angular frequency for the diluted 0.1 wt% MB.

7.2 PET

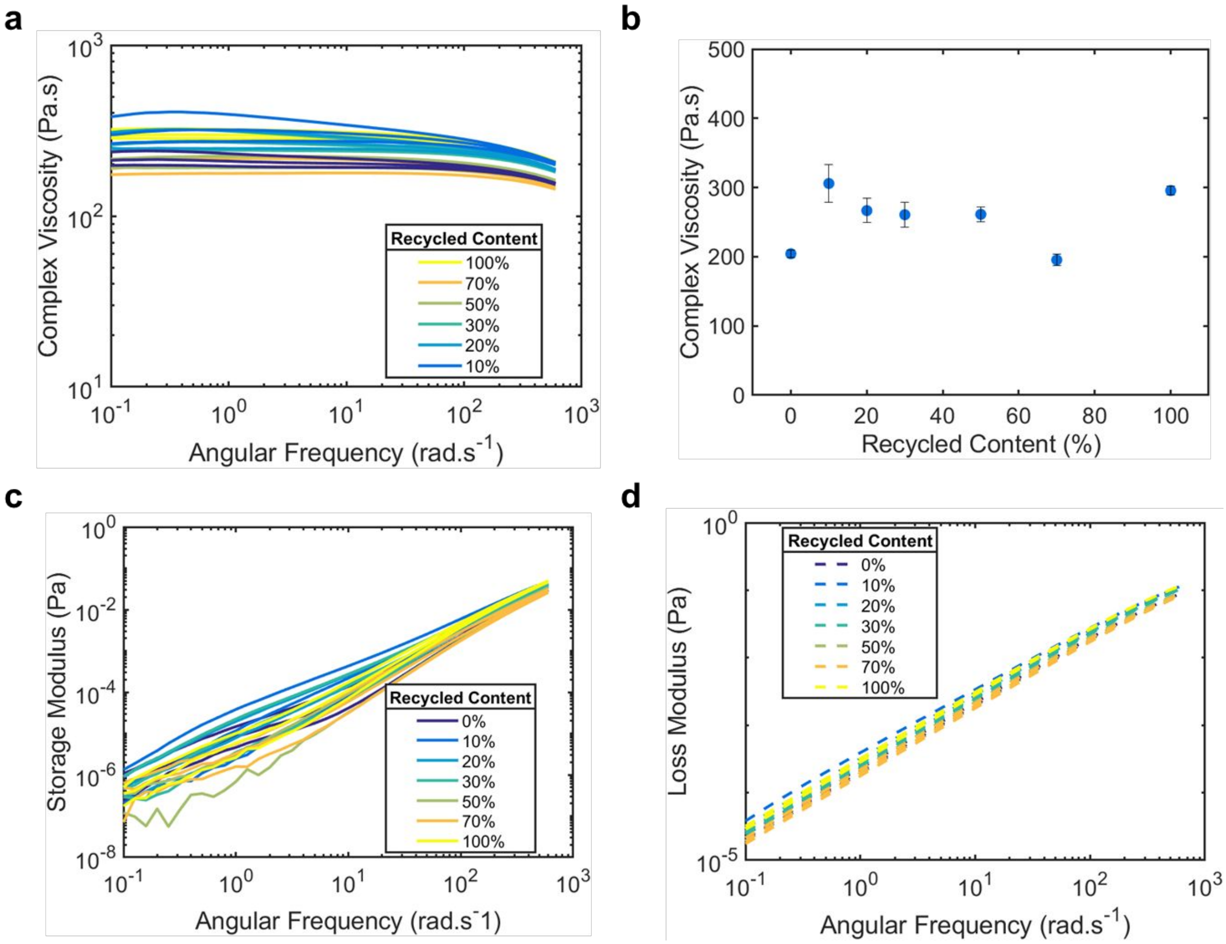

Figure S47 – Rheological properties of diluted 0.5 wt% BBS-PET MB. (**A**) Complex viscosity with varying angular frequency for the diluted 0.5 wt% MB. (**B**) Complex viscosity measured at 10 rad·s-1 for diluted 0.5 wt% MB. (**C**) Storage moduli with varying angular frequency for the diluted 0.5 wt% MB. (**D**) Loss moduli with varying angular frequency for the diluted 0.5 wt% MB.

7.3 PP

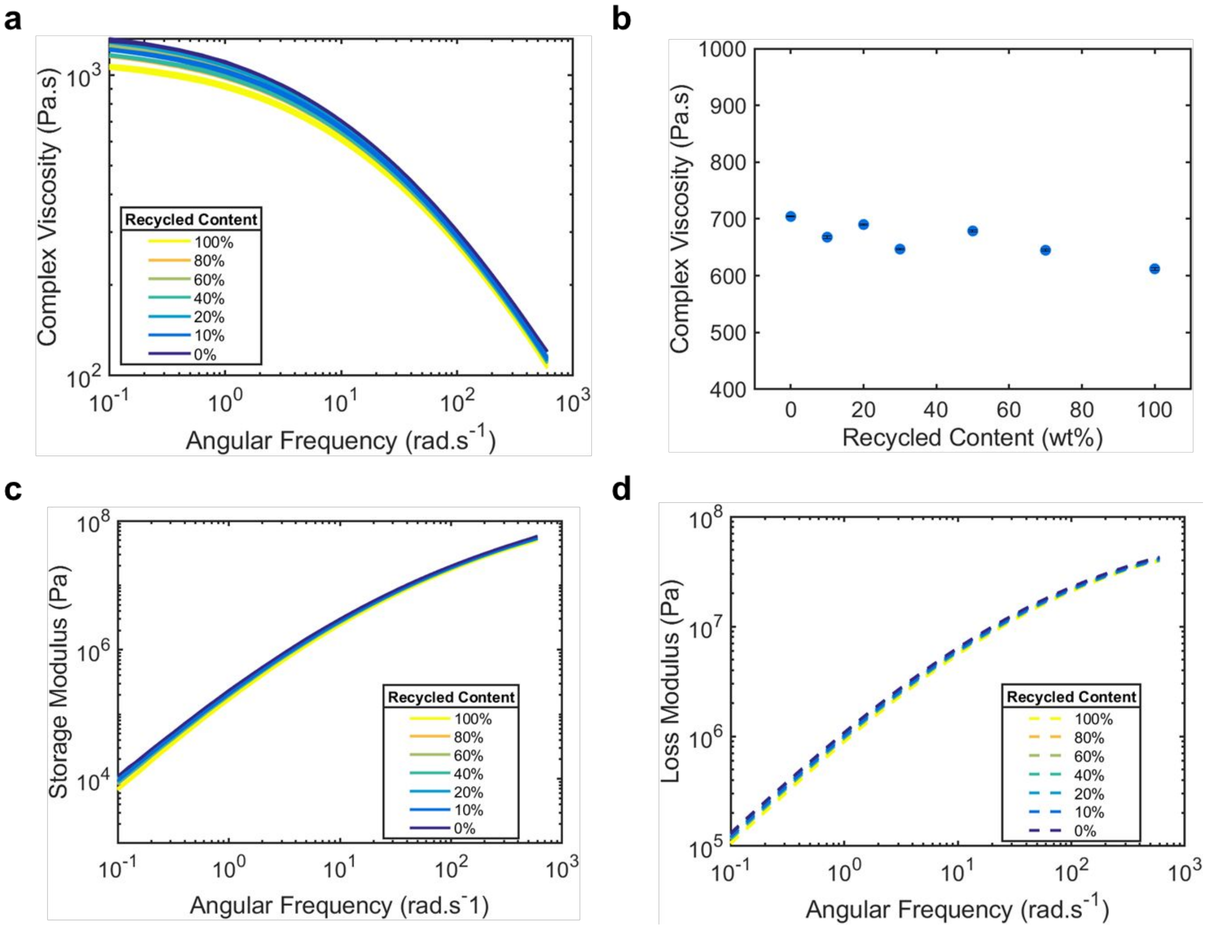

Figure S48 – Rheological properties of diluted 0.1 wt% BBS-PP MB. (**A**) Complex viscosity with varying angular frequency for the diluted 0.1 wt% MB. (**B**) Complex viscosity measured at 10 rad·s-1 for diluted 0.1 wt% MB. (**C**) Storage moduli with varying angular frequency for the diluted 0.1 wt% MB. (**D**) Loss moduli with varying angular frequency for the diluted 0.1 wt% MB.

8 Colour Analysis

8.1 HDPE

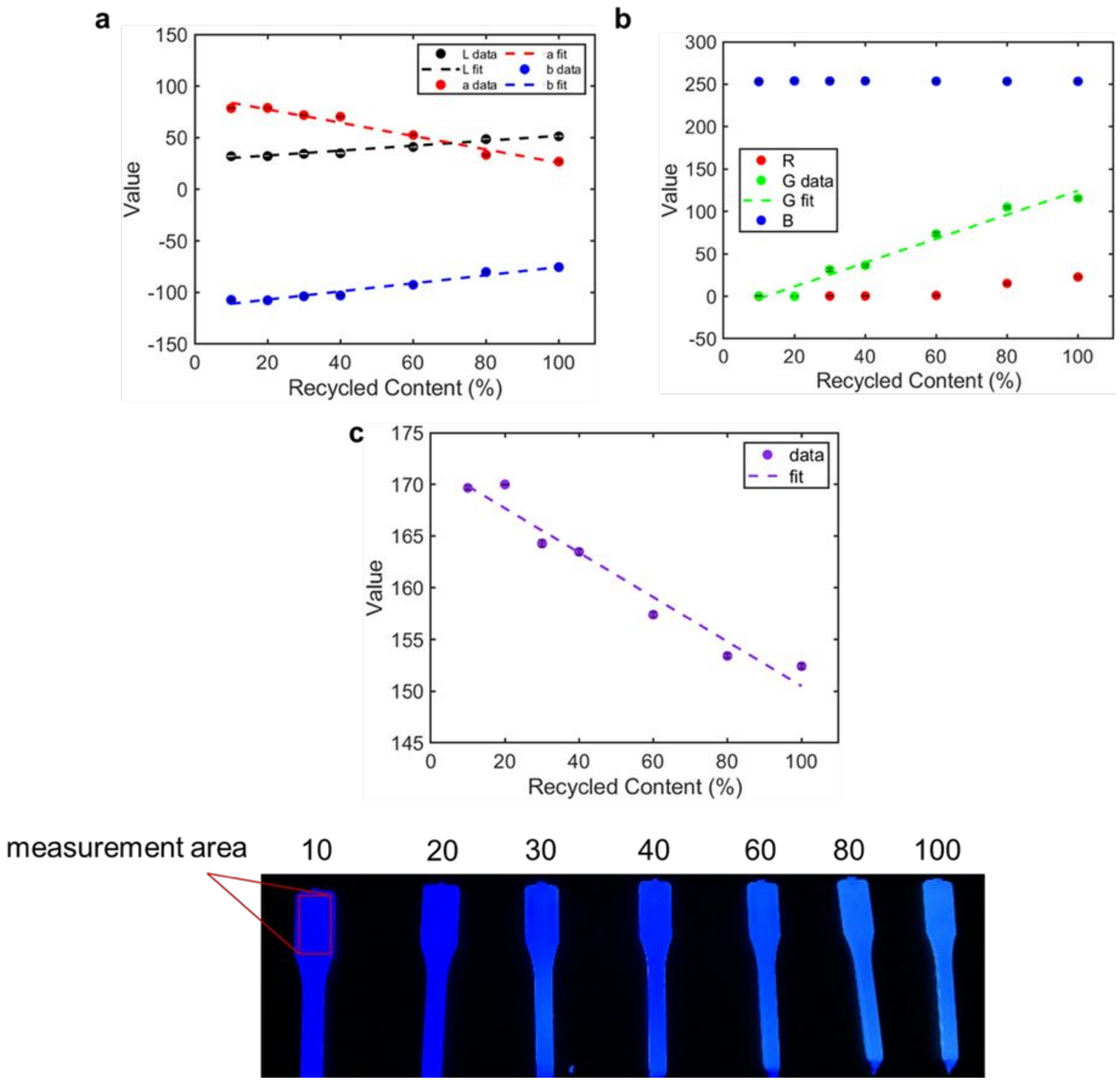

Figure S49 - Colour analysis of 0.1 wt% BBS-HDPE MB samples illuminated at 365 nm and photographed in a blacked-out room. (**A**) L*a*b* values measured using ImageJ. Fits produced using MATLAB curve fitting toolbox (L (R^2 = 0.9511), a (R^2 = 0.9660), b (R^2 = 0.9609)). (**B**) RGB values measured using ImageJ. Fits produced using MATLAB curve fitting toolbox (G (R^2 = 0.9718)). (**C**) Hue values measured using ImageJ. Fits produced using MATLAB curve fitting toolbox (Hue (R^2 = 0.9511)). Errors taken as the standard deviation produced by the ImageJ software.

Comparative colour differences between reference HDPE and the dyed samples under identical illumination conditions allowed direct comparison of colour differences due to aggregation. Increases in b* values (~-110 to ~-75) and decreases in a* values (~75 to ~30)

were also observed, corresponding to increases in yellow and green content, respectively. This was reinforced by increasing G values (~0 to ~120) and decreasing hue (~170 to ~155) with increasing recycled content. Linear correlations were found between recycled content and a*, b*, G and hue values for the BBS-HDPE MB (*Figure S49*).

8.2 PP

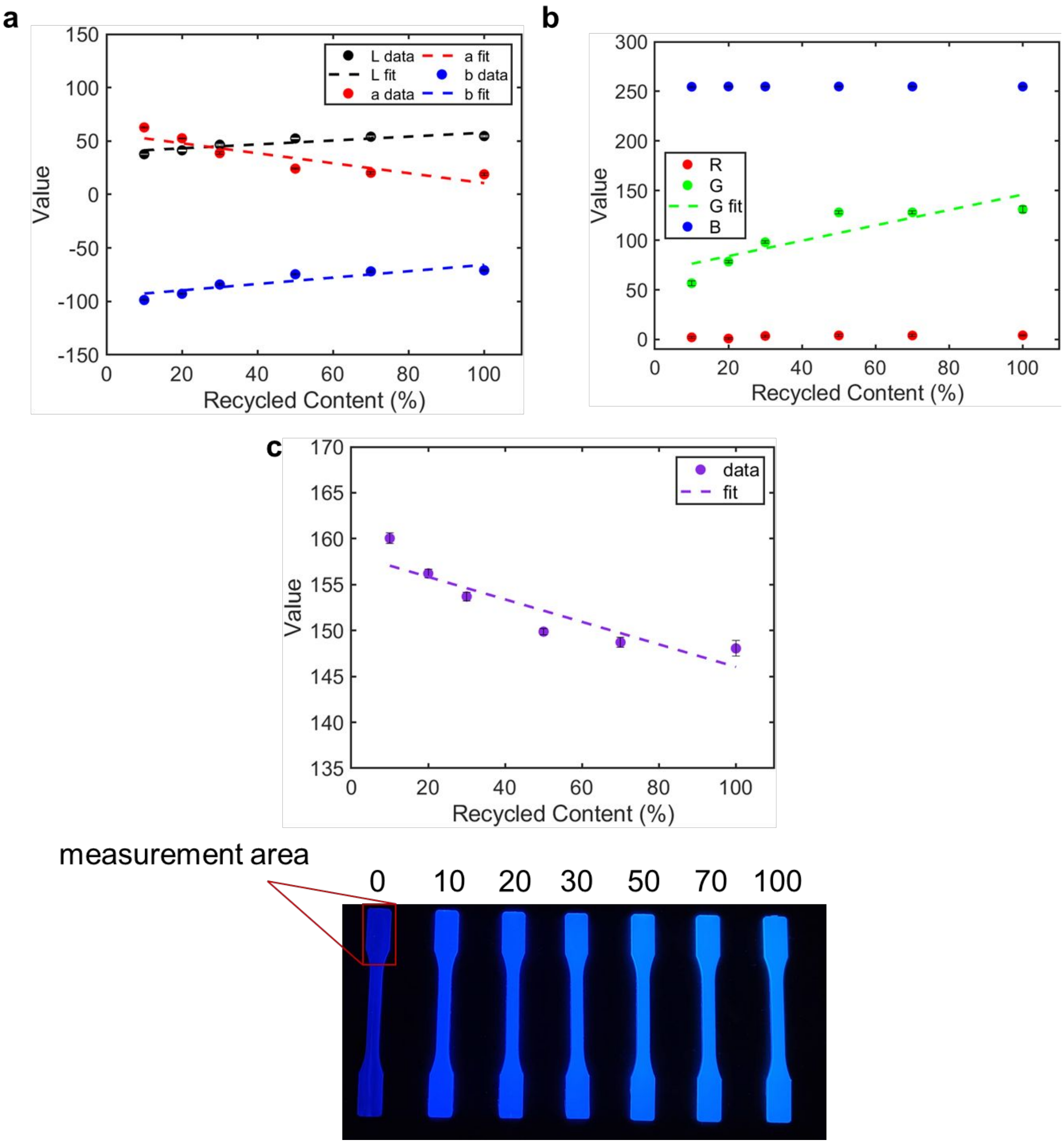

Figure S50 – Colour analysis of 0.1 wt% BBS-PP MB samples illuminated at 365 nm and photographed in a blacked-out room. (**A**) L*a*b* values measured using ImageJ. (L (R^2 = 0.8578), a (R^2 = 0.8479), b (R^2 = 0.8570)). (**B**) RGB values measured using ImageJ. (G (R^2 = 0.8143)). (**C**) Hue values measured using ImageJ (Hue (R^2 = 0.8724)). Errors taken as the standard deviation produced by the ImageJ software. Fits produced using MATLAB curve fitting toolbox.

8.3 PET

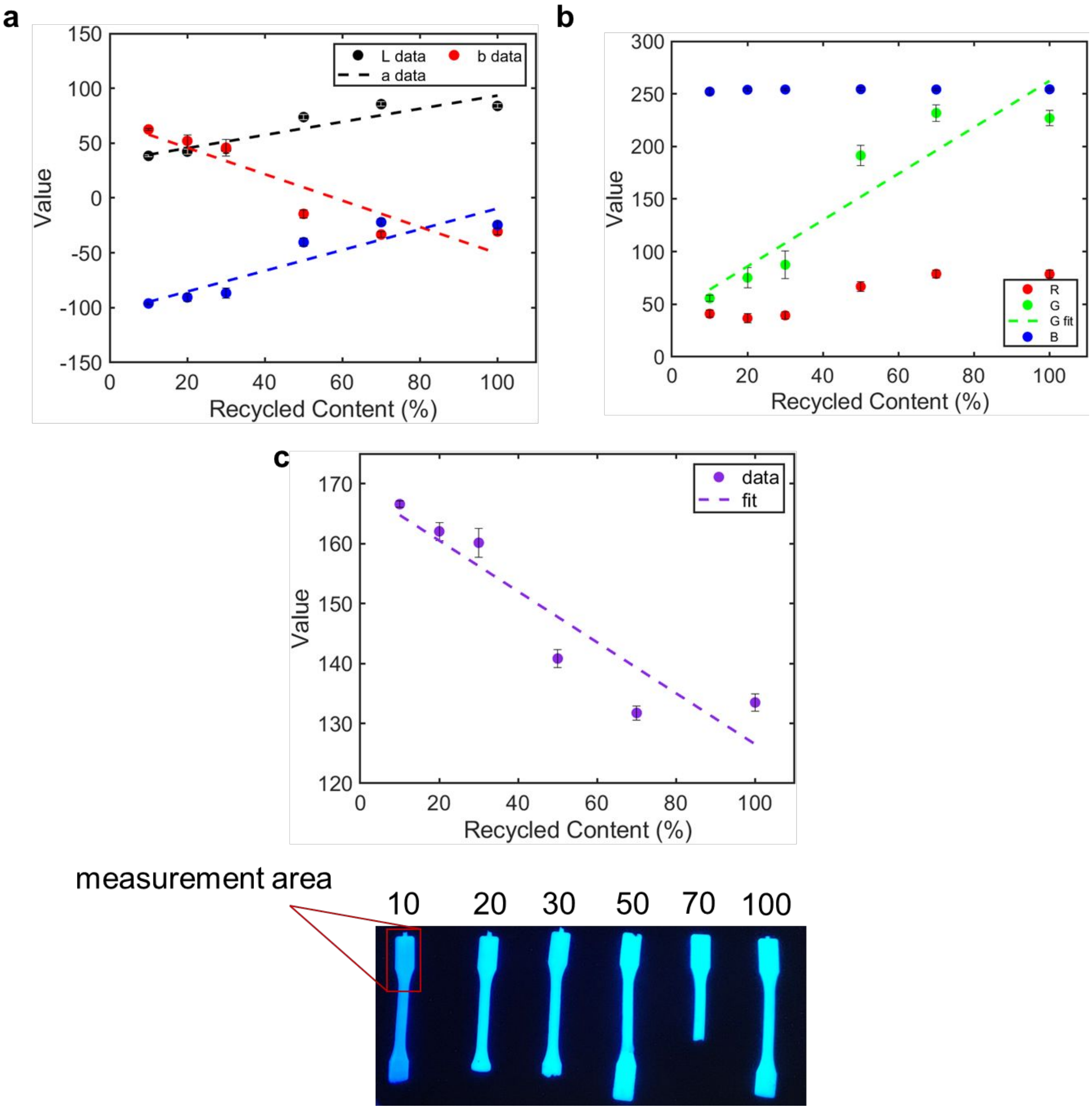

Figure S51 – Colour analysis of annealed 0.5 wt% BBS-PET MB samples illuminated at 365 nm and photographed in a blacked-out room. (**A**) L*a*b* values measured using ImageJ. (L (R^2 = 0.8530), a (R^2 = 0.8435), b (R^2 = 0.8506)). (**B**) RGB values measured using ImageJ (G (R^2 = 0.8563)). (**C**) Hue values measured using ImageJ. (Hue (R^2 = 0.8560)). Errors taken as the standard deviation produced by the ImageJ software. Fits produced using MATLAB curve fitting toolbox.

9 TGA of 4,4′-bis(2-benzoxazolyl)stilbene

Thermogravimetric analysis was performed to investigate the thermal stability of the BBS dye. With a $T_{d5\%}$ of 367.60 °C, BBS dye degrades at a higher temperature than the processing temperature ranges for all packaging plastics, confirming its suitability for recyclate marking in common plastics.[7]

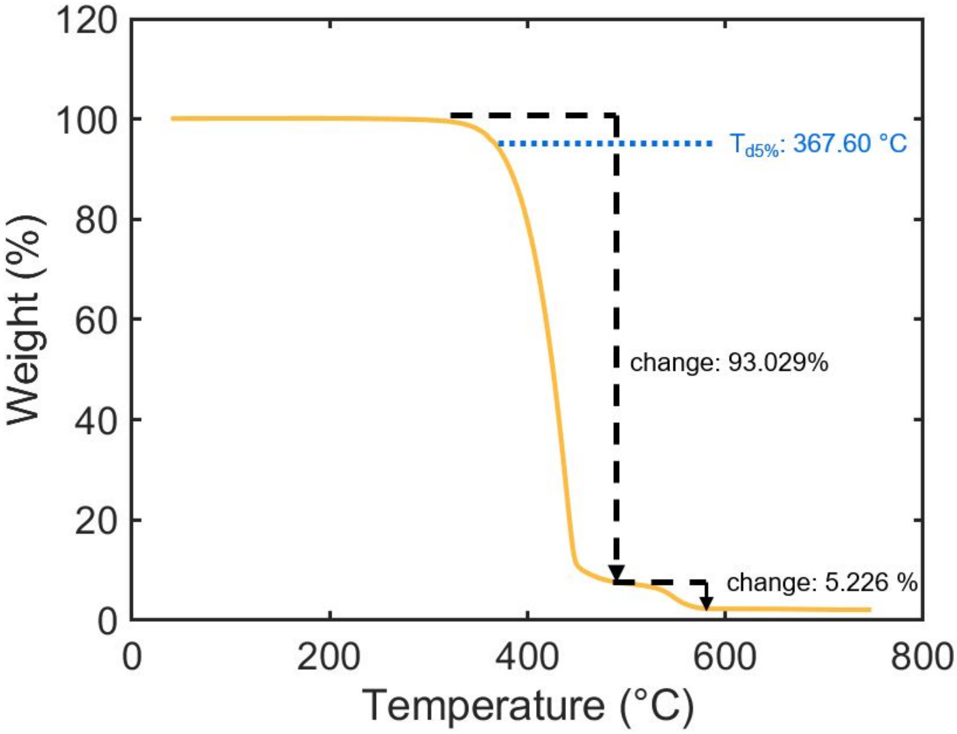

Figure S52 – TGA of BBS in air ramped at 10 °C/min. $T_{d5\%}$ at 367.60 °C.

10 Water Vapour Transmission Rates

Water vapour transmission rate (WVTR) measurements were performed on virgin PP and 0.1 wt% BBS-PP blown films to investigate the barrier properties of the film. PP and the BBS-PP 0.1 wt% MB of 40 μm thickness were tested at 37.8 °C at 90 % relative humidity with medium conditioning settings (> 10 [g/(m^2·24h)]) according to ASTM F1249.[8] A small improvement in WVTR was found when BBS was present within the PP film (WVTRs 5.598 ± 0.029 and 7.161 ± 0.409 [g/(m^2 · 24h)] for BBS-PP and PP film respectively).

11 Recycling Simulations of Coloured HDPE Samples

11.1 Fluorescence Emission

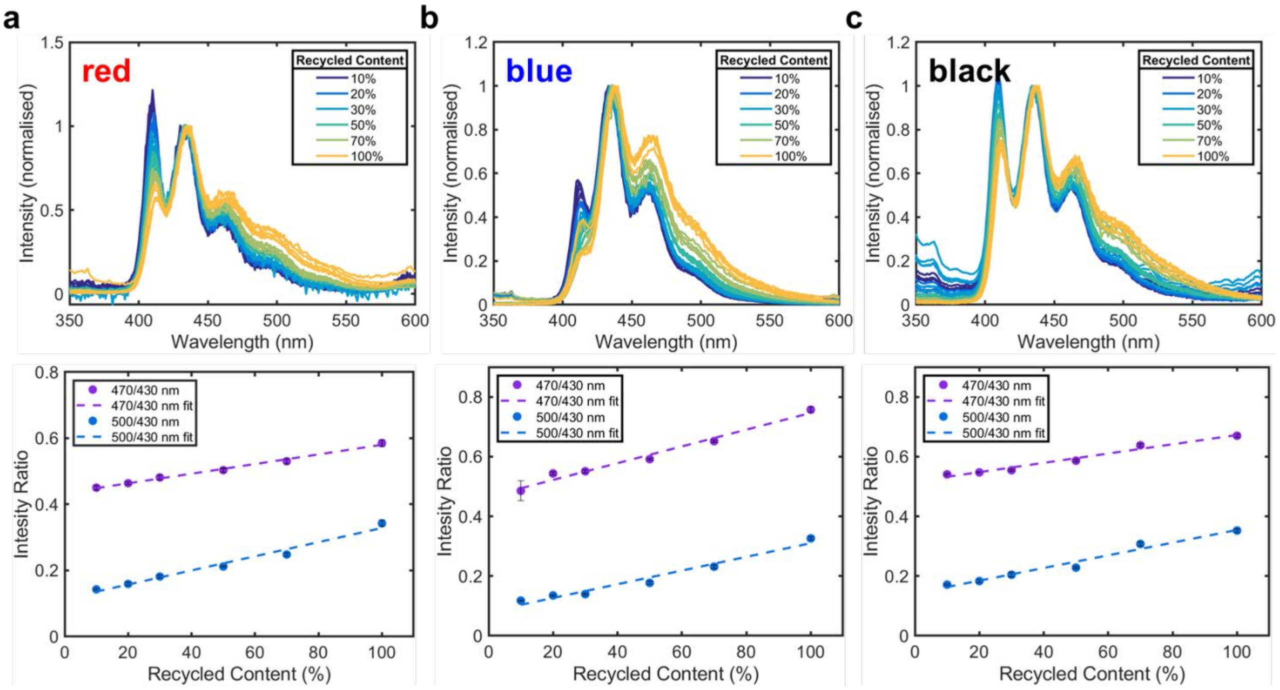

Figure S53 – (**A**) Top: Fluorescence emission spectra of varying recycled content for red HDPE recyclate marked by BBS, normalised to 1 at the fluorescence emission maximum of isolated molecules. Bottom: Resulting intensity ratios for red diluted 0.1 wt% BBS-HDPE samples between 470, 500 and 430 nm. (470/430 nm R^2 = 0. 9928 and 500/430 nm R^2 = 0.9773. (**B**) Top: Fluorescence emission spectra of varying recycled content for blue HDPE recyclate marked by BBS, normalised to 1 at the fluorescence emission maximum of isolated molecules. Bottom: Resulting intensity ratios for blue diluted 0.1 wt% HDPE BBS samples between 470, 500 and 430 nm. (470/430 nm R^2 = 0. 9786 and 500/430 nm R^2 = 0. 9659. (**C**) Top: Fluorescence emission spectra of varying recycled content for black HDPE recyclate marked by BBS, normalised to 1 at the fluorescence emission maximum of isolated molecules. Bottom: Resulting intensity ratios for black diluted 0.1 wt% HDPE BBS samples between 470, 500 and 430 nm. (470/430 nm R^2 = 0.9735 and 500/430 nm R^2 = 0.9716. Error bars represent the standard error (n = 5) where each sample comes from the same batch. Fits produced using the MATLAB curve fitting toolbox.

11.2 Optical analysis

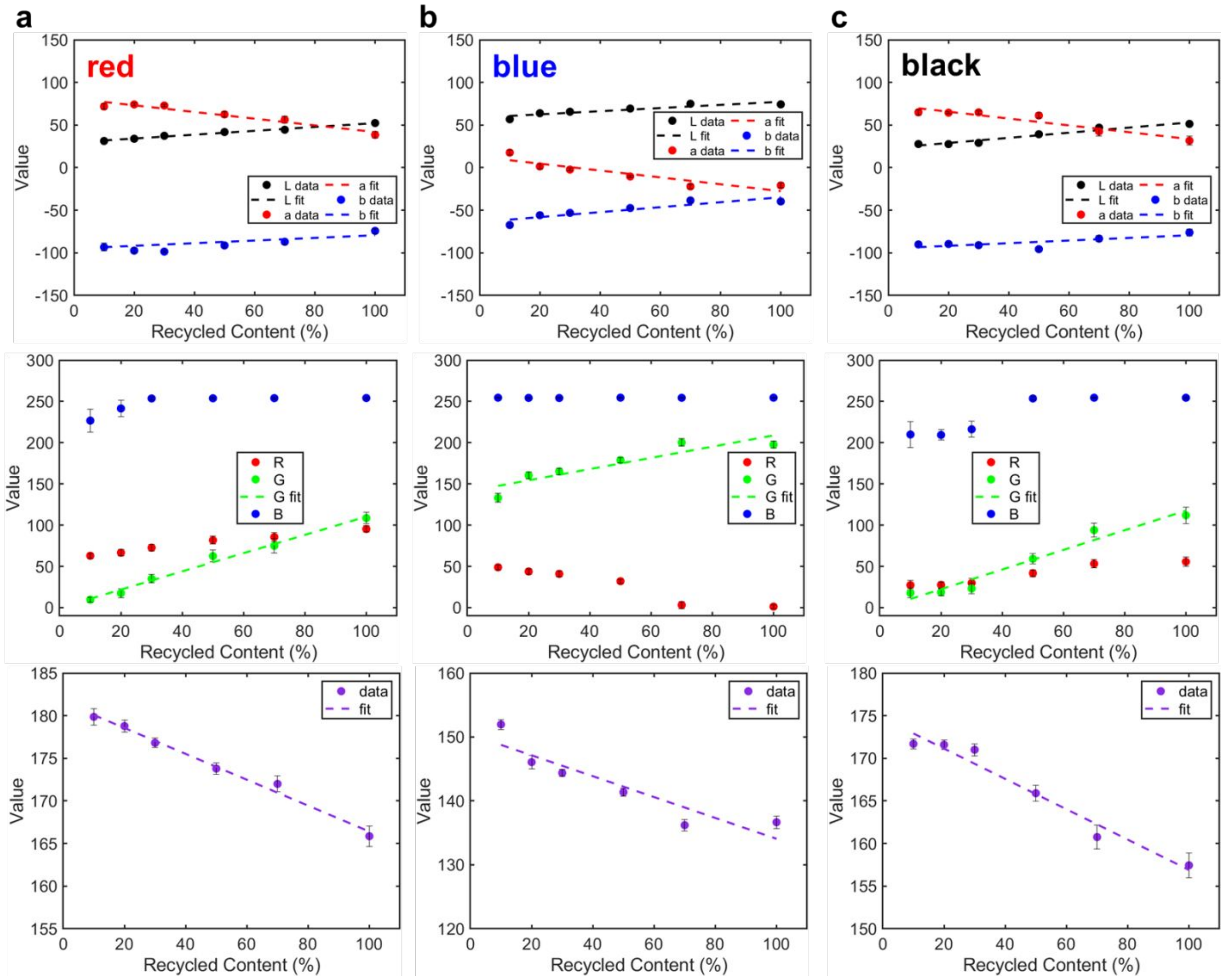

Figure S54 - Colour analysis of 0.1 wt% BBS-HDPE MB coloured samples illuminated at 365 nm and photographed in a blacked-out room. (**A**) L*a*b*, RGB and Hue values of red samples measured using ImageJ. (**B**) L*a*b*, RGB and Hue values of blue samples measured using ImageJ. (**C**) L*a*b*, RGB and Hue values of black samples measured using ImageJ. Errors taken as the standard deviation produced by the ImageJ software. Fits produced using MATLAB curve fitting toolbox.

12 References

1. Hunger, Klaus, ed. *Chp 7 Optical Brighteners*, *Industrial dyes: chemistry, properties, applications*. John Wiley & Sons, (2007).
2. Velapoldi, R., A. and Tønnesen, H.,H. Corrected Emission Spectra and Quantum Yields for a Series of Fluorescent Compounds in the Visible Spectral Region, *Journal of Fluorescence*, **14**, 465-472, (2004).
3. Pucci, A., Cuia, F. Di, Signori, F. & Ruggeri, G. Bis(benzoxazolyl)stilbene excimers as temperature and deformation sensors for biodegradable poly(1,4-butylene succinate) films. *J. Mater. Chem.* **17**, 783–790 (2006). (#23 in original manuscript)
4. European commission, COMMISSION REGULATION (EU) No 10/2011, *Off. J. Eur. Union*, 2011, https://eur-lex.europa.eu/legal-content/EN/TXT/PDF/?uri=CELEX:32011R0010&from=FR).
5. Fourati, M. A., Maris, T., Bazuin, C. G. & Prud'homme, R. E. (E)-4,4'-Bis(1,3 benzoxazol-2-yl)-stilbene at 150 and 375 K. *Acta Crystallogr. Sect. C* **66**, 11 14 (2010).
6. Kong, Y., Hay, J. N. Multiple melting behaviour of poly(ethylene terephthalate). *Polymer* **44**, 623–633 (2003).
7. Schyns, Z. O. G. & Shaver, M. P. Mechanical Recycling of Packaging Plastics: A Review. *Macromol. Rapid Commun.* 2000415 (2020).
8. ASTM F1249-20, "Standard Test Method for Water Vapor Transmission Rate Through Plastic Film and Sheeting Using a Modulated Infrared Sensor", (2020).
